# Reactivity of Carbohydrate Phosphodiesters, Potential Targets of Antibacterial Agents

**DOI:** 10.1002/cbdv.202501852

**Published:** 2025-08-28

**Authors:** Eero Sillanpää, Tuomas Lönnberg, Satu Mikkola

**Affiliations:** ^1^ Department of Chemistry University of Turku Turku Finland

**Keywords:** carbohydrates, catalysis, Cu(II) complexes phosphodiester bonds, reaction mechanisms

## Abstract

While monosaccharide units in bacterial carbohydrates may be linked with phosphodiester bonds, human carbohydrates exclusively contain glycosidic linkages. Differences between human and bacterial carbohydrates present potential targets for the development of novel antibacterial agents. The purpose of the present article is to study the reactivity of carbohydrate phosphodiester models to evaluate the possibility of a selective inactivation of bacterial carbohydrates within a biological matrix. The model compound was chosen for the ease of synthesis and the convenience of detection. Its reactivity was studied in the presence and in the absence of metal ion‐based catalysts. The results obtained show that the spontaneous reaction under neutral conditions is the glycoside hydrolysis, but metal catalysts enhance the cleavage by intramolecular transesterification. The reactivity was lower than that of the phosphodiester bonds of RNA under the same conditions. Corresponding phosphorothioates were synthesized to study the effect of the coordination of metal ion catalysts. However, unexpected reactions were observed, and in addition to glycoside hydrolysis, phosphorothioates reacted similarly to corresponding RNA analogs, resulting in the cleavage, phosphate migration, and desulphurization. Different metal ion catalysts steered the reaction in different directions.

## Graphical abstract

A simple carbohydrate phosphodiester model was synthesized to compare the reactivity of phosphodiester bonds within carbohydrates and RNA.

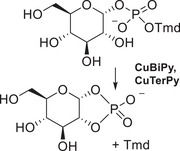

## 1 Introduction

Human and bacterial oligosaccharides differ from each other in that, in addition to glycosidic bonds, bacterial monosaccharides may also be linked *via* phosphodiester bonds. For example, certain serotypes of capsular polysaccharides from *Streptococcus pneumoniae* [1, 2, 3, 4], *Neisseria meningitidis* [5, 6, 7], and *Hemophilus influenzae* [8] contain phosphodiester‐linked carbohydrate units. Phosphodiester bonds are also common in cell‐surface teichoic acids, where monosaccharides may be linked by glycerol and ribitol phosphates [9, 10, 11, 12]. Phosphodiester‐linked carbohydrates and aminoalcohols are also found in the Lipid A unit in bacterial lipopolysaccharides (LPS) [13], and a diphosphate bridge may serve as a link between lipid A and the hydrocarbon chain that attaches the LPS to the cell surface, as in the case of *Klebsiella pneumoniae* [14].

Differences between human and bacterial carbohydrates present potential targets for therapeutic agents against bacterial infections, and one potential way of targeting a phosphodiester‐containing carbohydrate sequence is the cleavage of a phosphodiester bond within the structure. In this concept, the stability difference between phosphodiester bonds in bacterial carbohydrates and in human RNA is an essential factor. Information on the stability of the phosphodiester bond containing carbohydrates is also important for the development of carbohydrate conjugate vaccines, since their stability under storage conditions is essential for their safety and reliability [15, 16]. Aluminium‐based adjuvants, for example, have been observed to destabilize phosphodiester bond‐containing conjugate vaccines [17].

**SCHEME 1:**
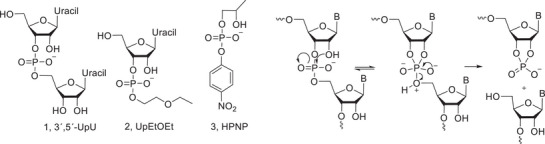
RNA model compounds and the transesterification of RNA phosphodiester bonds.

While the chemical reactivity of phosphodiester bonds of RNA has been extensively studied over the last decades [18], fairly little is known about the reactivity of phosphodiester bonds in carbohydrates. The structure of RNA is monotonous, and therefore the sequence of a linear RNA molecule has a relatively modest effect on the reactivity of phosphodiester bonds [18]. The structure of carbohydrates, in contrast, is much more varied, and larger reactivity differences can be expected. Studies with RNA model compounds, such as **1**–**3**, may, however, give information on the factors that influence the reactivity of phosphodiester bonds in general. Phosphodiester bonds in RNA are cleaved by intramolecular transesterification (Scheme 1), where a neighboring HO‐group attacks the phosphate, which results in the formation of a five‐membered cyclic phosphorane, and consequently in the cleavage of the phosphodiester bond [18]. Formation of a six‐membered cyclic phosphorane is clearly less favourable. It is also clear that the ribose ring in RNA places the attacking HO‐nucleophile in a favorable position for the nucleophilic attack that results in an in‐line geometry of the forming and breaking bonds [19, 20]. A nucleophile in a more flexible structure, such as the simple RNA model 2‐hydroxypropyl‐4‐nitrophenyl phosphate, is less efficient [21]. Departure of the leaving group nucleoside, a primary alcohol, is clearly the rate‐limiting step under neutral conditions [18].

Sugar nucleotides, such as uridine‐5‐diphosphate (UDP)‐glucose (**4**), form another class of model compounds for carbohydrate phosphodiesters, but their disadvantage is the good nucleotide leaving group, which affects the kinetics and thermodynamics of the transesterification. The results obtained have, however, shown that under neutral and basic conditions [22, 23, 24], as well as in the presence of metal ion‐based catalysts [25, 26], a 2‐OH group in *cis*‐position, as in UDP‐glucose (**4**), acts as a nucleophile that attacks the α‐phosphate. A nucleophile in *trans*‐position, as in GDP‐mannose (**5**), attacks preferably on C1 rather than on the phosphate group at C1 [26]. Under acidic conditions, sugar nucleotides react as glycosides, that is, by the release of the aglycon as a result of glycoside hydrolysis [22, 23].


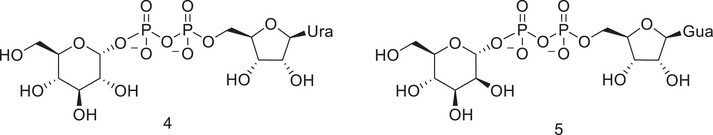


Studies on the structure and stability of carbohydrates, for example, in glycoconjugate vaccines often focus on hydrolytic cleavage [1, 2, 5, 27, 28]. These studies give valuable information on the stability of vaccines. However, information on the reactivity of phosphodiester bonds within the structures is often limited, particularly under acidic conditions where both glycosidic and phosphodiester bonds are cleaved. Under alkaline conditions, in contrast, glycosidic bonds are stable, and the observed reactivity can be attributed to the cleavage at a phosphodiester bond. Such studies are rather scarce, but the results reported show that under alkaline conditions, the reactivity of phosphodiester bonds follows the same basic rules as the reactivity of RNA phosphodiester bonds [27, 29]. A neighboring nucleophilic HO‐group is required for an efficient cleavage [30, 31], and the formation of a five‐membered ring is more favourable than the formation of a six‐membered ring. Furthermore, a nucleophilic attack by an HO‐group in a more flexible ribitol chain is less favourable than an attack by an HO‐group of ribose. The reactivity of all carbohydrate phosphodiesters can not, however, be explained on the basis of information derived from RNA models. For example, the *trans*‐oriented 2‐OH of rhamnose seems to be clearly more efficient nucleophile than a corresponding HO‐group in *cis‐*position [32]. Clearly, the more variable structures of carbohydrates may also result in an unpredictable reactivity. Furthermore, little is known about the effect of metal ion catalysts on the reactivity of carbohydrate phosphodiesters.

In this paper, we report on the synthesis and reaction kinetics of carbohydrate phosphodiester model **6α,** its anomer **6β**, and their phosphorothioate counterparts **7α** and **7β**. The aglycon is a thymidine nucleoside, that is, a ribose sugar attached to an ultraviolet (UV)‐active thymine base. This allows the detection by high‐performance liquid chromatography (HPLC) or by capillary electrophoresis equipped with a UV‐detector, which was used to monitor the cleavage of **6** and **7** in the absence and presence of Cu‐Bipyridine (CuBiPy, **8**) and Cu‐terpyridine (CuTerPy, **9**). We chose CuBiPy and CuTerPy because they are simple and well‐known complexes that are stable under neutral conditions. Furthermore, we have previously studied them as catalysts of the reactions of related compounds [21], which allows us to discuss their roles in the current work.


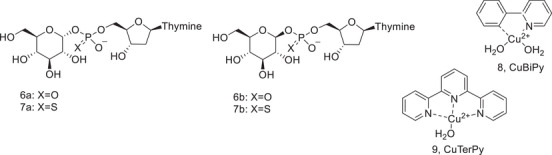


## 2 Results and Discussion

### 2.1 Synthesis and Analytical Methods

**SCHEME 2:**
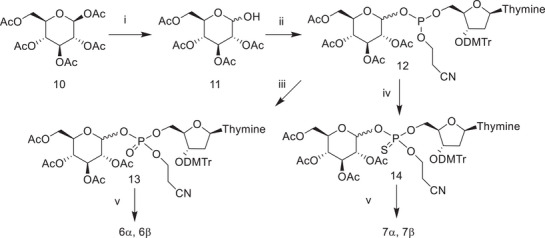
Synthesis of phosphodiesters **6** and phosphorothioates **7**. Reagents and conditions: i) hydrazine acetate, DMF ii) dT‐5′‐CE phosphoramidite, 5‐(benzylthio)‐1*H*‐tetrazole, MeCN iii) I_2_, H_2_O, pyridine iv) S_8_, CH_2_Cl_2_ v) 1. NH_3_, MeOH 2. TFA, CH_2_Cl_2_ 3. NH_3_, H_2_O.

We chose glucose 1‐phosphate derivatives as model compounds for the straightforward synthesis without the need for tedious protection and deprotection steps. As was discussed earlier, it was also known from previous studies with sugar nucleotides that under neutral and alkaline conditions, 2‐OH of glucose attacks on the phosphate, resulting in the substitution reaction at the phosphate group [23, 24, 26]. The synthetic route is shown in Scheme 2. β‐D‐glucose pentaacetate (**10**) was used as a starting material. The acetyl group at the anomeric position was selectively removed by treating with hydrazine acetate [33]. This resulted in the formation of a mixture of α‐ and β‐anomers of 2,3,4,6‐tetraacetyl glucose (**11**) that were coupled with deoxythymidine‐5′‐cyanoethyl phosphoramidite. The phosphite ester intermediate **12** was divided into two parts, one of which was oxidized with iodine in pyridine to the phosphate triester **13**, while the other part was treated with sulphur in dichloromethane to yield the phosphorothioate triester **14**. After the removal of the protecting groups, phosphodiesters **6α** and **6β**, as well as phosphorothioates **7α1, 7α2**, **7β1**, and **7β2**, were purified by semipreparative HPLC. Absolute configuration at the phosphorothioate group was not determined, but the α1, α2, β1, and β2 attributes refer to the faster and slower eluting diastereomers of the given α‐ or β‐anomer. Synthesis and characterization of the phosphodiesters and phosphorothioates are described in detail in the Experimental section, and a set of nuclear magnetic resonance (NMR) spectra is collected in the Supporting Information.

We studied the reactions of α‐ and β‐anomers of phosphodiester **6** and phosphorothioates at 90°C in the presence and in the absence of CuBiPy (**8**) and CuTerPy (**9**) catalysts. Aliquots withdrawn from the reaction solutions were analysed by reverse‐phase (RP)‐HPLC and/or capillary zone electrophoresis (CZE). As both polar and apolar products were expected, we employed two different analysis methods to ensure the detection and separation of all reaction components. Pseudo first‐order rate constants collected in Table 1 were calculated for the disappearance of the starting material. Analytical conditions utilized are described in the Experimental section, and representative chromatograms and electropherograms are collected in the Supporting Information.

**TABLE 1:** Pseudo first‐order rate constants of the cleavage of phosphodiesters and phosphorothioates^[a]^.

Substrate	*k*(NaOH)/	*k*(pH 3.0)/	*k*(pH 6.7)/	*k*(CuBiPy)/	*k*(CuTerPy)/
	10^−4^ s^−1^	10^−4^ s^−1^	10^−6^ s^−1^	10^−6^ s^−1^	10^−6^ s^−1^
α‐phosphate **6α**	1.58 ± 0.04	2.0 ± 0.1	0.98 ± 0.08	2.3 ± 0.5	7.1 ± 0.4
β‐phosphate **6β**		5.7 ± 0.4	2.6 ± 0.2	5 ± 1	6 ± 1
3′,5′‐UpU (**1**)^[b]^	45.1	0.024			
UpEtOEt (**12**)^[c]^			0.13	38	280
UDP‐glucose (**4**)^[d]^	1.78^[e]^		0.11^[f]^	1310^[g]^	125^[g]^
Thiophosphate **7α1**	1.45 ± 0.03		14 ± 1	560 ± 20	330 ± 80
Thiophosphate **7α2**	2.10 ± 0.06		11 ± 1	780 ± 80	450 ± 10
Thiophosphate **7β1**			75 ± 3	600 ± 30	650 ± 80
Thiophosphate **7β2**		7.5 ± 0.5	63 ± 8	770 ± 50	580 ± 60

^[a]^Values refer to 0.1 M NaOH, 10 mM CuBiPy, and CuTerPy at 90°C, except for data for **4**.

^[b]^From [34].

^[c]^From [35].

^[d]^From [26].

^[e]^10 mM NaOH at 50°C.

^[f]^pH 7.0 at 50°C.

^[g]^ 5 mM CuBiPy and CuTerPy at 50°C.

### 2.2 Reactions of Phosphodiesters 6α and 6β in the Absence of Cu Catalysts

**SCHEME 3:**
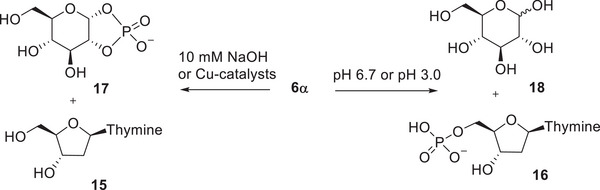
Reactions of **6α** under different experimental conditions.

We studied the reactivity of α‐ and β‐phosphates (**6α** and **6β**) in the absence of metal ion catalysts at three different pH levels at 90°C. In 0.1 M NaOH, only the α‐anomer was cleaved, and the only UV‐active product observed was thymidine (**15** in Scheme 3). In contrast, under neutral and slightly acidic conditions, thymidine 5’‐monophosphate (TMP, **16**) was formed as an initial product. The putative, UV‐inactive products **17** and **18** were not observed, as analytical methods based on UV‐detection were used.

The observations described above are consistent with results obtained previously with sugar nucleotides [22, 23, 24]. They show that the alkaline cleavage of **6α** proceeds by intramolecular transesterification, similarly to the alkaline cleavage of bacterial carbohydrate fragments described in the introduction. Under neutral and slightly acidic conditions, both anomers (**6α** and **6β**) react via glycoside hydrolysis, with the β‐anomer being slightly more reactive than its α‐counterpart.

The lack of transesterification under neutral conditions is consistent with the results obtained with two related compounds. A comparison between the alkaline reactivity of **6α** and 3’,5’‐UpU (**1**) [34] suggests that a nucleophilic attack by 2’‐OH of ribose on the neighboring 3’‐phosphate is approximately 30 times more efficient than the nucleophilic attack by glucose 2‐OH on the phosphate group at 1‐position. Assuming that the pH‐dependence of reactions of **6α** and **1** is similar, a rate constant of 2 × 10^−8^ s^−1^ can be estimated for the transesterification of **6α** at pH 6.7. Furthermore, a comparison to the reactivity of a sugar nucleotide UDP‐glucose (**4**) [23] suggests a 400‐fold reactivity difference between the base‐catalyzed transesterification reactions of **6α** and **4** at 90°C. A rate constant of 1 × 10^−5^ s^−1^ can be estimated for the transesterification of **4** at pH 6.7 and 90°C by extrapolation from the pH‐rate profile reported [23]. If the pH‐dependence of the transesterification reactions of **6α** and **4** are similar, this comparison gives almost the same estimate (2.5 × 10^−8^ s^−1^) as that obtained above with **6α** and 1.

The rate constants obtained for the hydrolysis of α‐ and β‐phosphates 6α and 6β under slightly acidic and neutral conditions are also well consistent with the reactivity of UDP‐glucose (**4**) [23, 24]. While UDP‐glucose undergoes a concurrent nucleophilic substitution that significantly contributes to the reactivity under neutral conditions, rate constants obtained for the glycoside hydrolysis under acidic conditions allow extrapolation to neutral conditions. The value of 1 × 10^−6^ s^−1^ thus estimated at pH 6.7, is practically the same as that obtained for 6α under the same conditions (Table 1).

### 2.3 Reactions of Phosphodiesters 6α and 6β in the Presence of Cu Catalysts

CuTerPy (**8**) and CuBiPy (**9**) enhance the reactions of phosphodiester **6α** very modestly. The apparent rate enhancement in comparison to the uncatalysed reaction at pH 6.7 is only 2‐ to 7‐fold. The main product in the presence of CuTerPy is thymidine (**15**), but an experiment with 5’‐TMP showed that CuTerPy does not promote the hydrolysis of 5’‐TMP. Together with the modest rate enhancement observed, these results suggest that CuTerPy enhances the transesterification of **6α** to an extent that it competes with the glycoside hydrolysis that is the prevailing reaction in the absence of Cu catalysts. A comparison to the estimated rate constant of 2 × 10^−8^ s^−1^ for the uncatalysed transesterification under the experimental conditions suggests an approximately 350‐fold rate‐enhancement by 10 mM CuTerPy at pH 6.7. However, results obtained previously with an RNA model **2** [35] suggest that a phosphodiester bond in RNA would most probably be at least 6 times more reactive under the same conditions. Comparison to results obtained with UDP‐glucose (**4**) with the same nucleophile [23] shows that the rate enhancement in the case of a sugar nucleotide is much more significant, which most probably results from a better leaving group as well as stronger binding of the catalyst with a diphosphate moiety of sugar nucleotide **4**.

The rate enhancement in the presence of CuBiPy is even more modest than in the presence of CuTerPy, and the reaction of **6α** produced equal amounts of TMP and thymidine. These results do not allow strict conclusions, but it is possible that CuBiPy very modestly enhances both the transesterification and the glycoside hydrolysis. As is shown by the results collected in Table 1, the glycoside hydrolysis of the β‐phosphate **6β** is enhanced approximately to the same extent as the reaction of **6α**. As discussed later, the rate enhancement of the glycoside hydrolysis by CuBiPy is seen more clearly with phosphorothioates as substrates

### 2.4 Reactions of Phosphorothioates in the Absence of Cu Catalysts

We synthesized phosphorothioate analogues **7α1**, **7α2, 7β1**, and **7β2** to study the effect of the binding of the metal ion catalyst on catalytic activity. However, even in the absence of catalysts, it became apparent that the reactions of α‐anomers **7α1** and **7α2** followed different reaction routes from those of phosphate **6α**. With β‐phosphorothioates **7β**, the product distribution under neutral conditions was simple and consistent with glycoside hydrolysis: a single product was initially formed under neutral conditions that subsequently decomposed, yielding thymidine. A known standard was not available, but the HPLC‐mass spectrometry (HPLC‐MS) analysis was consistent with the formation of thio‐TMP (**19**) as the initial product. Furthermore, the same product was formed in the reaction of β‐phosphorothioate **7β2** at pH 3.0, where the glucoside hydrolysis could be expected to be the predominant reaction of a glycoside with no intramolecular nucleophile. The only other product observed to any significant extent at pH 3.0 was thymidine, which was clearly formed as a secondary product. Traces of the corresponding phosphodiester **6β** and 5’‐TMP suggested desulphurization as a minor reaction route under these conditions.

**FIGURE 1:**
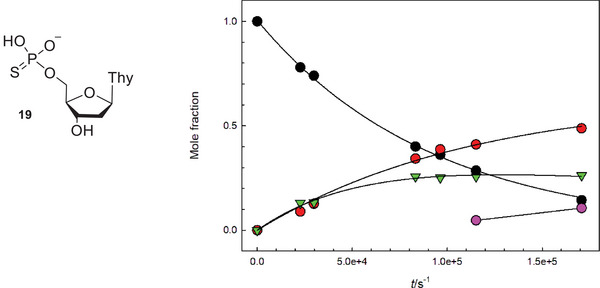
Product distribution curve in the reaction of phosphorothioate **7α2** at pH 6.7 at 90°C. Notation: **7α2 –** black circles; thymidine (**15**)**—**red circles; Unknown product**—**green triangles; 5’‐TMP (**16**)**—**purple circles. The curves have been calculated by fitting the mole fraction of the starting material **7α2** according to the equation of exponential decay, and the mole fractions of thymidine (**15**) and the unknown product according to the rate law of parallel and consecutive first‐order reactions [36].

Reactions of the α‐anomers **7α1** and **7α2** at 6.7 were slightly slower than those of their β‐anomers, and resulted in a more complicated product distribution, suggesting that the glycoside hydrolysis was a minor reaction pathway. The product identified above as thio‐TMP (**19**) was barely detected, and the main products were thymidine and a product that in CZE‐analysis migrated very close to the starting material. In HPLC analysis, the retention time of the unknown product was clearly shorter than that of the phosphorothioates **7α1** and **7α2**, which allowed a more reliable detection and quantification. The product distribution curves obtained in a reaction of **7α2** (Figure 1) showed that initially thymidine and the unknown product were formed in parallel, and approximately at the same rate. On a longer reaction time, the concentration of the unknown product started to level off. While the corresponding phosphodiester **6α** would be a potential intermediate, co‐injection with a known standard in an HPLC analysis showed that the unknown product was not the phosphodiester analog **6α**. Formation of TMP was observed at a longer reaction time.

**SCHEME 4:**
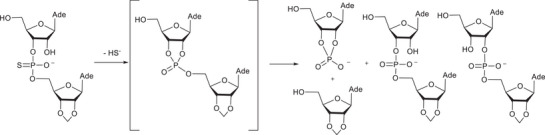
Nucleophilic reactions of a thio analog of an RNA model [38].

A potential explanation for the unexpected behavior of the α‐phosphorothioates can be evoked by studying the information obtained with related reaction systems. It is known that phosphorothioate analogs of dinucleoside monophosphates undergo three different reactions as a result of a nucleophilic attack of the adjacent 2′‐OH on the thiophosphate group: desulphurization, phosphate migration, and the cleavage of a phosphodiester bond [37, 38]. According to the studies with dinucleoside monophosphate analogs, the nucleophilic attack by the neighboring HO‐group on the thiophosphate group results initially in a rapid desulphurization and the formation of a cyclic phosphotriester intermediate (Scheme 4). The stable products are formed by the decomposition of endo‐ and exocyclic P─O –bonds in the intermediate.

**SCHEME 5:**
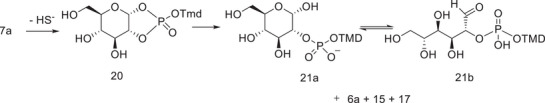
A potential reaction route for the spontaneous decomposition of thiophosphate **7α** based on the analogy to a phosphorothioate diester system [38].

Assuming that phosphothioates **7α** undergo a similar reaction sequence as described above, the products formed through a corresponding triester intermediate (**20** in Scheme 5) are phosphodiester **6α** and its 2‐isomer **21a**, as well as cleavage products thymidine and cyclic phosphodiester **17**. Phosphodiester **6α** was not observed, but the formation of thymidine as an initial product is consistent with such an analogous reaction. On the basis of analogy, the unknown product observed could be the 2‐isomer **21a**. The product distribution in the present case is different from that observed previously with diadenosine phosphorothioate in Scheme 4. This is conceivable considering that phosphotriester intermediates in Schemes 4 and 5 are significantly different. There are three esterified alcohols in the phosphotriester intermediate in Scheme 4, and it decomposes to form two isomeric acyclic phosphodiesters as well as one 2′,3′‐cylic phosphodiester. In contrast to this, corresponding phosphotriester **20** is asymmetric, as one of the endocyclic P─O –bonds is formed by a hemiacetal HO‐group. Therefore, the reactivity of the endocyclic P─O –bonds may well be different. Independent of the reactivity, the cleavage of endocyclic P─O bonds of **20** yields a mixture of two chemically different compounds: **6α** is a glycoside phosphate, but its isomer **21a** is a hemiacetal with a phosphodiester bond at an adjacent carbon (Scheme 5).

The structure of the open‐chain form **21b** offers a potential explanation for the formation of 5‘‐TMP at a later stage of the reaction**. 21b** is an analog of reducing sugar nucleotides, and its reactivity is more diverse than phosphodiesters in general. We have shown in our previous work that reducing sugar nucleotides, such as ribose‐5′‐UDP, undergo a phosphate elimination as a result of a sequence of keto‐enol equilibria [24]. A corresponding elimination reaction in **21b** is a potential explanation for the formation of 5‐TMP in the present reaction.

### 2.5 Reactions of Phosphorothioates in the Presence of Cu Catalysts

CuBiPy and CuTerPy promoted the decomposition of both α‐ and β‐anomers of phosphorothioates **7**. The rate enhancements were, however, modest: a 70‐fold rate enhancement of the decomposition of **7α2** was observed in the presence of 10 mM CuBiPy. Rate enhancement by CuTerPy was even more modest. The hydrolysis of the β‐anomers **7β** was also modestly enhanced, and an approximately 10‐fold rate acceleration was observed. There were no significant reactivity differences between the diastereomers of the same anomer.

**FIGURE 2:**
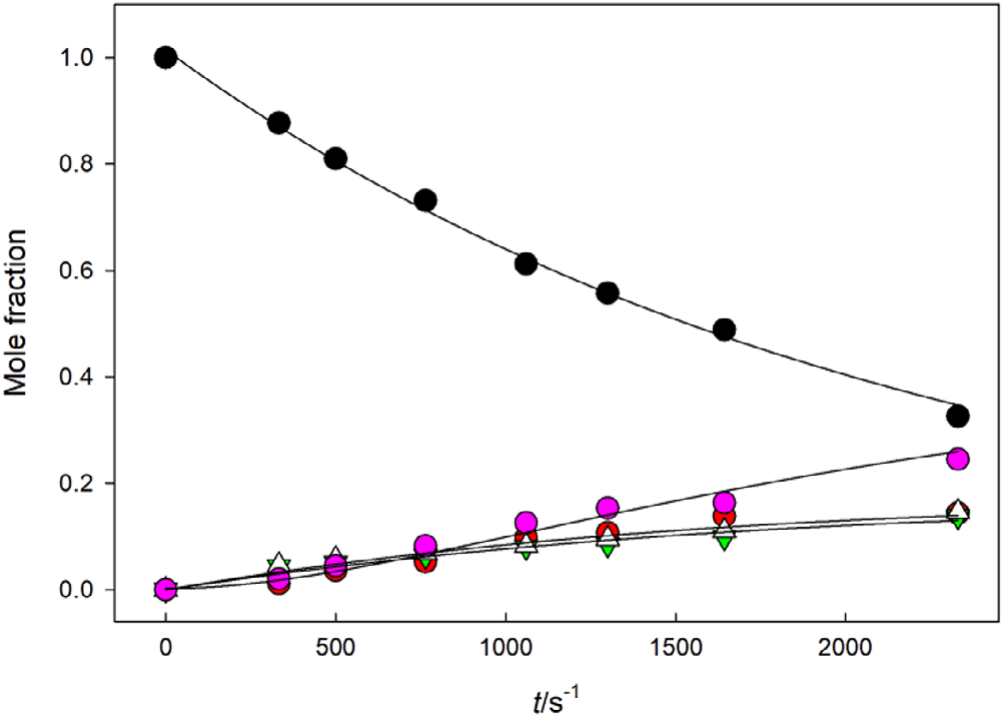
Product distribution curve in the reaction of phosphorothioate **7α2** in 10 mM CuBiPy solution at pH 6.7 at 90°C. Notation: **7α2 –** black circles; thymidine (**15**)**—**red circles; The putative isomerization product **20 –** green triangles; 5’‐TMP (**15**)**—**purple circles; Phosphodiester **6α—**white triangles. The curves have been calculated by fitting the mole fraction of the starting material **7α2** according to the equation of exponential decay, and the mole fractions of **20**, 5’‐TMP (**15**), and phosphodiester **6α** according to the rate law of parallel and consecutive first‐order reactions [36].

In the presence of 10 mM CuBiPy, the cleavage of both α‐ and β‐anomers of phosphothioates yielded TMP as a product. With β‐anomers, TMP was the only product observed, suggesting that CuBiPy enhanced the glycoside hydrolysis. As thio‐TMP did not accumulate in the presence of CuBiPy, it seems likely that CuBiPy efficiently enhanced also the desulphurization of a monophosphate, even though desulphurization of β‐phosphorothioates **7β1** and **7β2** was not observed to any significant extent. In the case of the α‐anomers **7α1** and **7α2,** the rate enhancement was larger than with their β‐counterparts. The product distribution was also more complex, as is shown by Figure 2 for the CuBiPy‐promoted reaction. TMP, thymidine, a product that was identified as the corresponding phosphodiester **6α,** and the putative phosphate migration product **21** were formed. These four products were formed concomitantly, and to almost the same extent. On a longer reaction time, the proportion of **21** slightly decreased, and that of 5’‐TMP increased. After two half‐lives of the reaction **7α2**, the proportion of 5’‐TMP was 36%.

The CuTerPy‐promoted reactions were slightly slower than reactions in the presence of CuBiPy. The reaction of α‐phosphorothioates **7α1** and **7α2** produced thymidine as almost the sole product. Some 5’‐TMP was observed on a longer reaction time, but the putative isomerization product **21** was not observed. The reaction of the β‐anomers produced TMP as the only product, which further proved that the decomposition of TMP is not enhanced by CuTerPy under the reaction conditions. Hence, thymidine in the reactions of α‐phosphorothioates **7α1** and **7α2** was not formed through the hydrolysis of TMP, but another reaction route was followed. The small amount of TMP was observed on a longer reaction time, suggesting that a slower reaction, most probably glycoside hydrolysis, producing TMP, took place concomitant with the reaction producing thymidine.

Cu‐complex promoted reactions of thiophosphates **7α** differ from those of the corresponding phosphate **6α** in that CuBiPy is a more efficient catalyst in the reaction of **7α** than CuTerPy is. Furthermore, both CuBiPy and CuTerPy affect the product distribution, but the effects are different. Different catalytic strategies most probably explain both observations. According to the previous analysis on the catalysis of phosphodiester cleavage by CuBiPy and CuTerPy, CuTerPy is more efficient in promoting the departure of the leaving group [21]. CuBiPy, in contrast, is more efficient in promoting the nucleophilic attack on the phosphate than CuTerPy is. The observation that CuTerPy favours the formation of thymidine is therefore logical. In the presence of CuBiPy, four products are formed at approximately the same rate. 5‘‐TMP is most probably produced by the glycoside hydrolysis. The rest of the products are formed at comparable rates through the cleavage of endo‐ and exocyclic P─O –bonds in the intermediate **20**. Apparently, CuBiPy is more efficient in promoting the cleavage of endocyclic P─O –bonds than CuTerPy. These observations are also consistent with our previous analysis [21], and suggest that CuBiPy promotes the nucleophilic attack on the phosphotriester intermediate either by mere coordination to the phosphate group, or by coordination to the attacking water molecule or hydroxide ion. The fact that the product distributions of the reactions in the absence and in the presence of CuBiPy are different supports the latter alternative, where the role of CuBiPy is more active.

Different catalytic mechanisms also explain the observation that CuBiPy is a more efficient catalyst for the reaction of **7α** than CuTerPy is, even though an opposite order was observed with phosphodiester **6α**. The cleavage of **6α** is a transesterification reaction with a rate‐limiting decomposition of a phosphorane intermediate. As our previous analysis shows [21], CuTerPy is more efficient than CuBiPy for this kind of reaction. The reaction of **7α** proceeds through a reactive phosphotriester intermediate in the absence and in the presence of CuBiPy. The reaction route is, hence, different, and it is well possible that the nucleophilic attack is the rate‐limiting step in this case, favoring CuBiPy as the catalyst.

## 3 Conclusions

Our original aim was to synthesize simple carbohydrate phosphodiesters and to study the reactivity of phosphodiester bonds in bacterial carbohydrates. To simplify the synthesis, a compound with a phosphodiester bond at the anomeric position was chosen as the target in the studies. However, the reactions of glycosides, particularly in combination with the thiophosphate linkage, turned out to be quite complex, and the information on the catalysis by metal complexes on the transesterification reaction remains fairly limited.

The rate enhancement of the intramolecular transesterification of a phosphodiester bond at the 1‐position of a glucose moiety by CuTerPy and CuBiPy could be estimated on the basis of the data obtained. The 350‐fold rate enhancement is, however, very modest, considering that under the same conditions, the cleavage of an RNA phosphodiester bond is enhanced by a factor of 2000 [35]. Ours is just a simple model, with one type of phosphodiester bond, and with simple catalysts, but it shows that, at least in this case, the carbohydrate phosphodiester bond would not be selectively cleaved in the presence of phosphodiester bonds of RNA.

Reactions of the phosphorothioates produced transesterification and glycoside hydrolysis products both in the presence and in the absence of CuBiPy and CuTerPy. The reaction route resulting in transesterification products seems, however, different from that of phosphodiesters. As a consequence, different product distributions are observed depending on the reaction conditions. Configuration at the phosphorothioate linkage does not have any significant effect on the rate enhancement by the Cu‐catalysts or the product distribution.

### 3.1 Experimental

#### 3.1.1 Synthesis of α‐ And β‐D‐glucos‐1‐yl Thymidin‐5′‐yl Phosphate (**6α** and **6β**)

Thymidine‐5′‐cyanoethyl phosphoramidite building block (0.307 g, 0.412 mmol) and 2,3,4,5‐tetra‐*O*‐acetyl‐d‐glucose (0.287 g, 0.824 mmol) were dissolved in anhydrous MeCN (2.75 mL). 0.3 M solution of 5‐(benzylthio)‐1*H*‐tetrazole (2.75 mL) was added, and the resulting mixture was stirred under N_2_ atmosphere at room temperature for 30 min, after which it was divided into two equal portions. 0.05 M solution of I_2_ in a mixture of pyridine and H_2_O (90:10, v/v, 16.5 mL) was added to one portion, and the resulting mixture was allowed to react at room temperature for 5 min. CH_2_Cl_2_ (50 mL) was added, and the resulting solution was washed with 0.2 M aqueous NaS_2_O_3_ (50 mL), dried over Na_2_SO_4_, and evaporated to dryness. The residue was dissolved in 7 M methanolic NH_3_ (1 mL), and the resulting mixture was incubated at room temperature for 1 h, after which it was evaporated to dryness. The residue was passed through a silica gel column by eluting with a mixture of Et_3_N, MeOH, and CH_2_Cl_2_ (1:25:74, v/v/v). The protected phosphodiester intermediate **13** thus obtained was dissolved in CH_2_Cl_2_ (2 mL). TFA (100 µL) was added, resulting in an immediate orange color indicative of the release of the dimethoxytrityl cation. The mixture was then neutralized by the addition of Et_3_N (200 µL) and evaporated to dryness. The residue was dissolved in 25% aqueous NH_3_ (1 mL) and allowed to react for 30 min, after which the mixture was neutralized by the addition of AcOH. Finally, the product mixture was fractioned by RP‐HPLC on a Hypersil ODS C18 column (250 × 10 mm, 5 µm) eluting with a mixture of MeCN and 50 mM aqueous ammonium formate (1:99, v/v) at a flow rate of 3 mL/min. Yields of the α and β anomers **6α** and **6β** were determined spectrophotometrically as 0.156 µmol (0.0754%) and 0.248 µmol (0.120%), respectively.

^1^H NMR (**6α**, 500 MHz, D_2_O) δ 7.75 (s, 1H; Thd‐H6), 6.35 (t, *J* = 6.9 Hz, 1H; Thd‐H1′), 5.50 (dd, *J* = 3.6, 7.3 Hz, 1H; Glu‐H1), 4.59 (m, 1H; Thd‐H3′), 4.18 (m, 1H; Thd‐H4′), 4.14 (m, 2H; Thd‐H5′ and H5″), 3.80 (m, 1H; Glu‐H5), 3.77 (m, 2H; Glu‐H6 and H6′), 3.73 (t, *J* = 9.4 Hz, 1H; Glu‐H3), 3.56 (m, 1H; Glu‐H2), 3.47 (t, *J* = 9.6 Hz, 1H; Glu‐H4), 2.37 (dd, *J* = 4.8, 6.7 Hz, 2H; Thd‐H2′and H2″), 1.93 (s, 3H; Thd‐CH_3_). ^13^C NMR (**6α**, 126 MHz, D_2_O) δ 166.5 (Thd‐C4), 151.7 (Thd‐C2), 137.3 (Thd‐C6), 111.6 (Thd‐C5), 95.4 (d, *J* = 6.4 Hz, Glu‐C1), 85.4 (d, *J* = 9.1 Hz, Thd‐C4′), 85.1 (Thd‐C1′), 72.8 (Glu‐C3), 72.5 (Glu‐C5), 71.3 (d, *J* = 7.6 Hz, Glu‐C2), 71.0 (Thd‐C3′), 69.1 (Glu‐C4), 65.1 (d, *J* = 7.1 Hz, Thd‐C5′), 60.2 (Glu‐C6), 38.7 (Thd‐C2′), 11.6 (Thd‐CH_3_). ^31^P NMR (**6α**, 202 MHz, D_2_O) δ ‐1.7.^1^H NMR (**6β**, 500 MHz, D_2_O) δ 7.75 (s, 1H; Thd‐H6), 6.36 (t, *J* = 7.1 Hz, 1H; Thd‐H1′), 4.92 (t, *J* = 7.8 Hz, 1H; Glu‐H1), 4.60 (m, 1H; Thd‐H3′), 4.18 (m, 1H; Thd‐H4′), 4.18–4.10 (m, 2H; Thd‐H5′ and H5″), 3.89 (m, 1H; Glu‐H6), 3.71 (dd, *J* = 5.8, 12.4 Hz, 1H; Glu‐H6′), 3.51 (t, *J* = 9.2 Hz, 1H; Glu‐H3), 3.47 (m, 1H; Glu‐H5), 3.39 (t, *J* = 9.5 Hz, 1H; Glu‐H4), 3.34 (t, *J* = 8.6 Hz, 1H; Glu‐H2), 2.37 (m, 2H; Thd‐H2′and H2″), 1.94 (s, 1H; Thd‐CH_3_). ^13^C NMR (**6β**, 126 MHz, D_2_O) δ 166.6 (Thd‐C4), 151.8 (Thd‐C2), 137.4 (Thd‐C6), 111.7 (Thd‐C5), 97.7 (d, *J* = 6.4 Hz, Glu‐C1), 85.4 (d, *J* = 9.1 Hz, Thd‐C4′), 85.0 (Thd‐C1′), 76.4 (Glu‐C5), 75.3 (Glu‐C3), 73.5 (d, *J* = 8.6 Hz, Glu‐C2), 71.1 (Thd‐C3′), 69.4 (Glu‐C4), 65.3 (d, *J* = 6.0 Hz, Thd‐C5′), 60.7 (Glu‐C6), 38.7 (Thd‐C2′), 11.7 (Thd‐CH_3_). ^31^P NMR (**6β**, 202 MHz, D_2_O) δ ‐1.9. HRMS (ESI–TOF): *m*/*z* calcd for [C_16_H_24_N_2_O_13_P]: 483.10215; found: 483.10288 [M─H]^‐^.

#### 3.1.2 Synthesis of α‐ and β‐D‐glucos‐1‐yl Thymidin‐5′‐yl Phosphorothioate (**7α1**, **7α2**, **7β1** and **7β2**)

The other portion of the phosphite triester solution prepared above was diluted with CH_2_Cl_2_ (2 mL). S_8_ (0.39 g, 1.5 mmol) was added, and the resulting heterogeneous mixture was stirred at room temperature for 4 d, after which it was diluted with CH_2_Cl_2_ (50 mL), filtered, and washed with 1 M aqueous NaHCO_3_ (50 mL). The organic phase was evaporated to dryness, and the residue was dissolved in 7 M methanolic NH_3_ (1 mL). The resulting mixture was incubated at room temperature for 1 h, after which it was evaporated to dryness. The residue was passed through a silica gel column by eluting with a mixture of Et_3_N, MeOH, and CH_2_Cl_2_ (1:5:94, v/v/v). The protected phosphorothioate diester intermediate **14,** thus obtained, was dissolved in CH_2_Cl_2_ (2 mL). TFA (100 µL) was added, resulting in an immediate orange colour indicative of the release of the dimethoxytrityl cation. The mixture was then neutralized by the addition of Et_3_N (200 µL) and evaporated to dryness. The residue was dissolved in 25% aqueous NH_3_ (1 mL) and allowed to react for 30 min, after which the mixture was neutralized by the addition of AcOH. Finally, the product mixture was fractioned by RP‐HPLC on a Hypersil ODS C18 column (250 × 10 mm, 5 µm) eluting with a mixture of MeCN and 50 mM aqueous ammonium formate (2:98, v/v) at a flow rate of 3 mL/min. Yields of the faster‐ and slower‐eluting diastereomers of the α and β anomers (**7α1**, **7α2**, **7β1** and **7β2**) were determined spectrophotometrically as 1.18 µmol (0.573%), 1.21 µmol (0.587%), 0.725 µmol (0.352%), and 1.19 µmol (0.578%), respectively.

^1^H NMR (**7α1**, 500 MHz, D_2_O) δ 7.74 (d, *J* = 1.2 Hz, 1H; Thd‐H6), 6.33 (t, *J* = 7.0 Hz, 1H; Thd‐H1′), 5.66 (dd, *J* = 3.6, 9.2 Hz, 1H; Glu‐H1), 4.58 (m, 1H; Thd‐H3′), 4.21–4.15 (m, 3H; Thd‐H4′, H5′ and H5″), 3.79 (m, 1H; Glu‐H5), 3.76 (m, 2H; Glu‐H6 and H6′), 3.70 (t, *J* = 9.5 Hz, 1H; Glu‐H3), 3.57 (ddd, *J* = 2.6, 3.5, 9.8 Hz, 1H; Glu‐H2), 3.46 (t, *J* = 9.5 Hz, 1H; Glu‐H4), 2.35 (m, 2H; Thd‐H2′and H2″), 1.94 (d, *J* = 1.0 Hz, 1H; Thd‐CH_3_). ^13^C NMR (**7α1**, 126 MHz, D_2_O) δ 166.5 (Thd‐C4), 151.7 (Thd‐C2), 137.3 (Thd‐C6), 111.6 (Thd‐C5), 95.6 (d, *J* = 7.2 Hz, Glu‐C1), 85.2 (d, *J* = 9.3 Hz, Thd‐C4′), 85.1 (Thd‐C1′), 73.0 (Glu‐C3), 72.6 (Glu‐C5), 71.2 (d, *J* = 8.1 Hz, Glu‐C2), 71.0 (Thd‐C3′), 69.0 (Glu‐C4), 65.6 (d, *J* = 5.7 Hz, Thd‐C5′), 60.2 (Glu‐C6), 38.7 (Thd‐C2′), 11.8 (Thd‐CH_3_). ^31^P NMR (**7α1**, 202 MHz, D_2_O) δ 54.9.^1^H NMR (**7α2**, 500 MHz, D_2_O) δ 7.78 (s, 1H; Thd‐H6), 6.35 (t, *J* = 7.0 Hz, 1H; Thd‐H1′), 5.71 (dd, *J* = 3.5, 9.9 Hz, 1H; Glu‐H1), 4.59 (m, 1H; Thd‐H3′), 4.23–4.14 (m, 3H; Thd‐H4′, H5′ and H5″), 3.80 (m, 1H; Glu‐H5), 3.76 (m, 2H; Glu‐H6 and H6′), 3.74 (t, *J* = 9.6 Hz, 1H; Glu‐H3), 3.58 (m, 1H; Glu‐H2), 3.48 (t, *J* = 9.5 Hz, 1H; Glu‐H4), 2.37 (m, 2H; Thd‐H2′and H2″), 1.97 (s, 3H; Thd‐CH_3_). ^13^C NMR (**7α2**, 126 MHz, D_2_O) δ 166.6 (Thd‐C4), 151.7 (Thd‐C2), 137.3 (Thd‐C6), 111.7 (Thd‐C5), 96.0 (d, *J* = 7.1 Hz, Glu‐C1), 85.4 (d, *J* = 9.3 Hz, Thd‐C4′), 85.2 (Thd‐C1′), 73.2 (Glu‐C3), 72.7 (Glu‐C5), 71.31 (Glu‐C2), 71.25 (Thd‐C3′), 69.0 (Glu‐C4), 65.4 (d, *J* = 6.2 Hz, Thd‐C5′), 60.2 (Glu‐C6), 38.8 (Thd‐C2′), 11.7 (Thd‐CH_3_). ^31^P NMR (**7α2**, 202 MHz, D_2_O) δ 55.6.^1^H NMR (**7β1**, 500 MHz, D_2_O) δ 7.98 (s, 1H; Thd‐H6), 6.58 (t, *J* = 6.9 Hz, 1H; Thd‐H1′), 5.38 (dd, *J* = 8.1, 10.4 Hz, 1H; Glu‐H1), 4.84 (m, 1H; Thd‐H3′), 4.48–4.42 (m, 3H, Thd‐H4′, H5′ and H5″), 4.13 (dd, *J* = 2.2, 12.2 Hz, 1H, Glu‐H6), 4.06–4.02 (m, 1H, Glu‐H6′), 3.80 (t, *J* = 9.3 Hz, 1H; Glu‐H3), 3.73 (m, 1H; Glu‐H5), 3.70 (t, *J* = 9.5 Hz, 1H; Glu‐H4), 3.64 (t, *J* = 8.4, 1H; Glu‐H2), 2.63 (m, 2H; Thd‐H2′and H2″), 2.22 (br, 1H; Thd‐CH_3_). ^13^C NMR (**7β1**, 126 MHz, D_2_O) δ 166.6 (Thd‐C4), 151.9 (Thd‐C2), 137.6 (Thd‐C6), 111.8 (Thd‐C5), 98.5 (Glu‐C1), 85.6 (Thd‐C4′ and Thd‐C1′), 76.8 (Glu‐C5), 75.9 (Glu‐C3), 74.0 (Glu‐C2), 71.4 (Thd‐C3′), 69.8 (Glu‐C4), 66.1 (Thd‐C5′), 61.1 (Glu‐C6), 39.0 (Thd‐C2′), 12.0 (Thd‐CH_3_). ^31^P NMR (**7β1**, 202 MHz, D_2_O) δ 55.8. ^1^H NMR (**7β2**, 500 MHz, D_2_O) δ 7.79 (d, *J* = 1.2 Hz, 1H; Thd‐H6), 6.36 (t, *J* = 7.1 Hz, 1H; Thd‐H1′), 5.10 (dd, *J* = 7.9, 9.6 Hz, 1H; Glu‐H1), 4.61 (m, 1H; Thd‐H3′), 4.26–4.14 (m, 3H; Thd‐H4′, H5′ and H5″), 3.89 (dd, *J* = 2.2, 12.4 Hz, 1H; Glu‐H6), 3.72 (dd, *J* = 5.7, 12.5 Hz, 1H; Glu‐H6′), 3.53 (t, *J* = 9.3 Hz, 1H; Glu‐H3), 3.49 (m, 1H; Glu‐H5), 3.41 (t, *J* = 9.5 Hz, 1H; Glu‐H4), 3.37 (dd, *J* = 8.3, 9.1 Hz, 1H; Glu‐H2), 2.37 (m, 2H; Thd‐H2′and H2″), 1.96 (d, *J* = 1.1 Hz, 1H; Thd‐CH_3_). ^13^C NMR (**7β2**, 126 MHz, D_2_O) δ 166.6 (Thd‐C4), 151.7 (Thd‐C2), 137.4 (Thd‐C6), 111.7 (Thd‐C5), 97.7 (d, *J* = 7.0 Hz, Glu‐C1), 85.3 (d, *J* = 9.3 Hz, Thd‐C4′), 85.1 (Thd‐C1′), 76.4 (Glu‐C5), 75.4 (Glu‐C3), 73.4 (d, *J* = 8.7 Hz, Glu‐C2), 71.3 (Thd‐C3′), 69.3 (Glu‐C4), 65.8 (d, *J* = 6.2 Hz, Thd‐C5′), 60.6 (Glu‐C6), 38.7 (Thd‐C2′), 11.7 (Thd‐CH_3_). ^31^P NMR (**7β2**, 202 MHz, D_2_O) δ 55.4. HRMS (ESI–TOF): *m*/*z* calcd for [C_16_H_24_N_2_O_12_PS]: 499.07931; found: 483.07929 [M─H]^‐^.

#### 3.1.3 Kinetic Experiments

The pH of the reaction solutions was adjusted with 50 mM formic acid (pH 3.0) or MOPSO buffer (pH 6.7). The ionic strength was adjusted to 0.1 M with NaNO_3_. The reaction temperature was adjusted to 90°C and controlled either with a water bath (slow reactions) or an electric heating block (reactions with *t*_1/2_< 5 h). Typically, 10‐12 35 µl aliquots were withdrawn to cover two half‐lives of the reaction. Reactions were quenched by placing the aliquots on an ice‐salt bath. Aliquots were either analyzed immediately or stored in a freezer. NaOH‐catalyzed reactions were quenched by adding 1.0 M acetic acid solution.

HPLC analyses were carried out with a Perkin Elmer Flexar system equipped with an Aquasil C18 column (150 × 4 mm, particle size 5 µm). Mixtures of acetate buffer (50 mM, pH 4.3) and acetonitrile were used as eluents. Generally, 1.1% acetonitrile in acetic acid buffer was employed in the kinetic experiments. The flow rate was 1.5 mL/min. UV‐active compounds were detected at 268 nm. CZE analysis was carried out with HP ^3D^CE equipment in a fused silica capillary (77 cm effective length, i.d. 75 µm). MOPSO buffer (0.1 M, pH 6.7) was used as a background electrolyte. A voltage of 30 kV was applied. The equipment was thermostated to 25°C.

Rates for the disappearance of the starting material were calculated by applying the integrated first‐order rate law to the decrease of mole fraction or the signal area of the substrate as a function of time. Product distribution curves in Figures 1 and 2 have been obtained by fitting the mole fraction (x) of the starting material according to the equation of exponential decay and the mole fractions of products according to the rate law of parallel and consecutive first‐order reactions [36].

## Author Contributions

**Eero Sillanpää**: investigation; **Tuomas Lönnberg**: conceptualization, data curation, supervision, writing – review and editing; **Satu Mikkola**: conceptualization, data curation, formal analysis, investigation, supervision, writing – original draft, review and editing.

## Conflicts of Interest

The authors declare no conflicts of interest.

## Supporting information




**Supporting File 1**: cbdv70408‐sup‐0001‐SuppMat.pdf

## Data Availability

The data that support the findings of this study are available in the Supporting Information of this article.
