# Supplementary material for: Reactivity of Carbohydrate Phosphodiesters, Potential Targets of Antibacterial Agents
Source: Chem Biodivers. 2025 Aug 28;22(12):e01852. doi: 10.1002/cbdv.202501852 (PMC12716006; doi:10.1002/cbdv.202501852)
Supplement: Supplementary file 1 — Supporting File 1: cbdv70408‐sup‐0001‐SuppMat.pdf [file CBDV-22-e01852-s001.pdf]

Supporting information

Contents

Figure S1: ^{1}H NMR spectrum (500 MHz, D_2O) of compound **6α** Page S2
Figure S2: ^{13}C NMR spectrum (126 MHz, D_2O) of compound **6α** Page S4
Figure S3: ^{31}P NMR spectrum (202 MHz, D_2O) of compound **6α** Page S6
Figure S4: ^{1}H NMR spectrum (500 MHz, D_2O) of compound **6β** Page S7
Figure S5: ^{13}C NMR spectrum (126 MHz, D_2O) of compound **6β** Page S9
Figure S6: ^{31}P NMR spectrum (202 MHz, D_2O) of compound **6β** Page S11
Figure S7: ^{1}H NMR spectrum (500 MHz, D_2O) of compound **7α1** Page S12
Figure S8: ^{13}C NMR spectrum (126 MHz, D_2O) of compound **7α1** Page S14
Figure S9: ^{31}P NMR spectrum (202 MHz, D_2O) of compound **7α1** Page S16
Figure S10: ^{1}H NMR spectrum (500 MHz, D_2O) of compound **7α2** Page S17
Figure S11: ^{13}C NMR spectrum (126 MHz, D_2O) of compound **7α2** Page S19
Figure S12: ^{31}P NMR spectrum (202 MHz, D_2O) of compound **7α2** Page S21
Figure S13: ^{1}H NMR spectrum (500 MHz, D_2O) of compound **7β1** Page S22
Figure S14: ^{13}C NMR spectrum (126 MHz, D_2O) of compound **7β1** Page S24
Figure S15: ^{31}P NMR spectrum (202 MHz, D_2O) of compound **7β1** Page S26
Figure S16: ^{1}H NMR spectrum (500 MHz, D_2O) of compound **7β2** Page S27
Figure S17: ^{13}C NMR spectrum (126 MHz, D_2O) of compound **7β2** Page S29
Figure S18: ^{31}P NMR spectrum (202 MHz, D_2O) of compound **7β2** Page S31

Conditions of capillary electrophoresis and HPLC analysis Page S32

Figures S19: Electropherograms, α-phosphate **6α** at pH 6.7 Page S32
Figure S20: An electropherogram, α-phosphate **6α** at pH 3.0 Page S33
Figure S21: HPLC-chromatograms, α-phosphate **6α** with 10 mM CuBiPy Page S33
Figure S22: An HPLC-chromatogram, α-phosphate **6α** with 10 mM CuTerPy Page S34
Figure S23: HPLC-chromatogram, TMP with 10 mM CuTerPy Page S34
Figure S24: An electropherogram, **7β2** at pH 3.0 Page S35
Figure S25: An electropherogram, **7α2** at pH 6.7 Page S35
Figure S26: HPLC-chromatograms, **7α2** at pH 6.7 Page S36
Figure S27: HPLC-chromatogram, **A: 7α2** at pH 6.7. **B:** Spiked with α-phosphate **6α** Page S37
Figure 28: HPLC-chromatograms, **7α2** with 10 mM CuBiPy Page S38
Figure 29: HPLC-chromatograms, **7α2** with CuTerPy Page S38

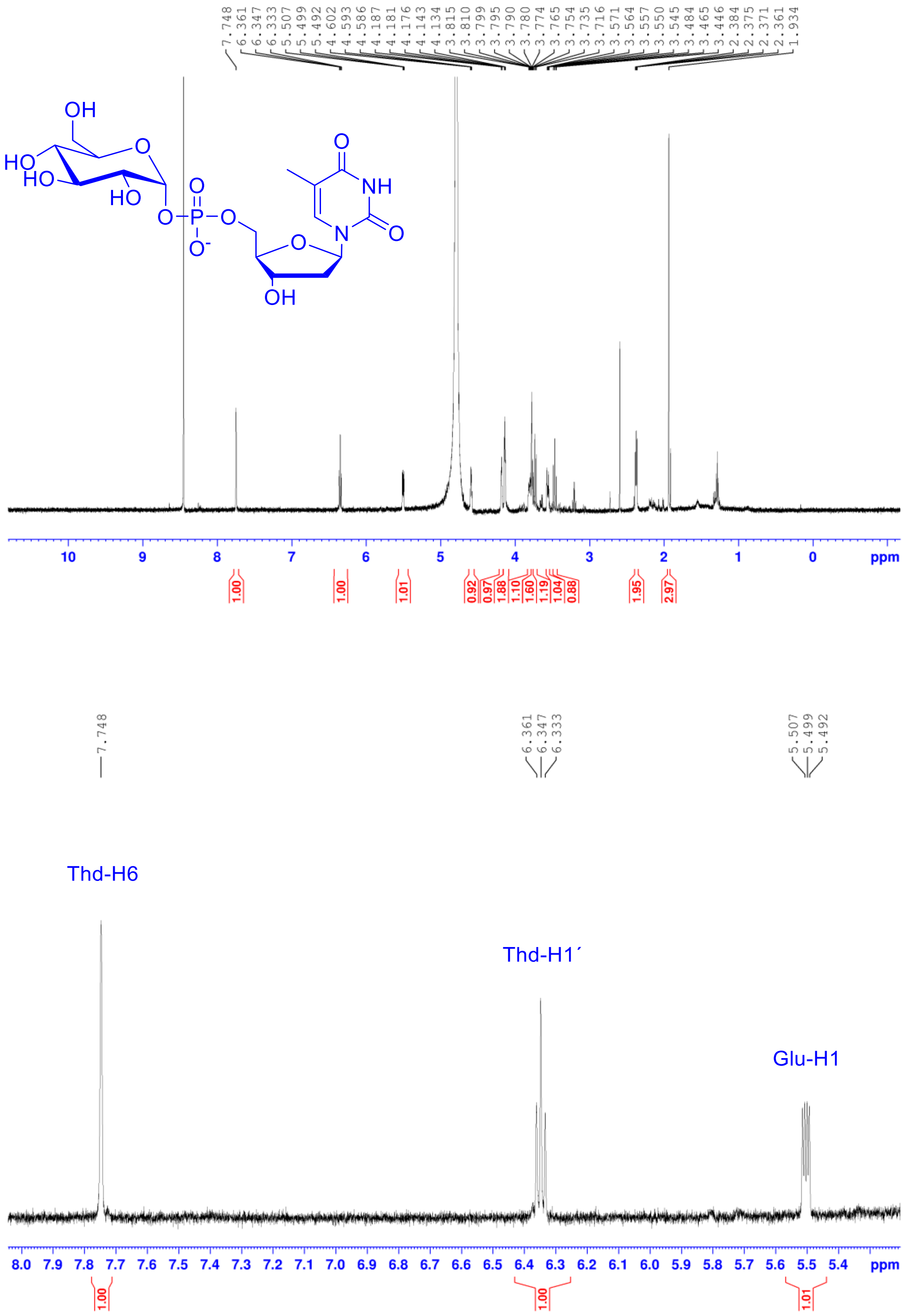

Figure S1. ^{1}H NMR spectrum (500 MHz, D_2O) of compound **6α**.

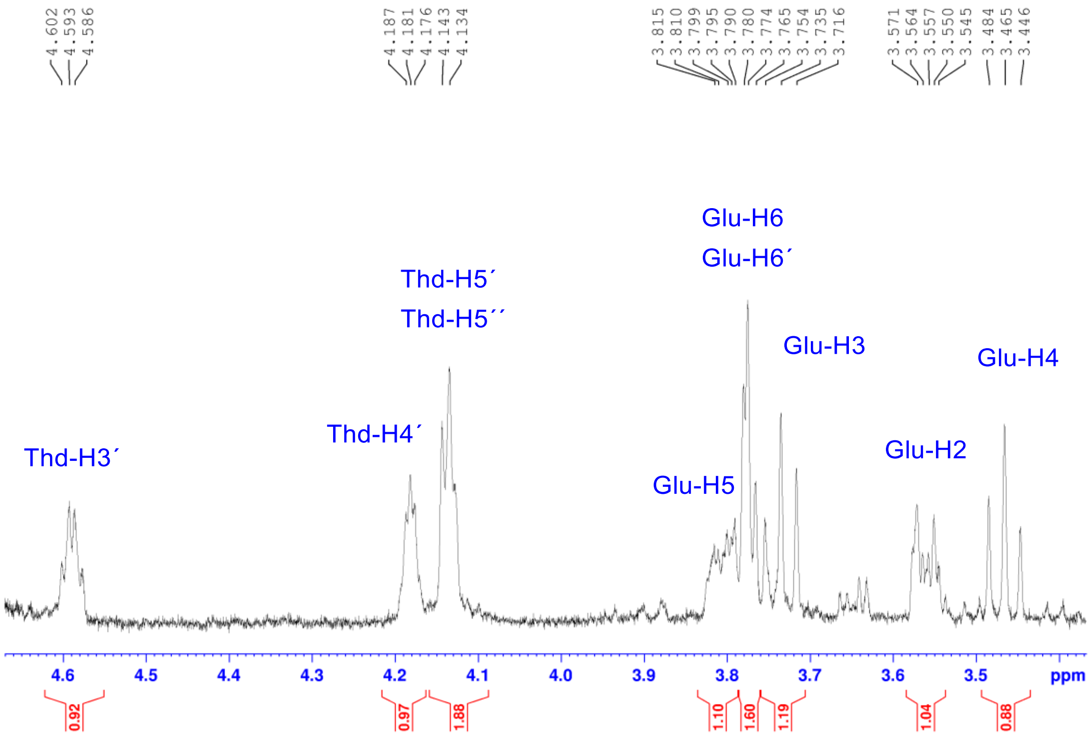

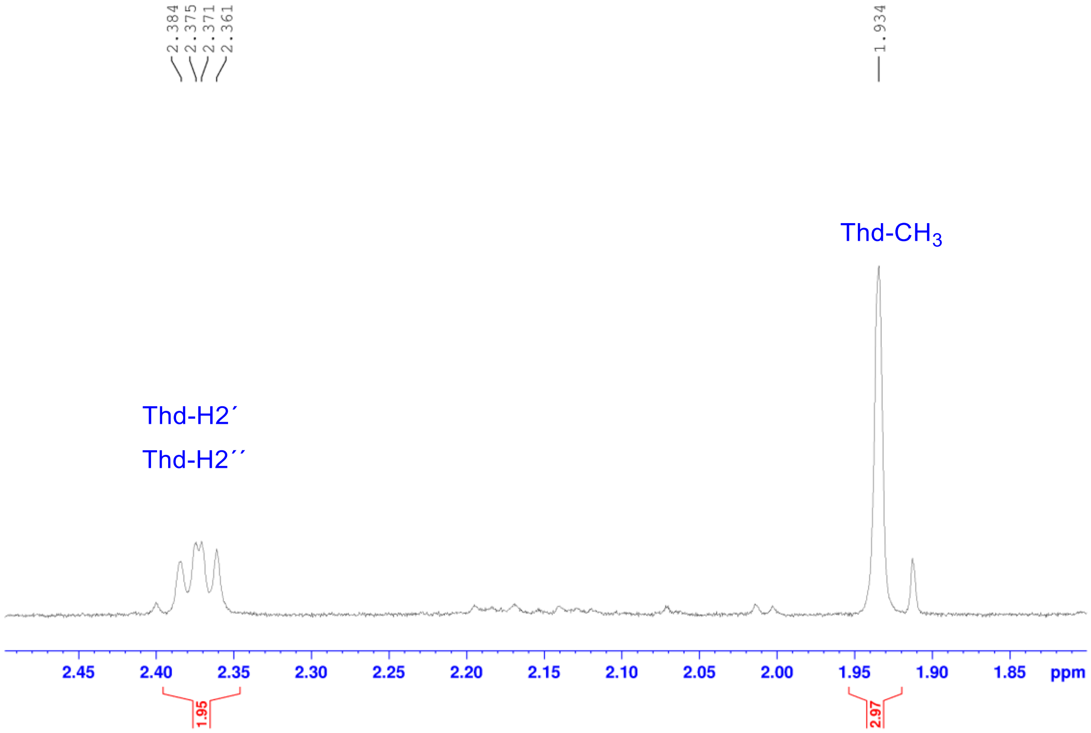

Figure S1 (continued). ^{1}H NMR spectrum (500 MHz, D_2O) of compound **6α**.

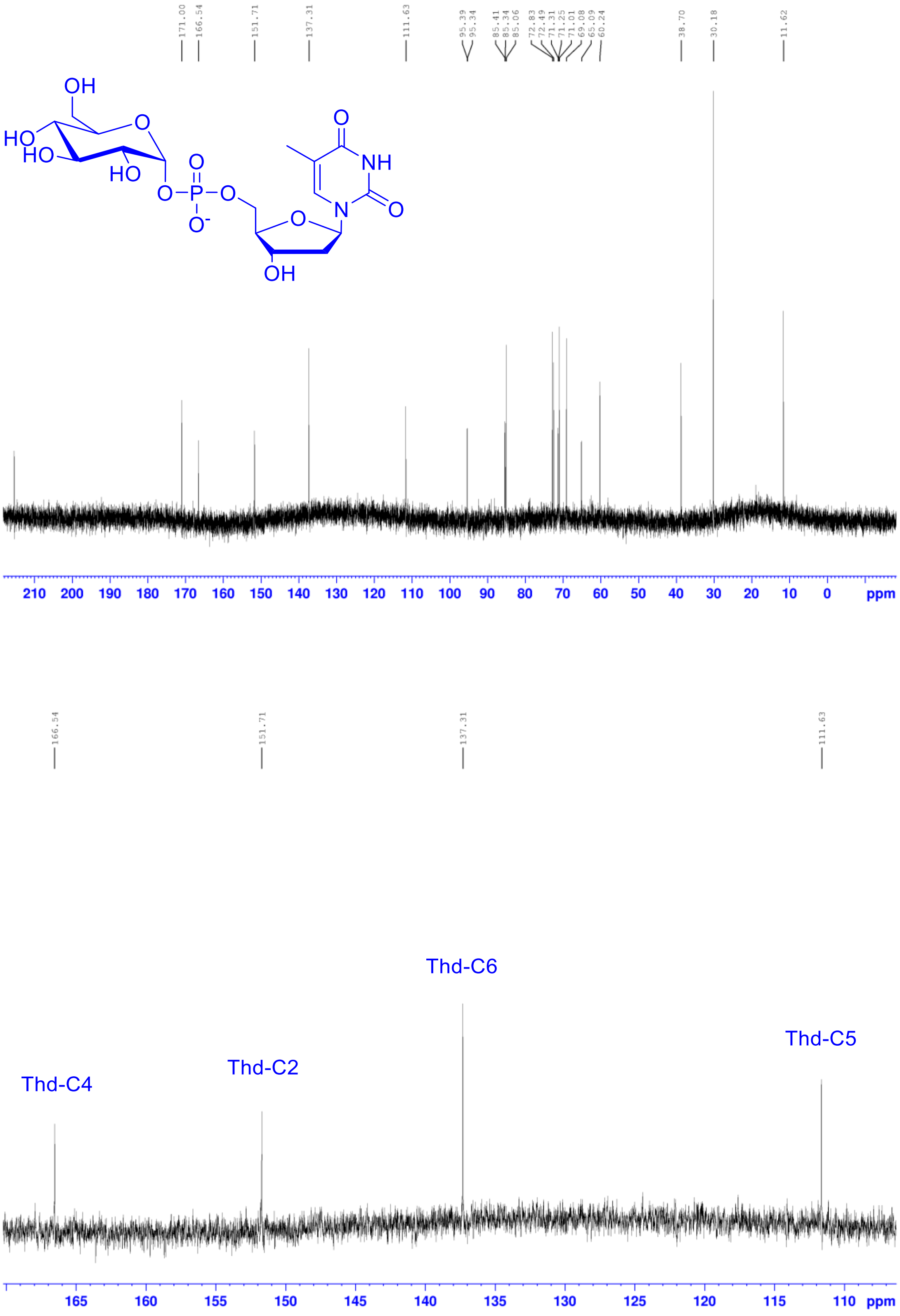

Figure S2. ^{13}C NMR spectrum (126 MHz, D_2O) of compound **6**α.

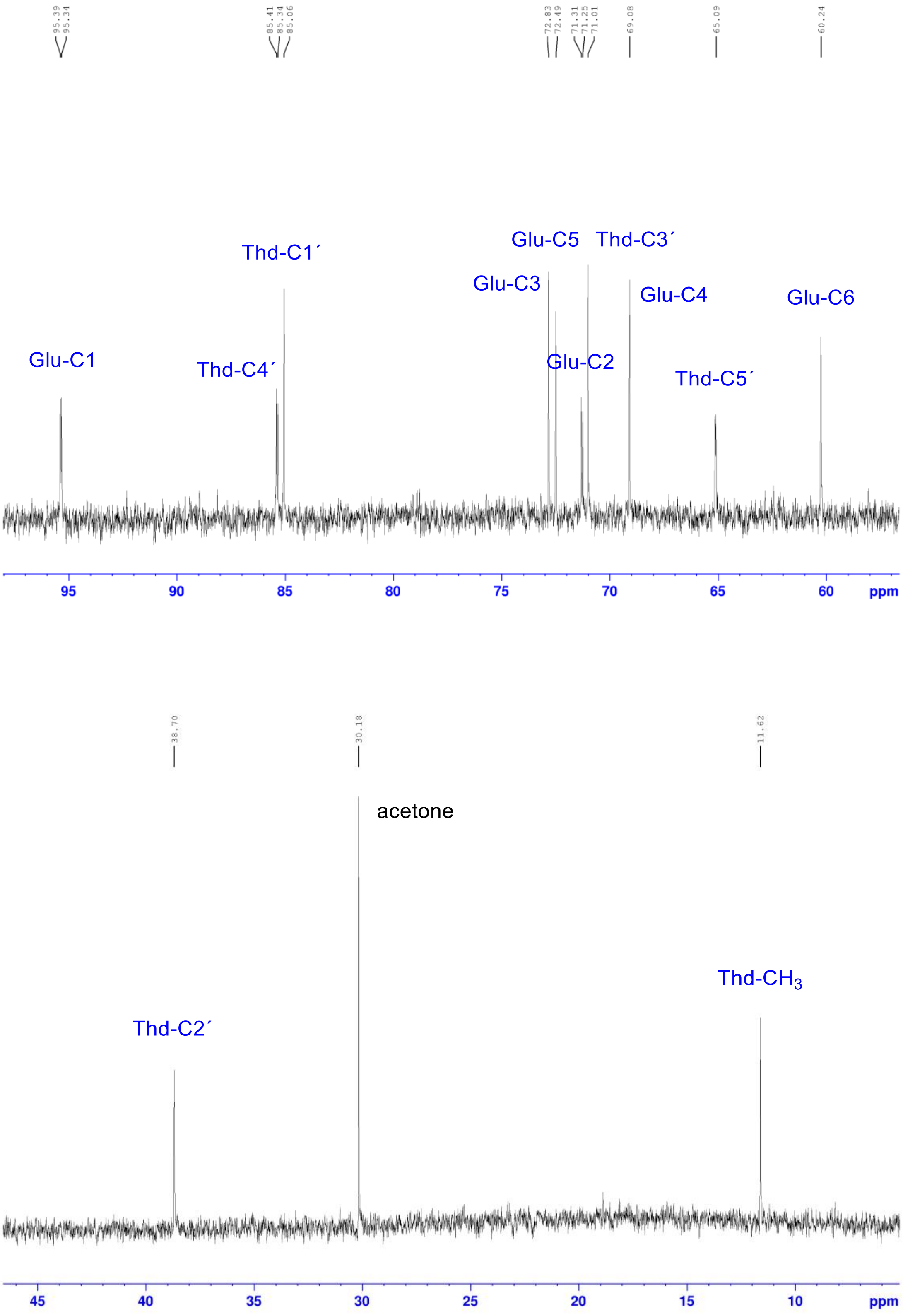

Figure S2 (continued). ^{13}C NMR spectrum (126 MHz, D_2O) of compound **6α.**

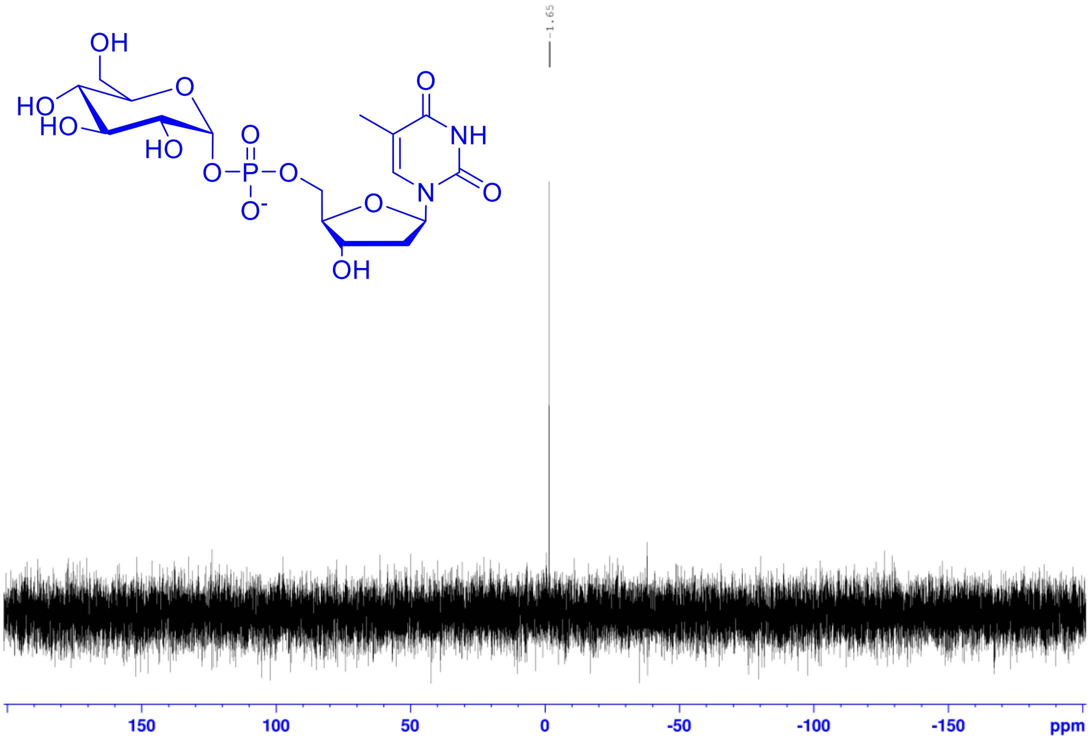

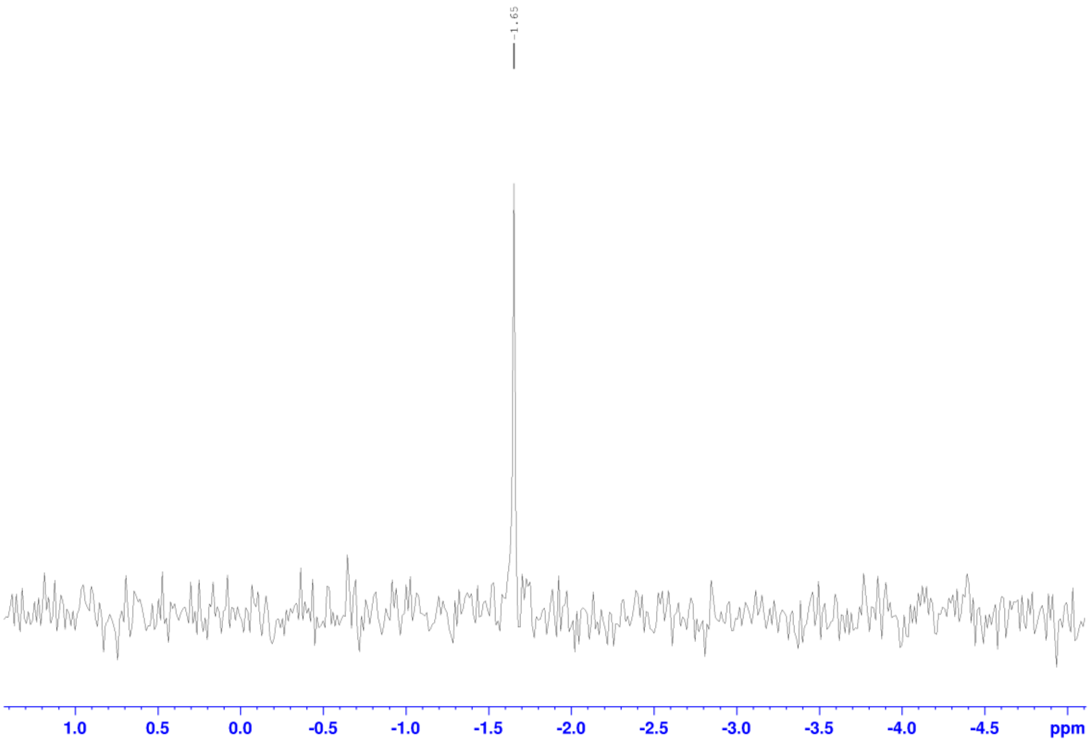

Figure S3. ^{31}P NMR spectrum (202 MHz, D_2O) of compound **6α**.

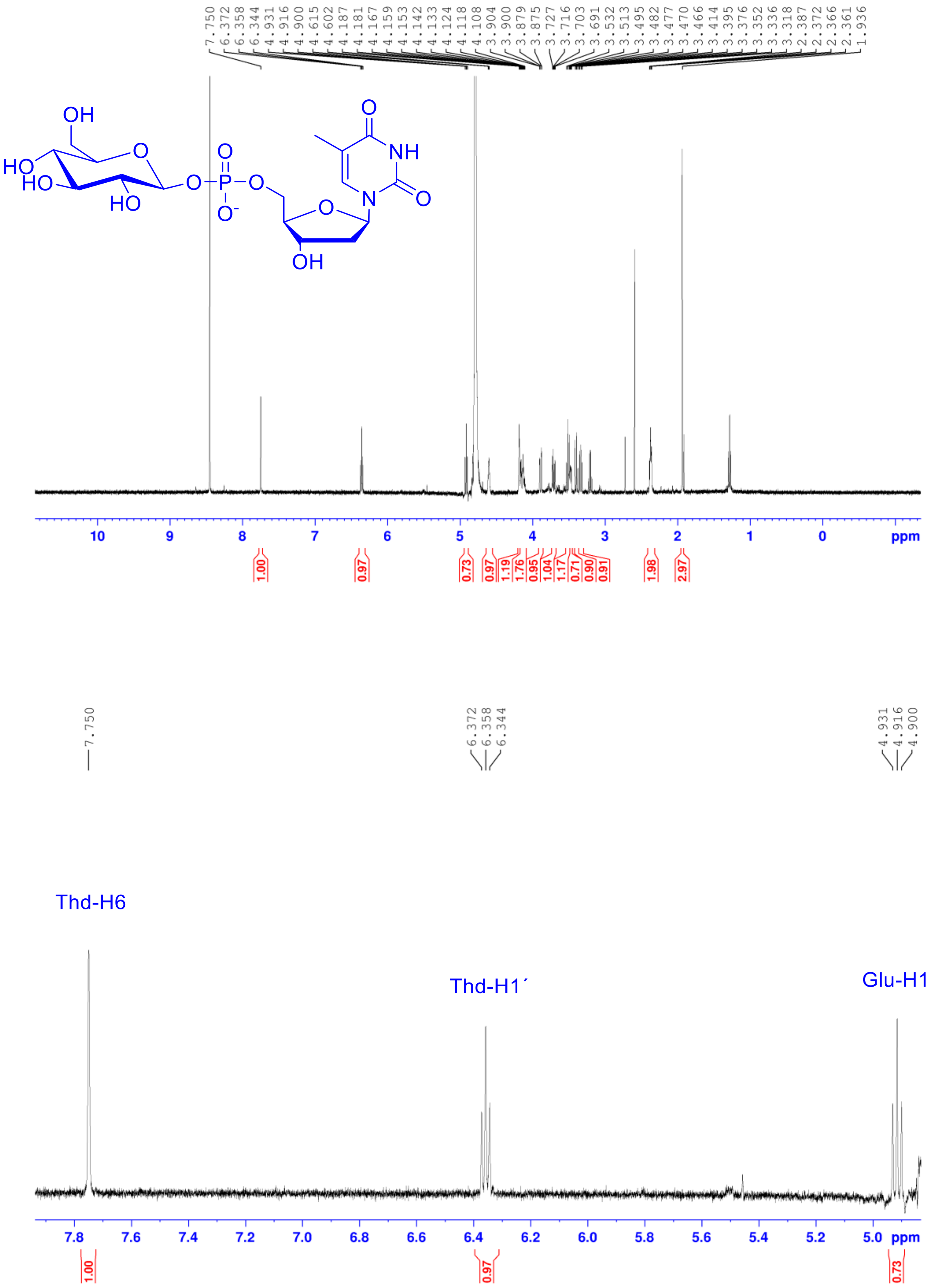

Figure S4. ^{1}H NMR spectrum (500 MHz, D_2O) of compound **6β**.

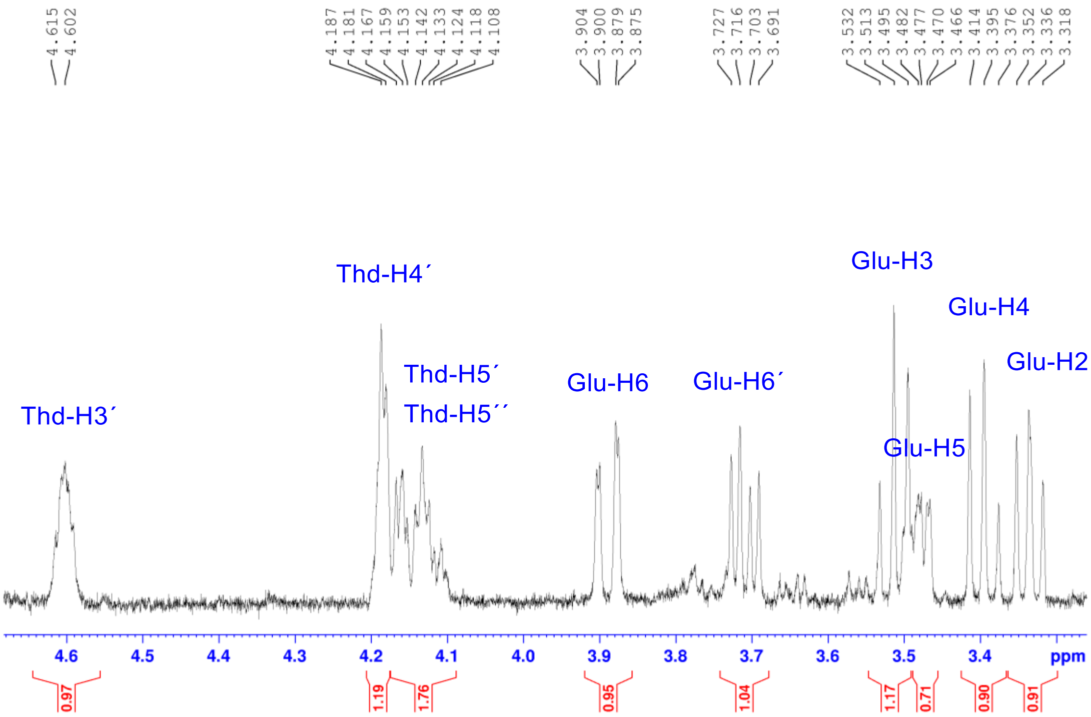

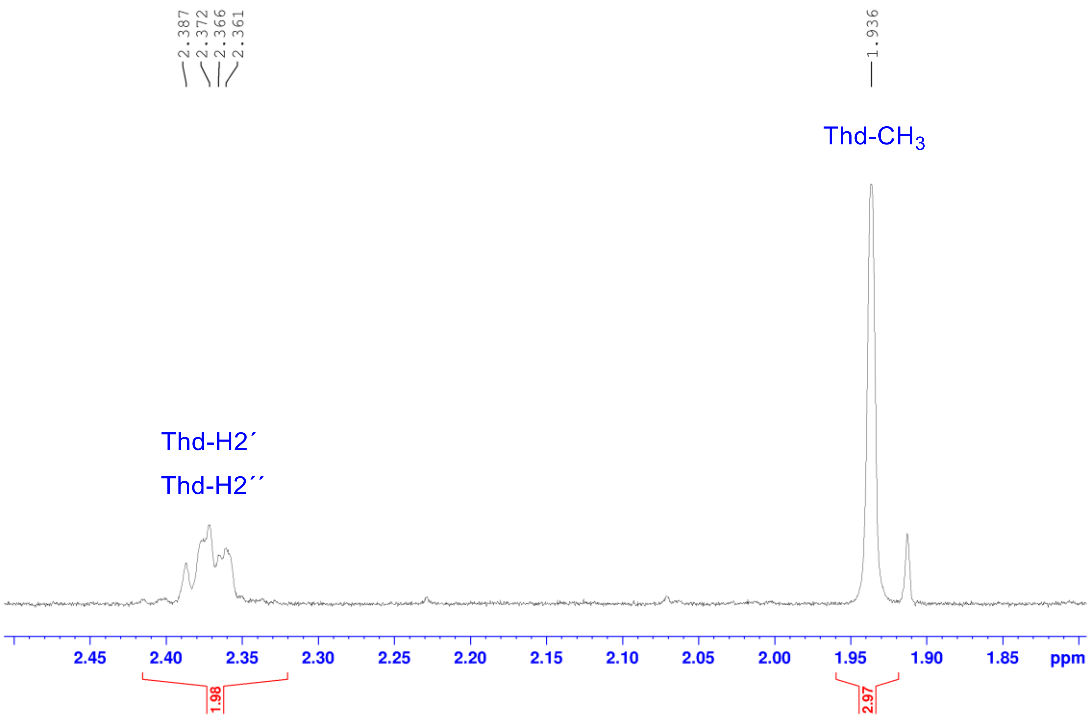

Figure S4 (continued). ^{1}H NMR spectrum (500 MHz, D_2O) of compound **6β**.

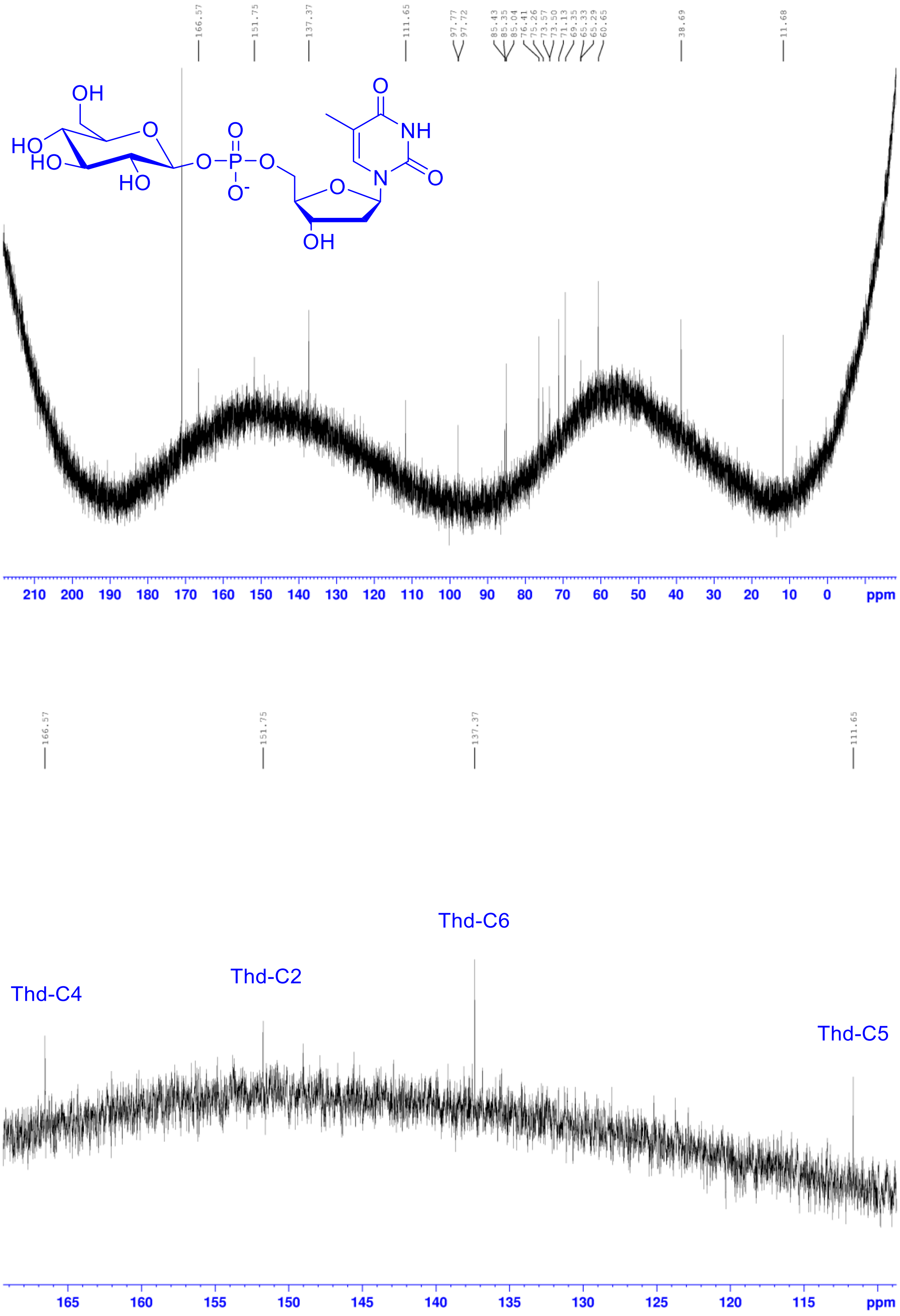

Figure S5. ^{13}C NMR spectrum (126 MHz, D_2O) of compound **6β**.

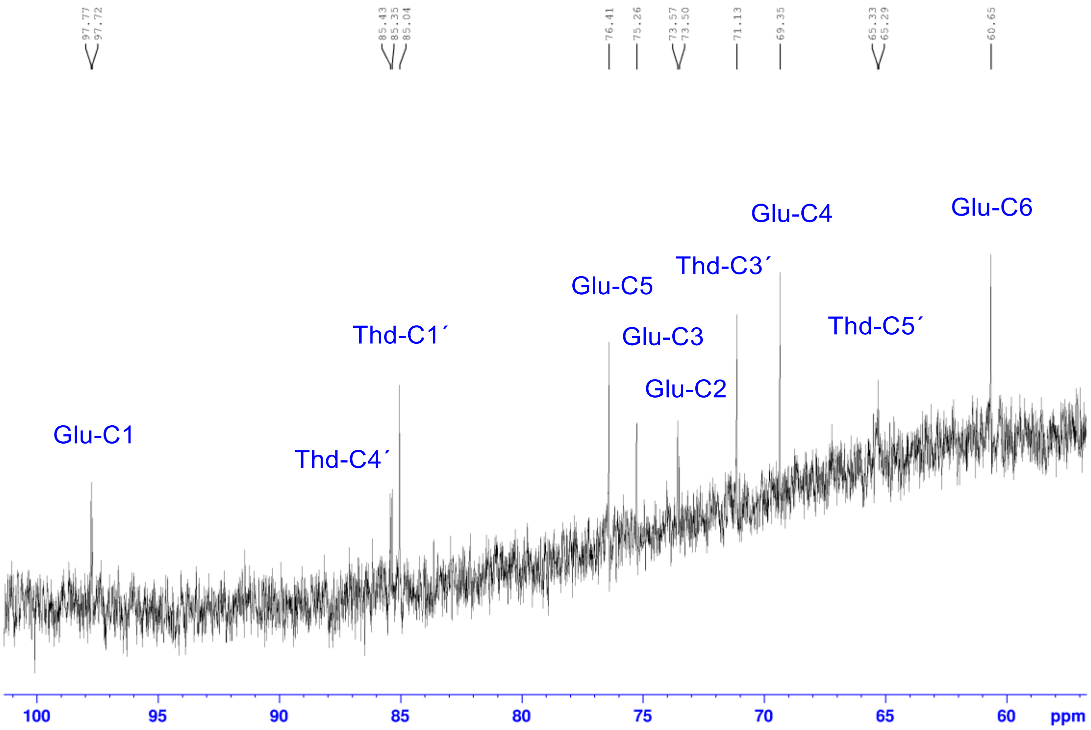

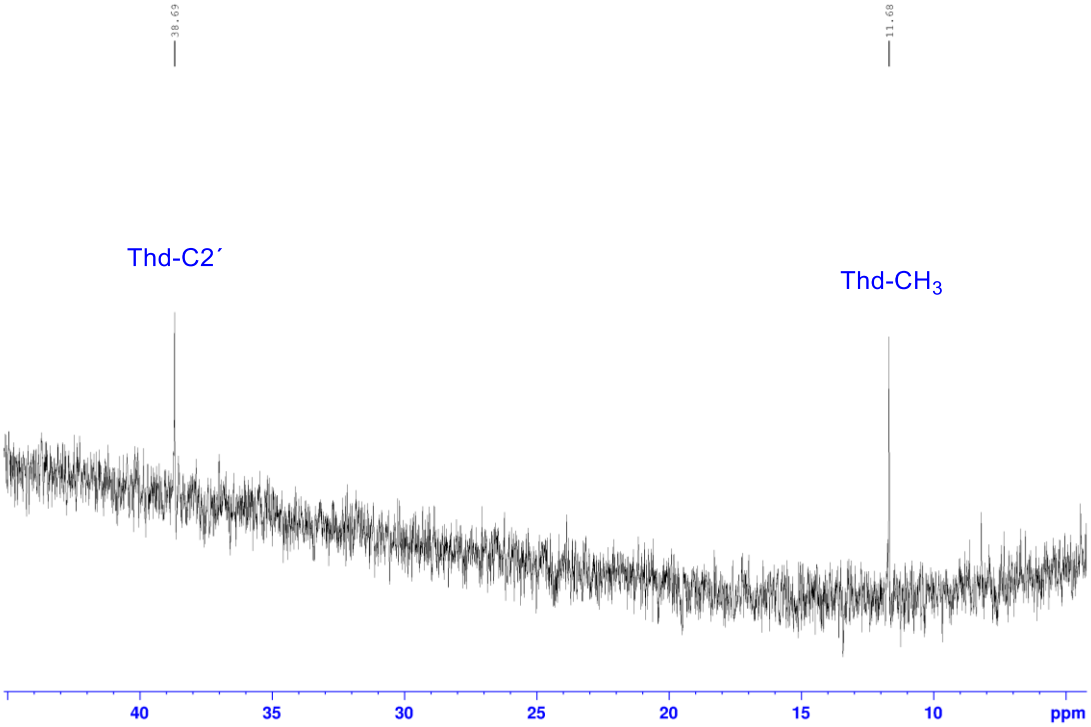

Figure S5 (continued). ^{13}C NMR spectrum (126 MHz, D_2O) of compound **6β**.

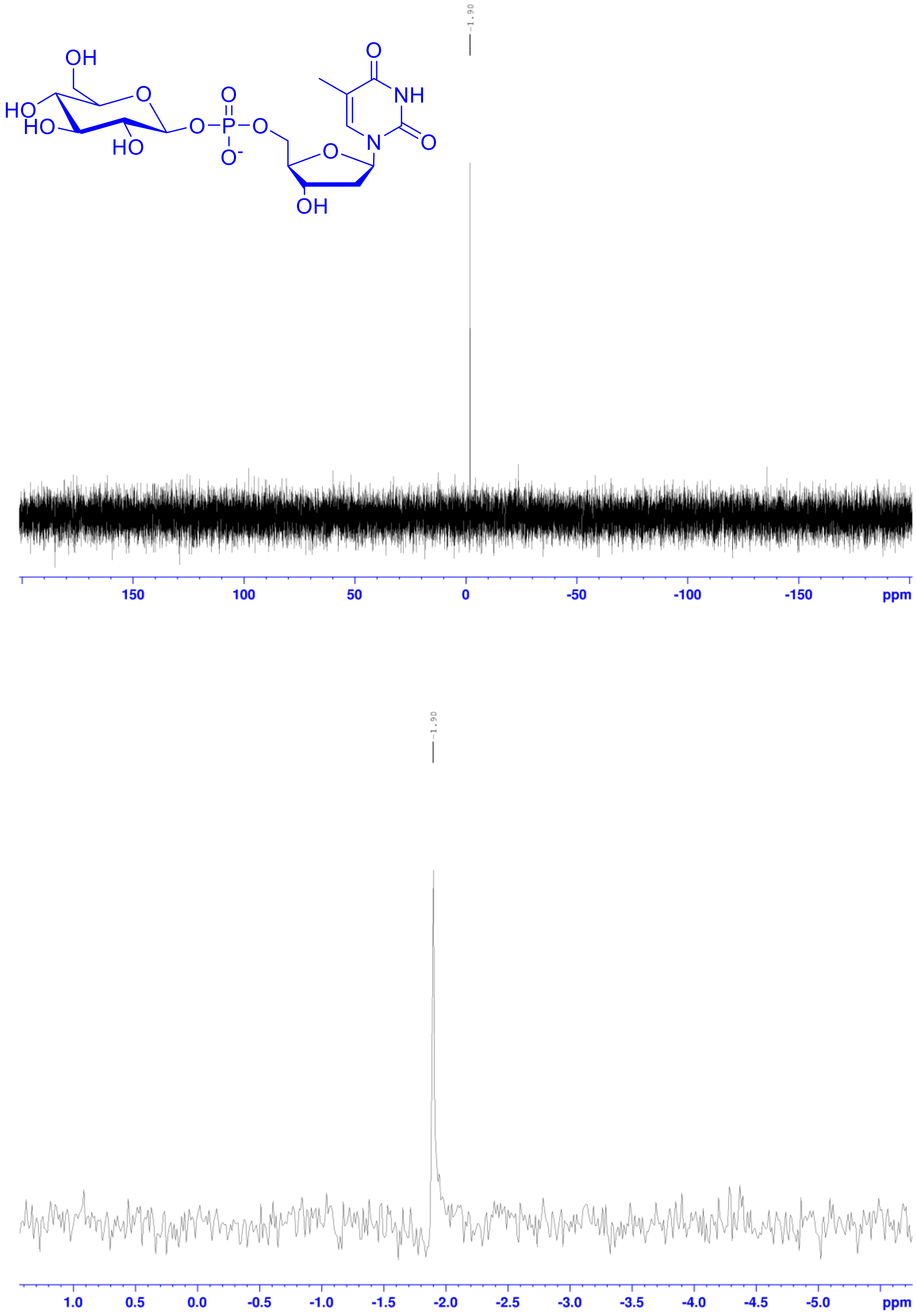

Figure S6. ^{31}P NMR spectrum (202 MHz, D_2O) of compound **6β**.

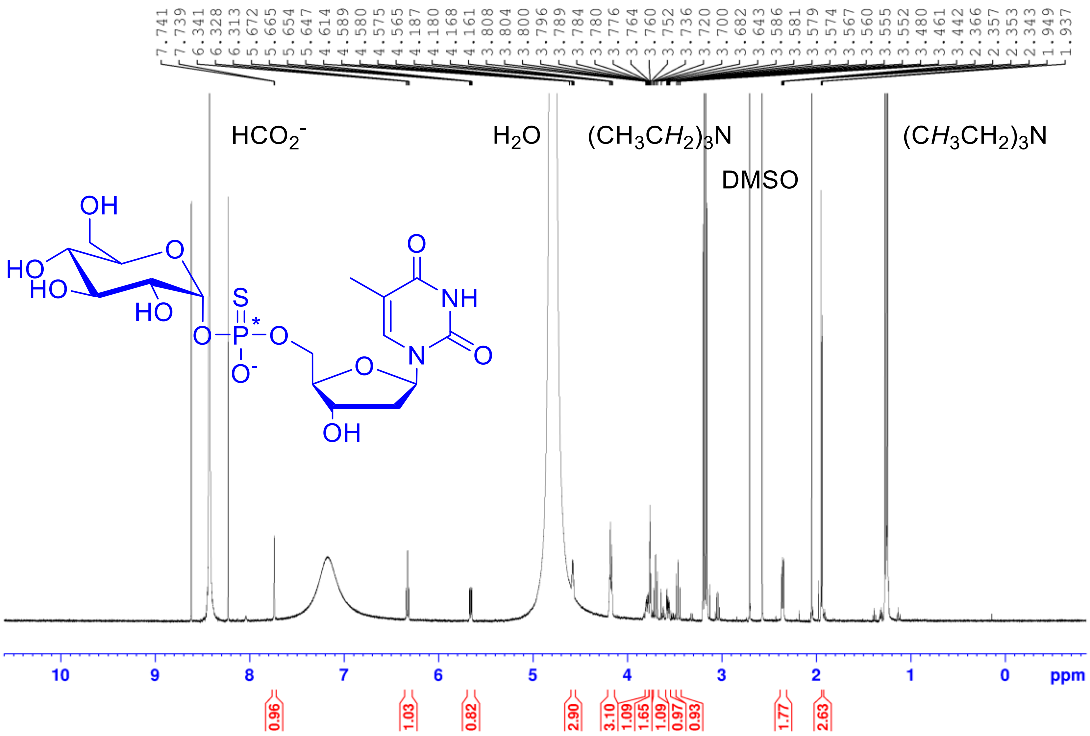

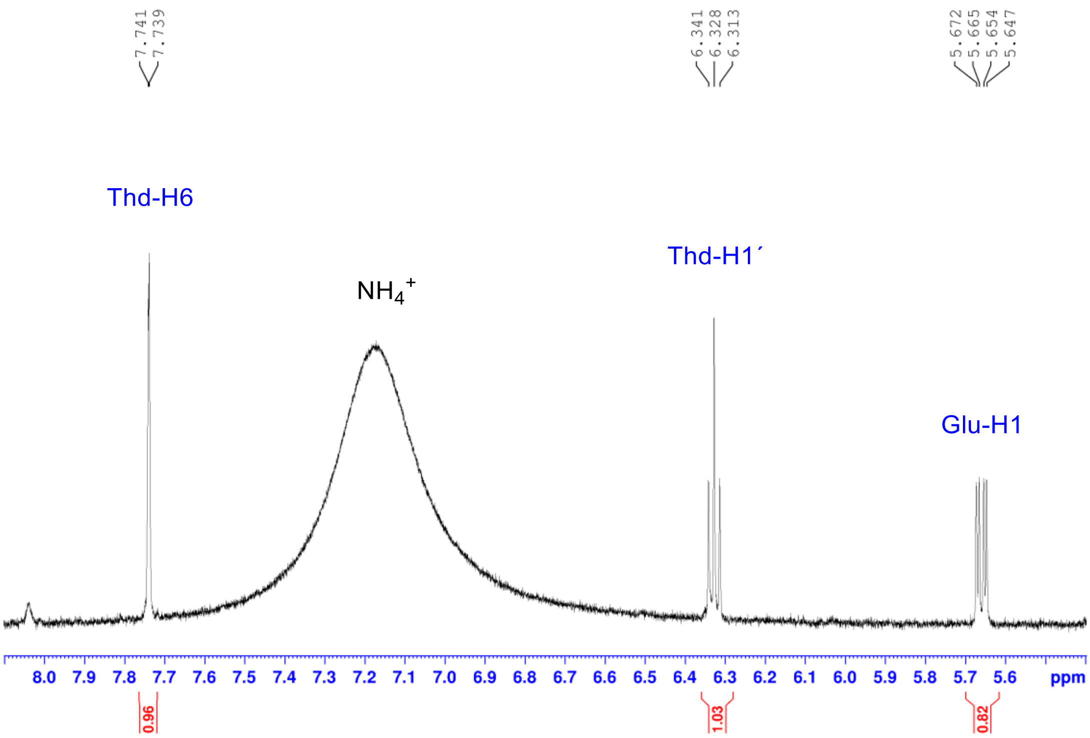

Figure S7. ^{1}H NMR spectrum (500 MHz, D_2O) of compound **7α1**.

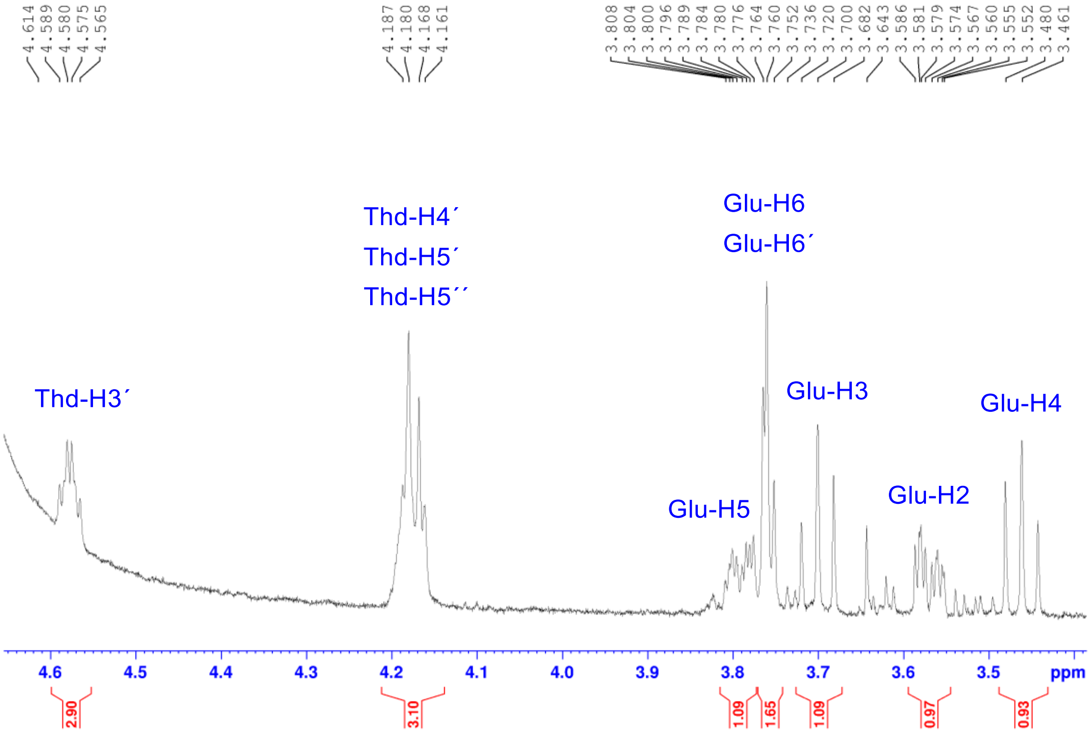

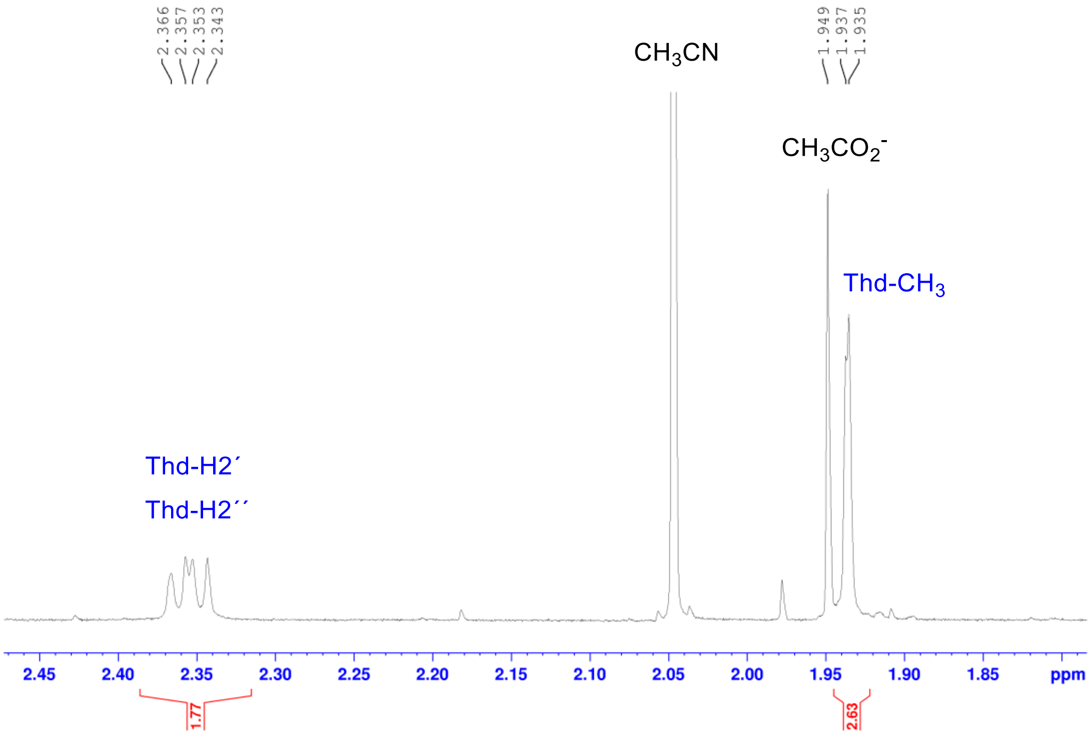

Figure S7 (continued). ^{1}H NMR spectrum (500 MHz, D_2O) of compound **7α1**.

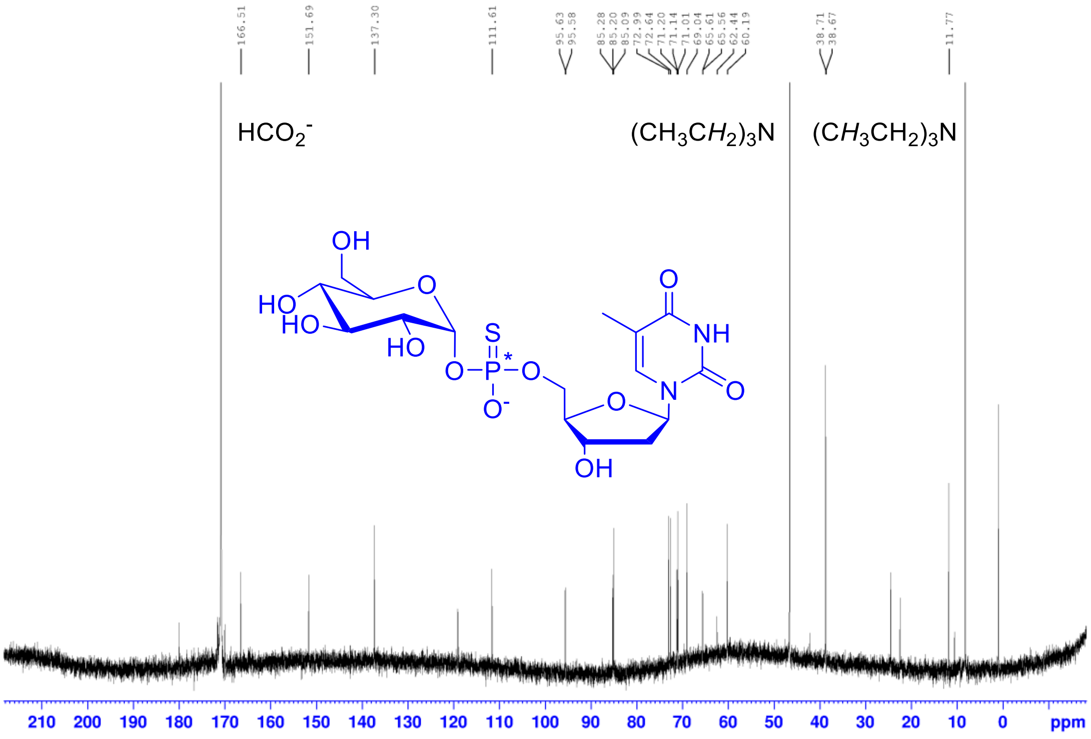

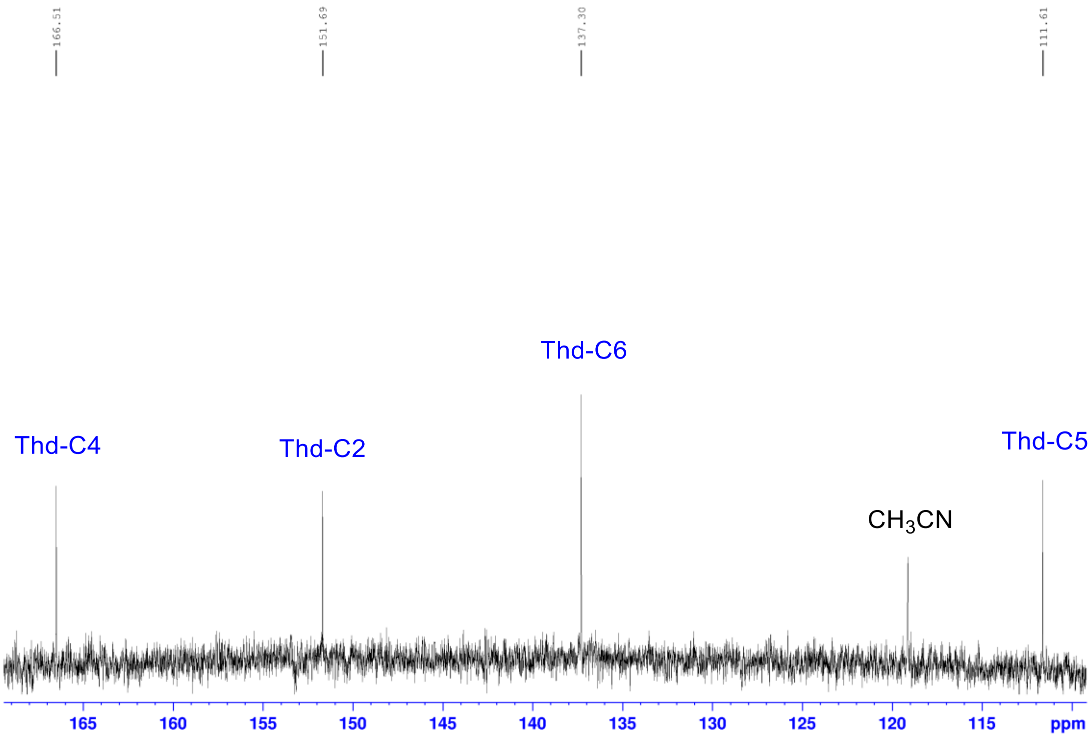

Figure S8. ^{13}C NMR spectrum (126 MHz, D_2O) of compound **7α1**.

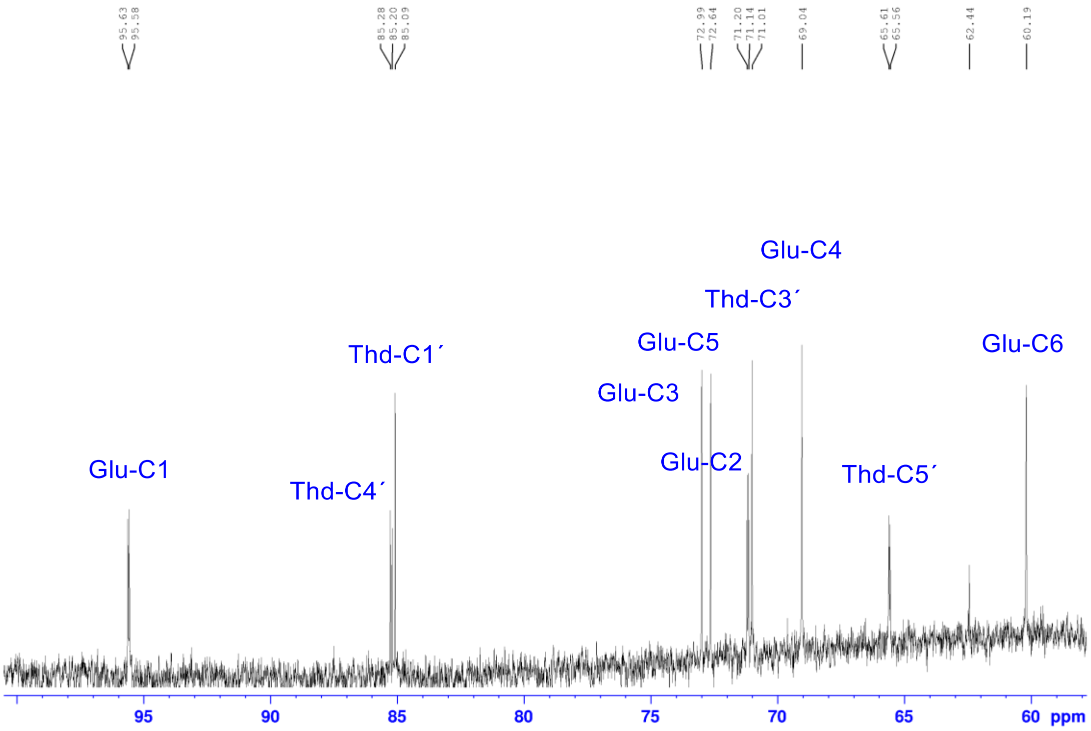

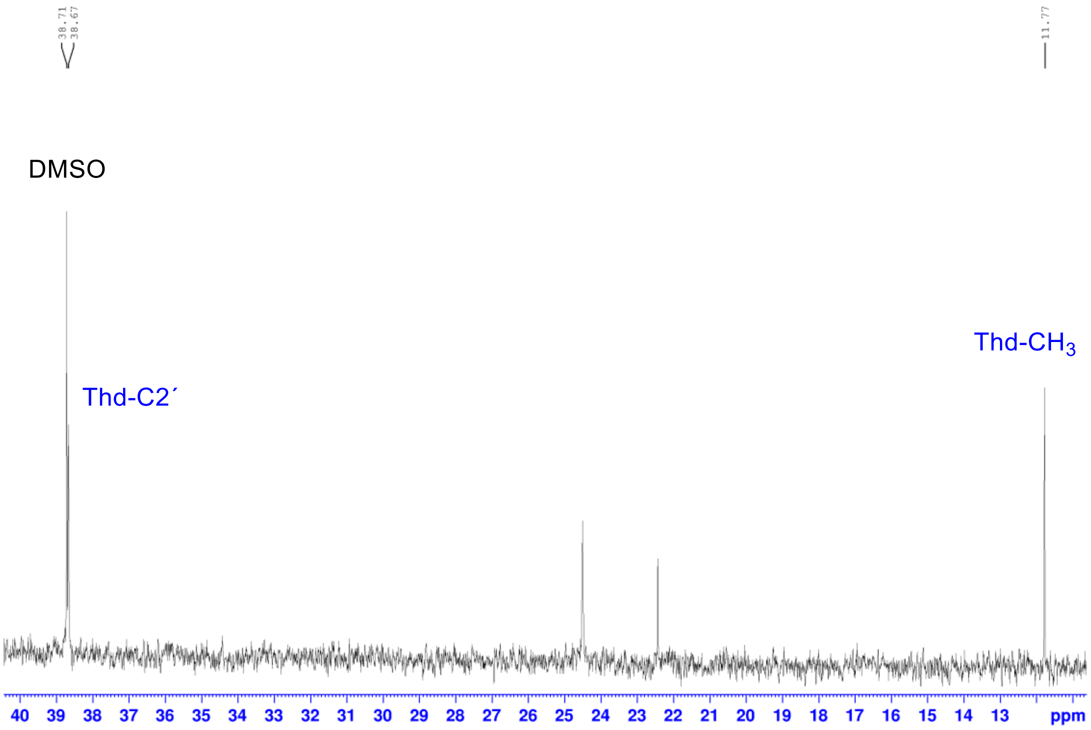

Figure S8 (continued). ^{13}C NMR spectrum (126 MHz, D_2O) of compound **7α1**.

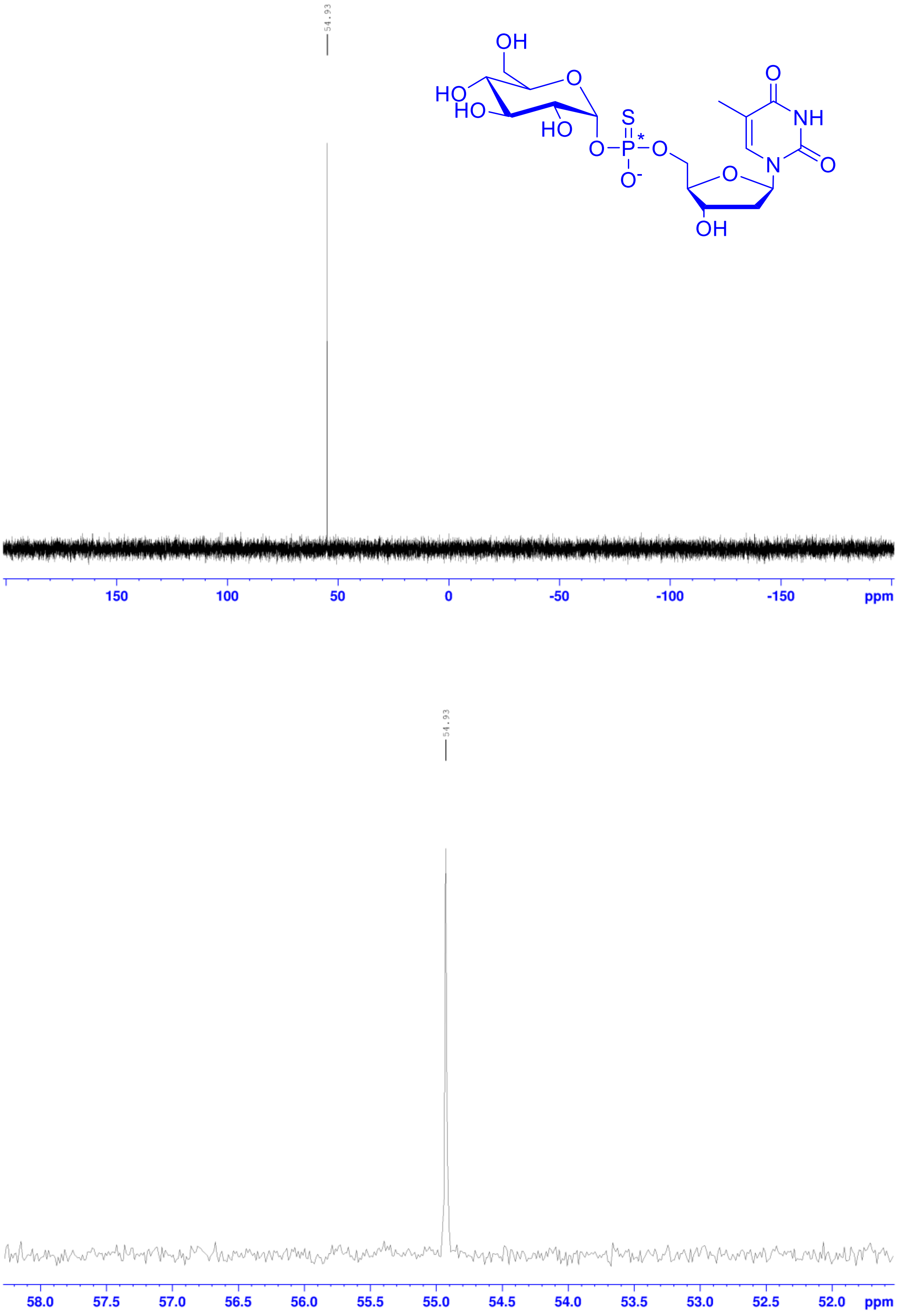

Figure S9. ^{31}P NMR spectrum (202 MHz, D_2O) of compound **7α1**.

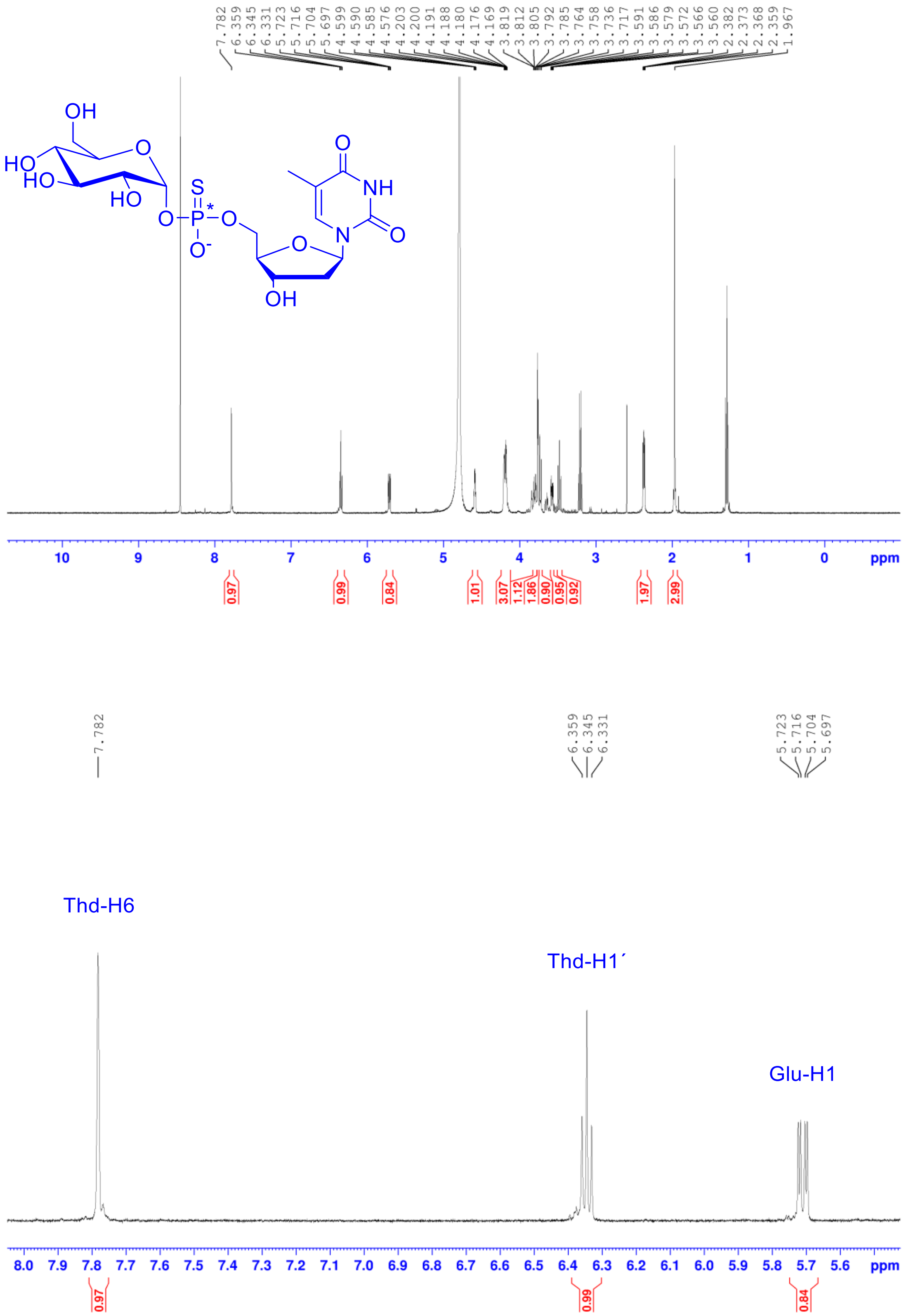

Figure S10. ^{1}H NMR spectrum (500 MHz, D_2O) of compound **7α2**.

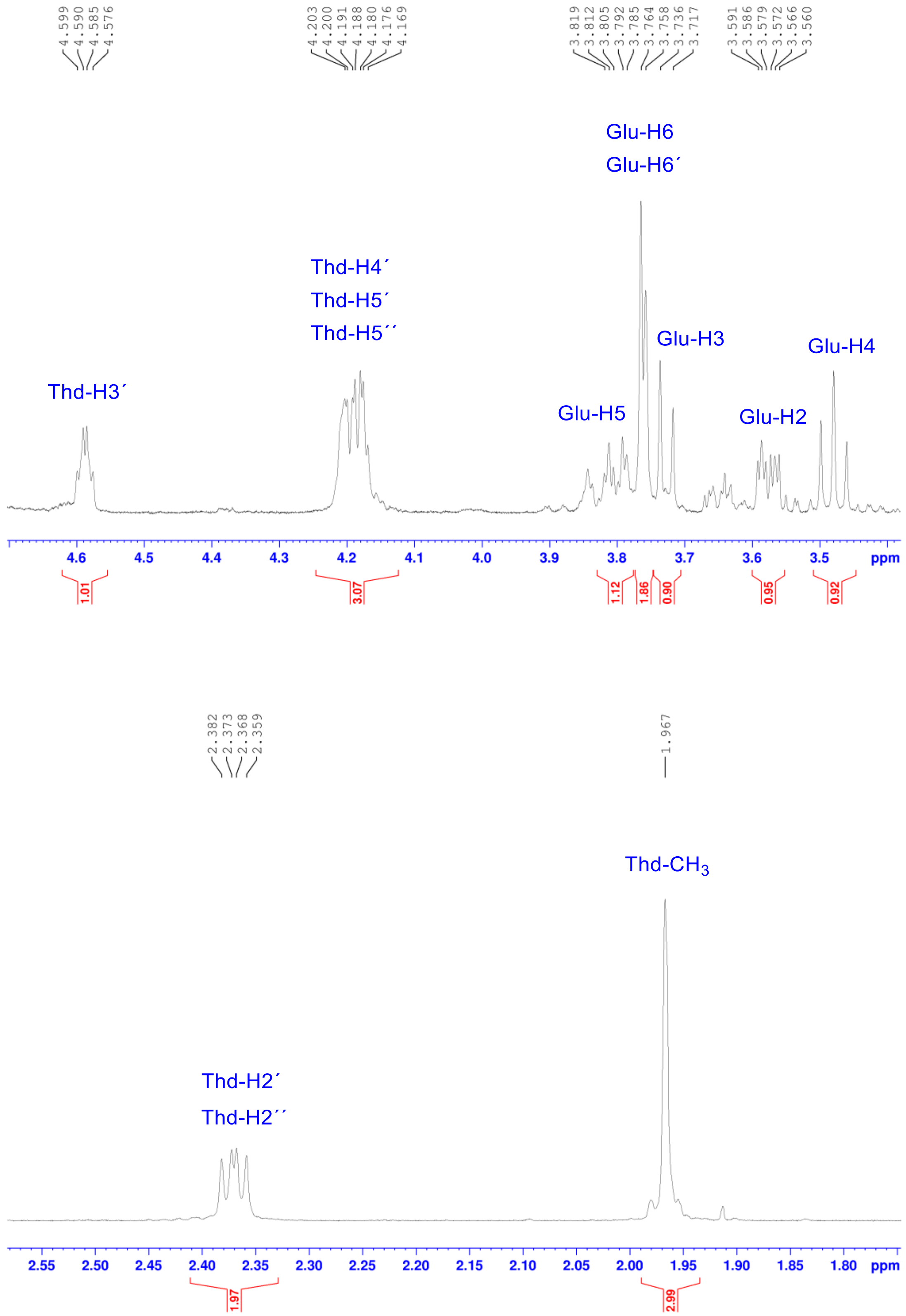

Figure S10 (continued). ^{1}H NMR spectrum (500 MHz, D_2O) of compound **7α2**.

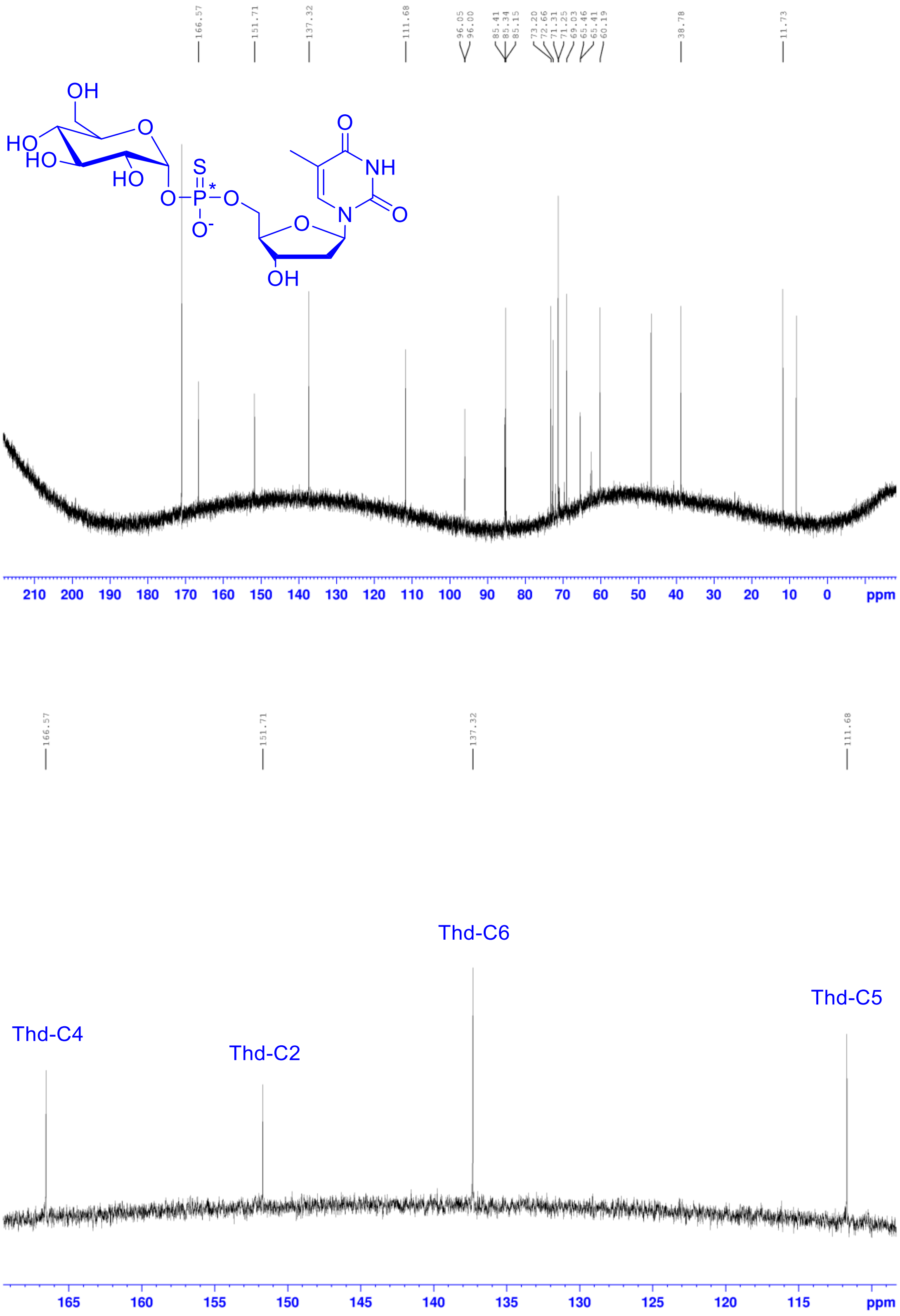

Figure S11. ^{13}C NMR spectrum (126 MHz, D_2O) of compound **7α2**.

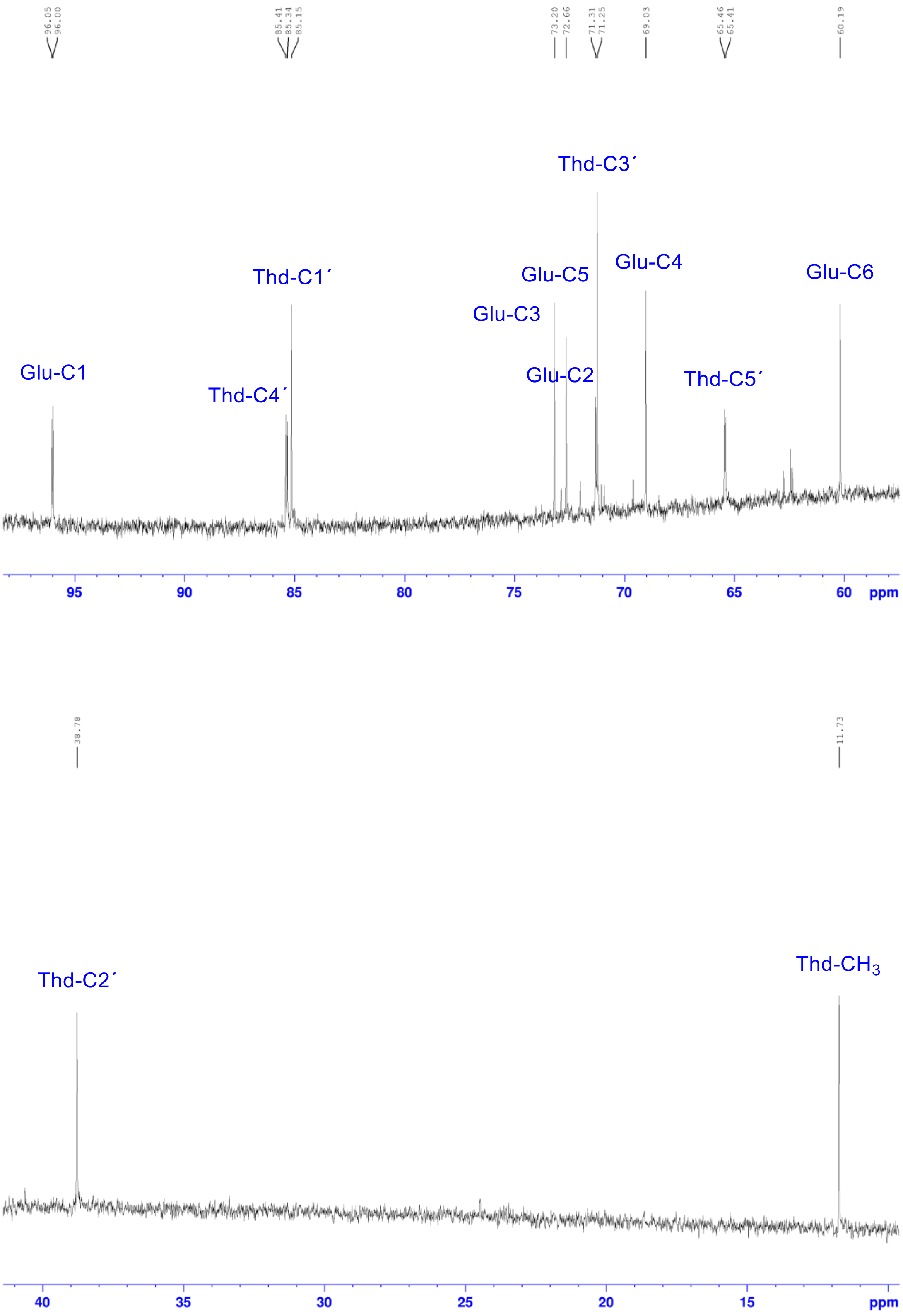

Figure S11 (continued). ^{13}C NMR spectrum (126 MHz, D_2O) of compound **7α2**.

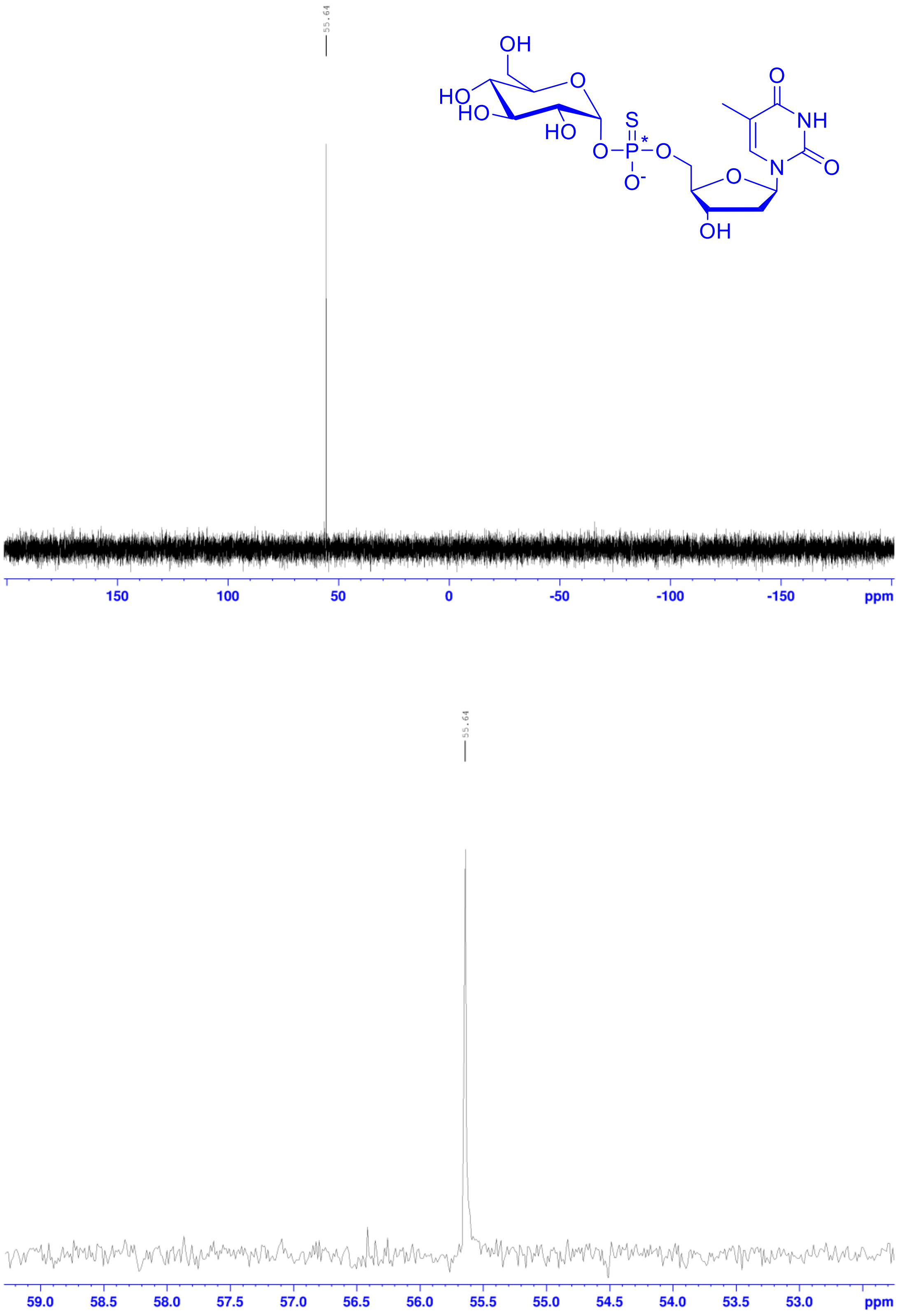

Figure S12. ^{31}P NMR spectrum (202 MHz, D_2O) of compound **7α2**.

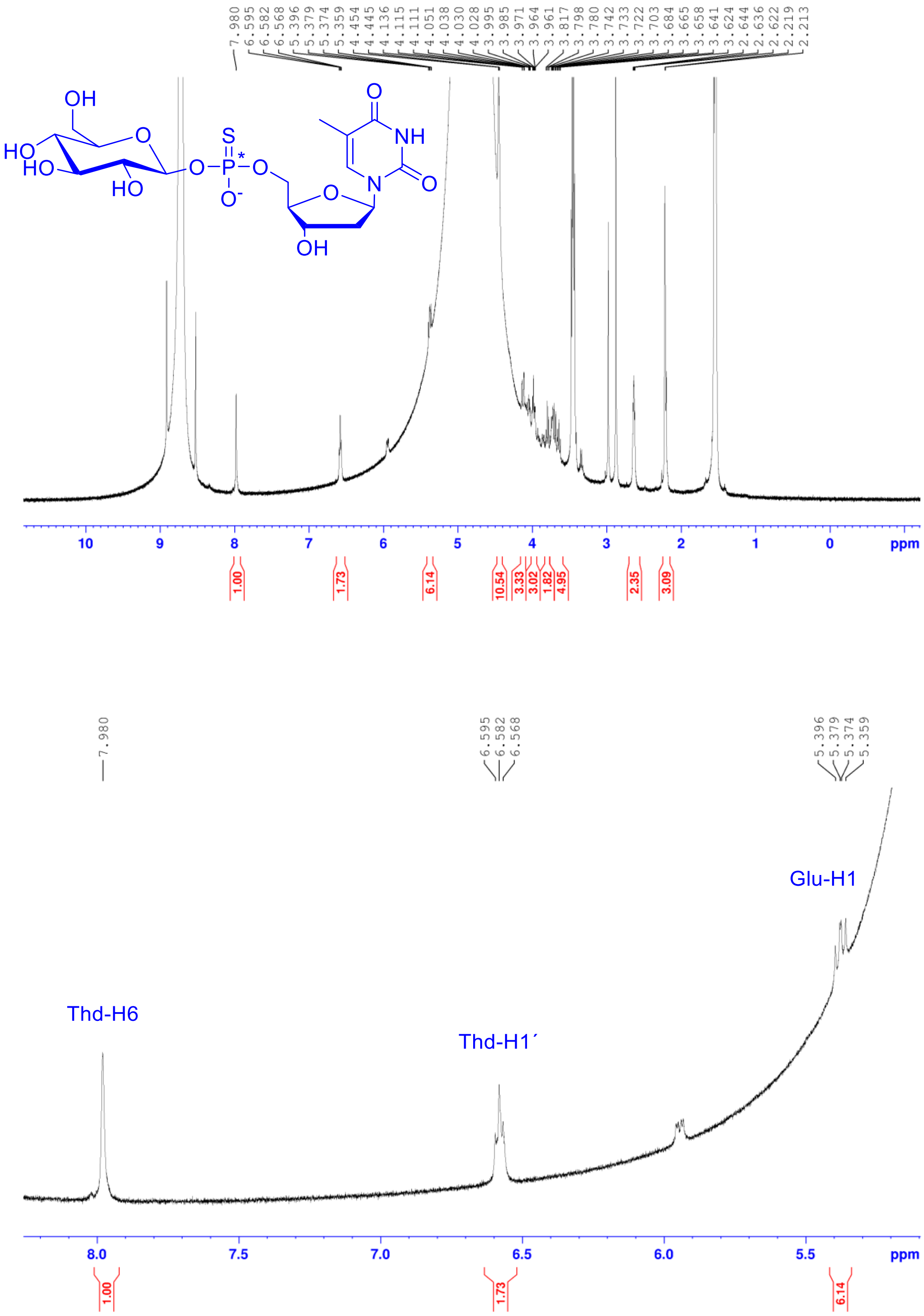

Figure S13. ^{1}H NMR spectrum (500 MHz, D_2O) of compound **7β1**.

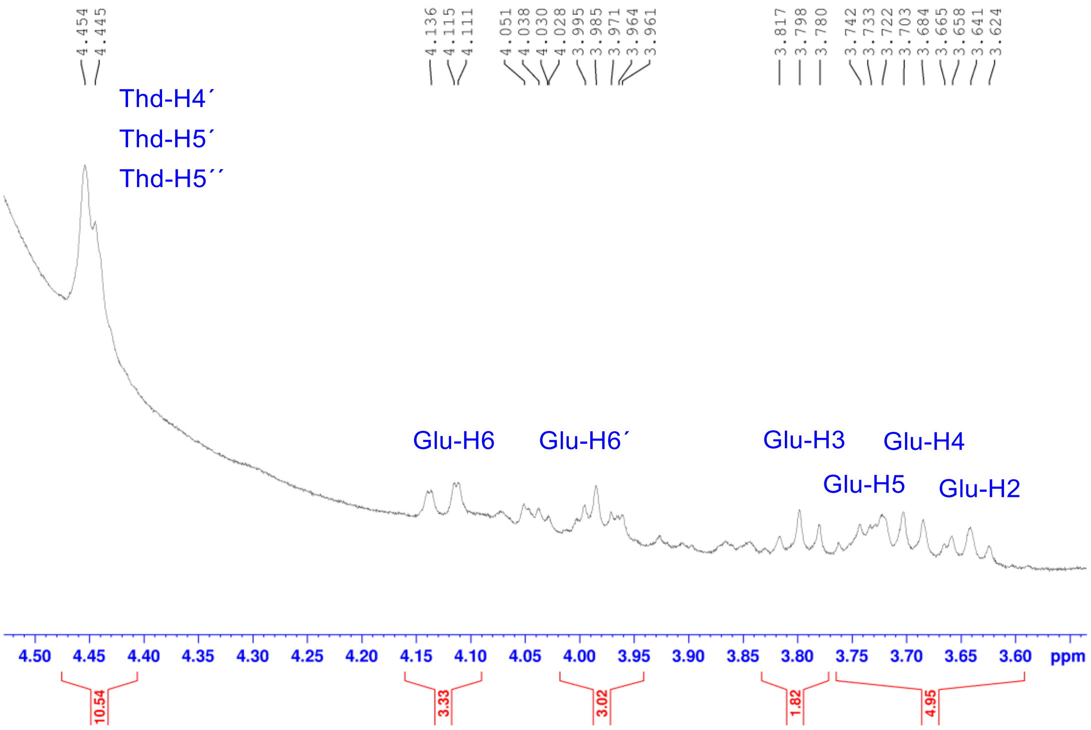

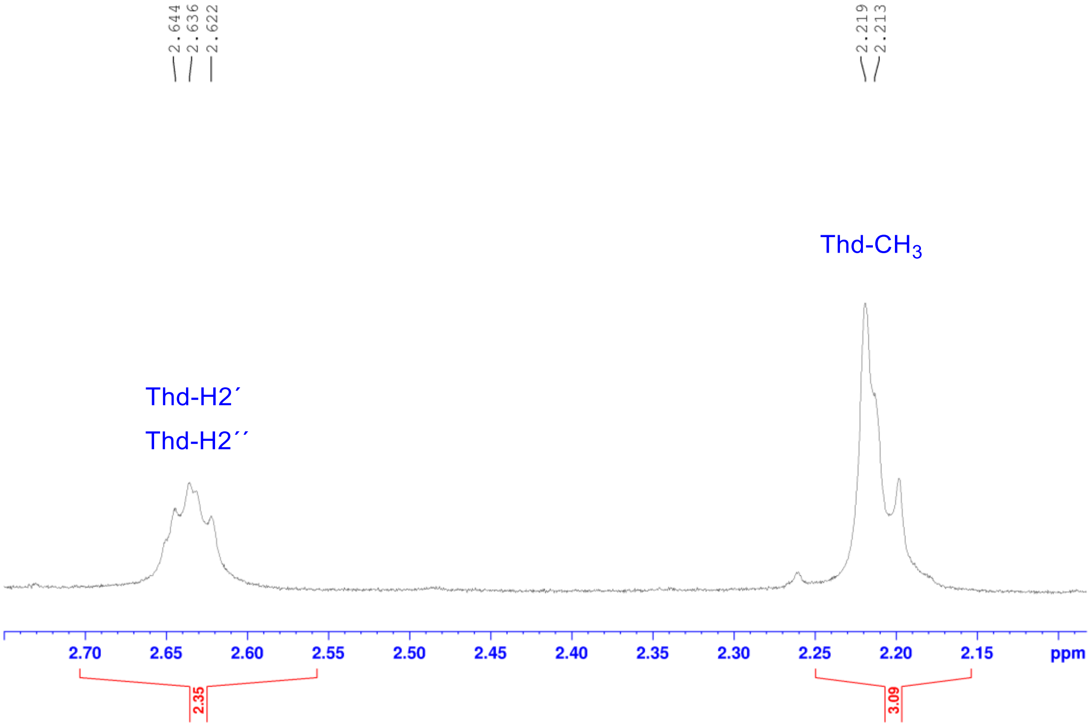

Figure S13 (continued). ^{1}H NMR spectrum (500 MHz, D_2O) of compound **7β1**.

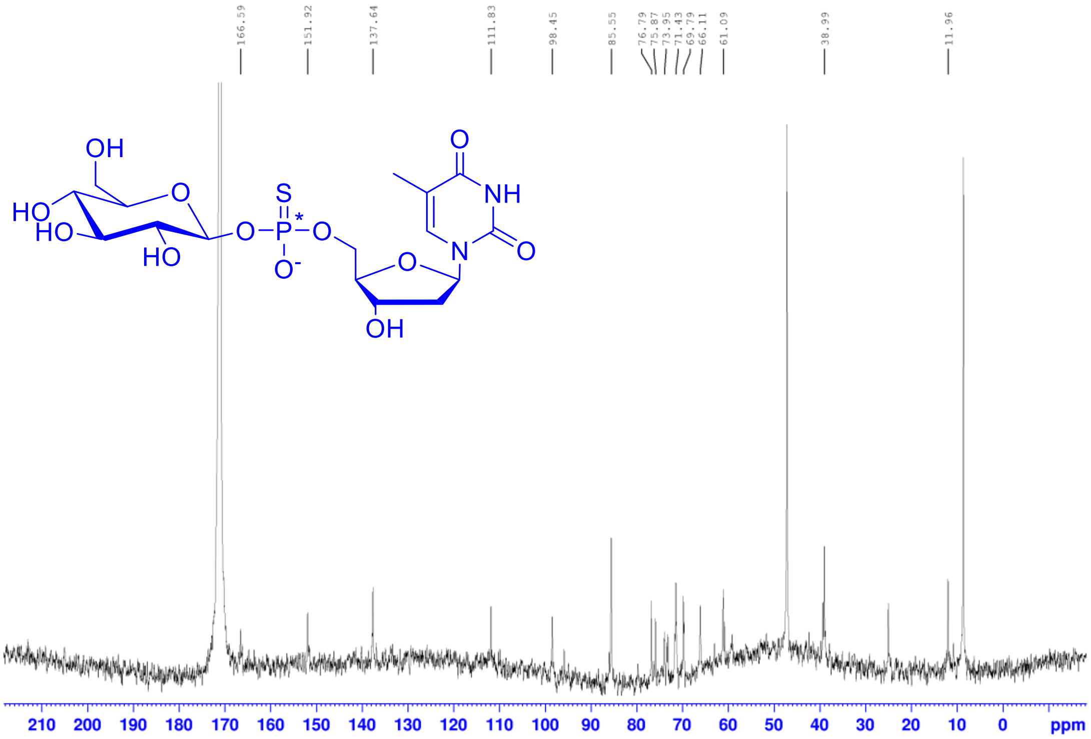

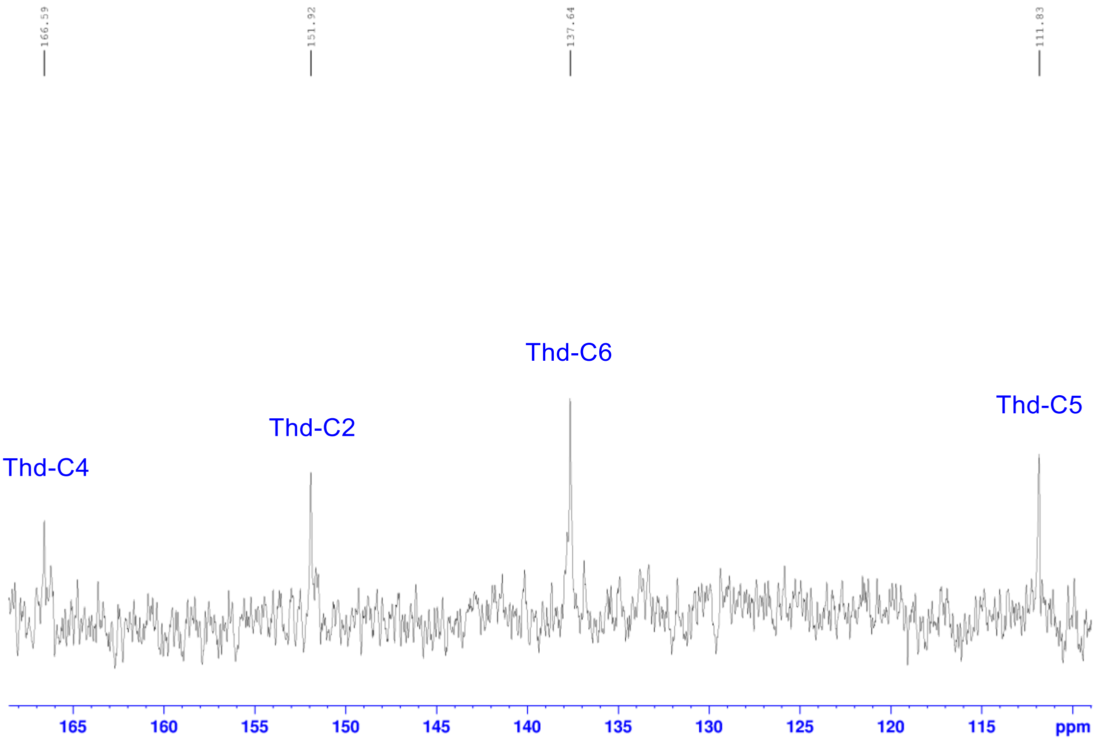

Figure S14. ^{13}C NMR spectrum (126 MHz, D_2O) of compound **7β1**.

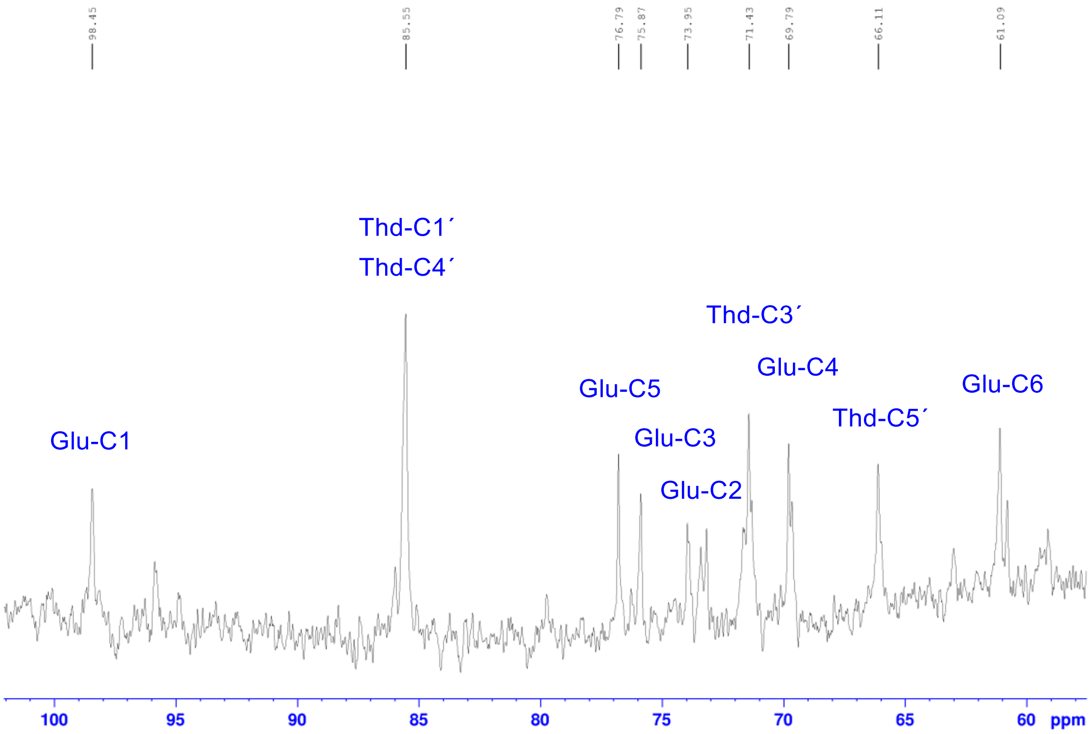

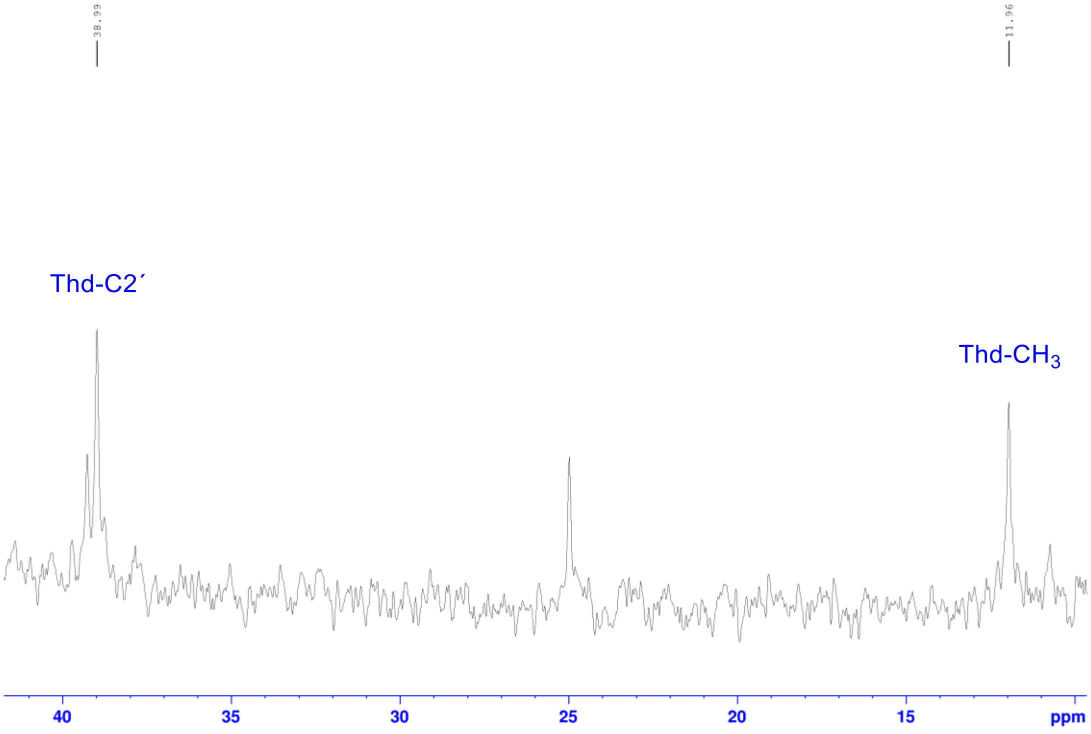

Figure S14 (continued). ^{13}C NMR spectrum (126 MHz, D_2O) of compound **7β1**.

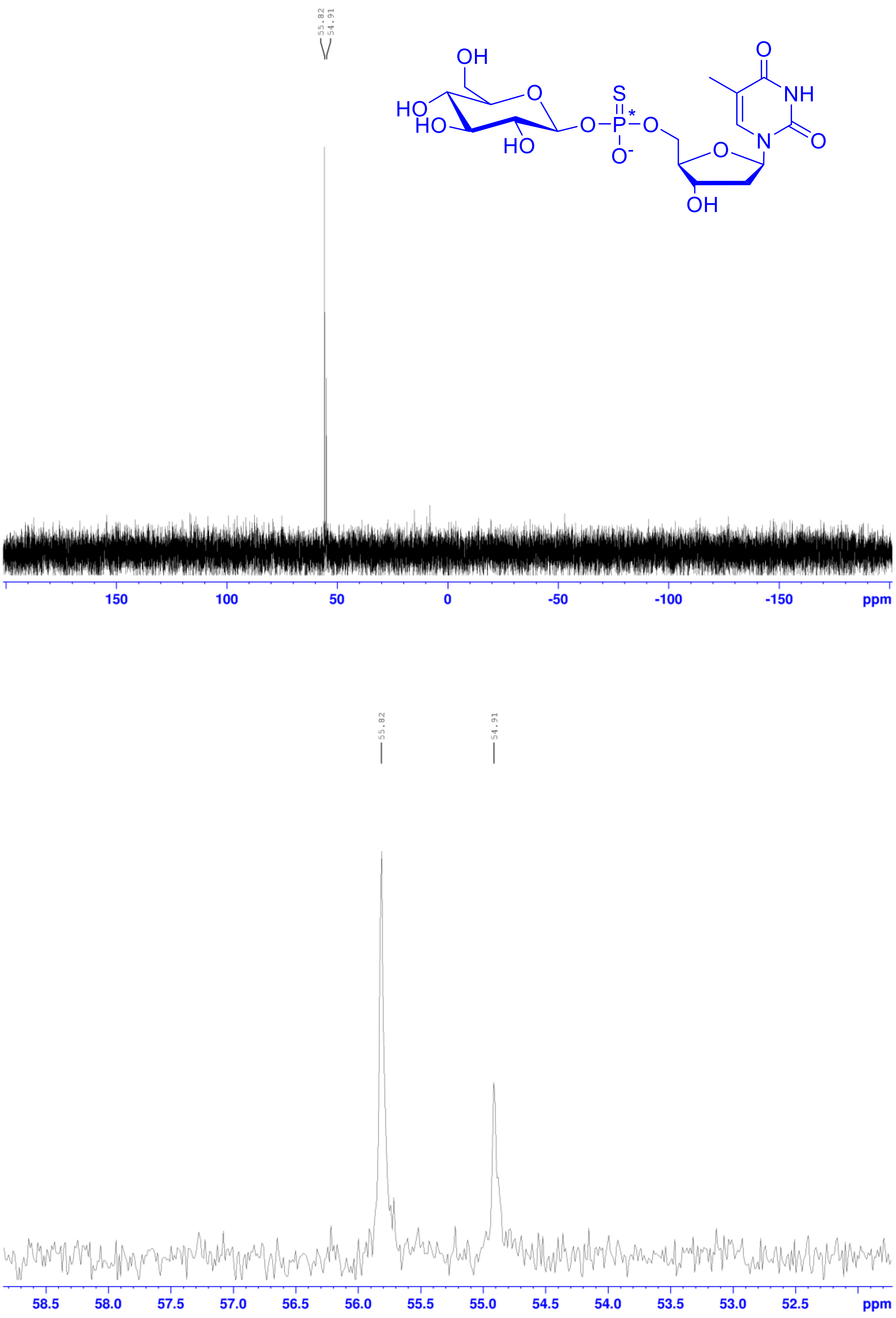

Figure S15. ^{31}P NMR spectrum (202 MHz, D_2O) of compound **7β1**.

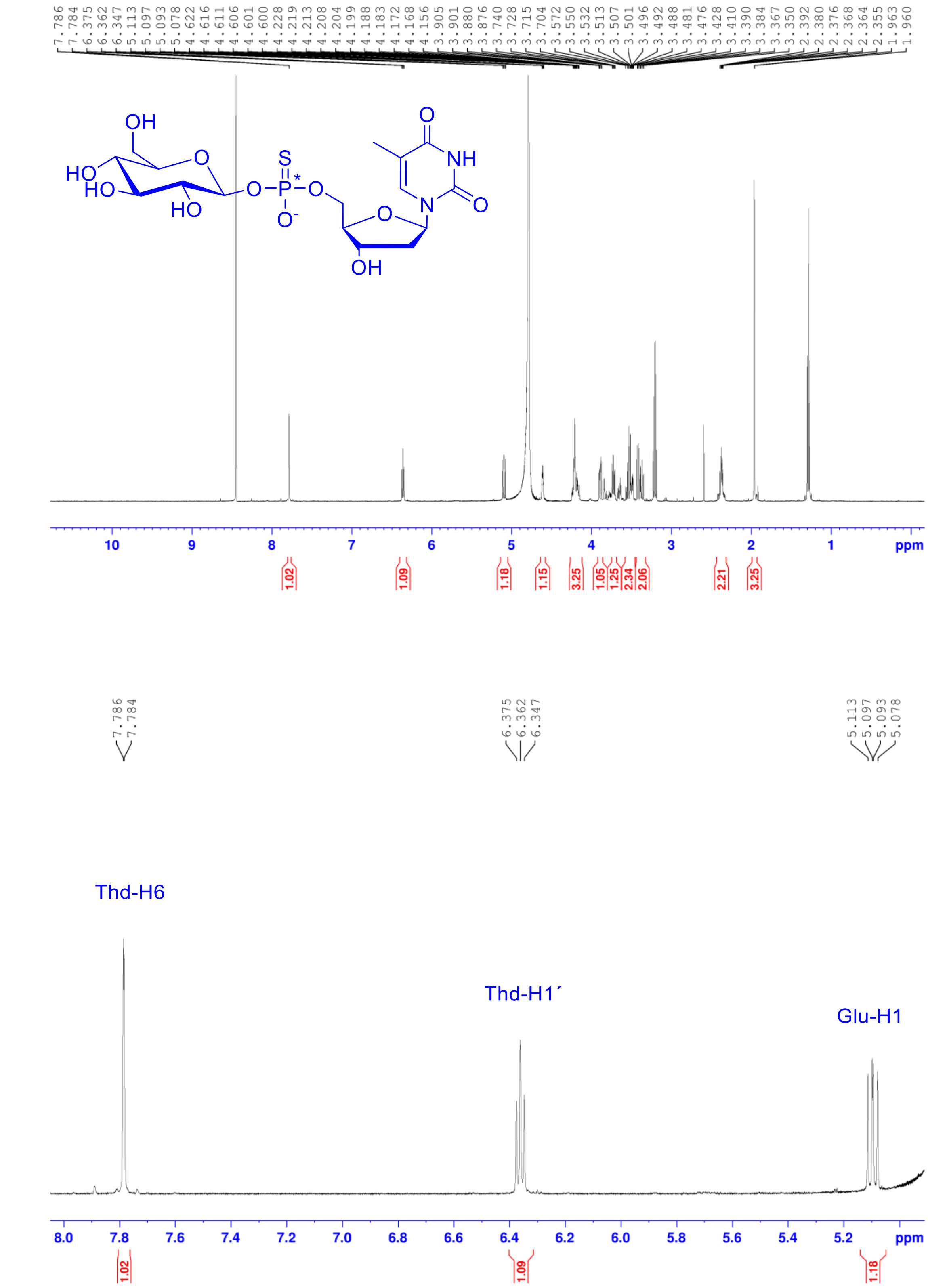

Figure S16. ^{1}H NMR spectrum (500 MHz, D_2O) of compound **7β2**.

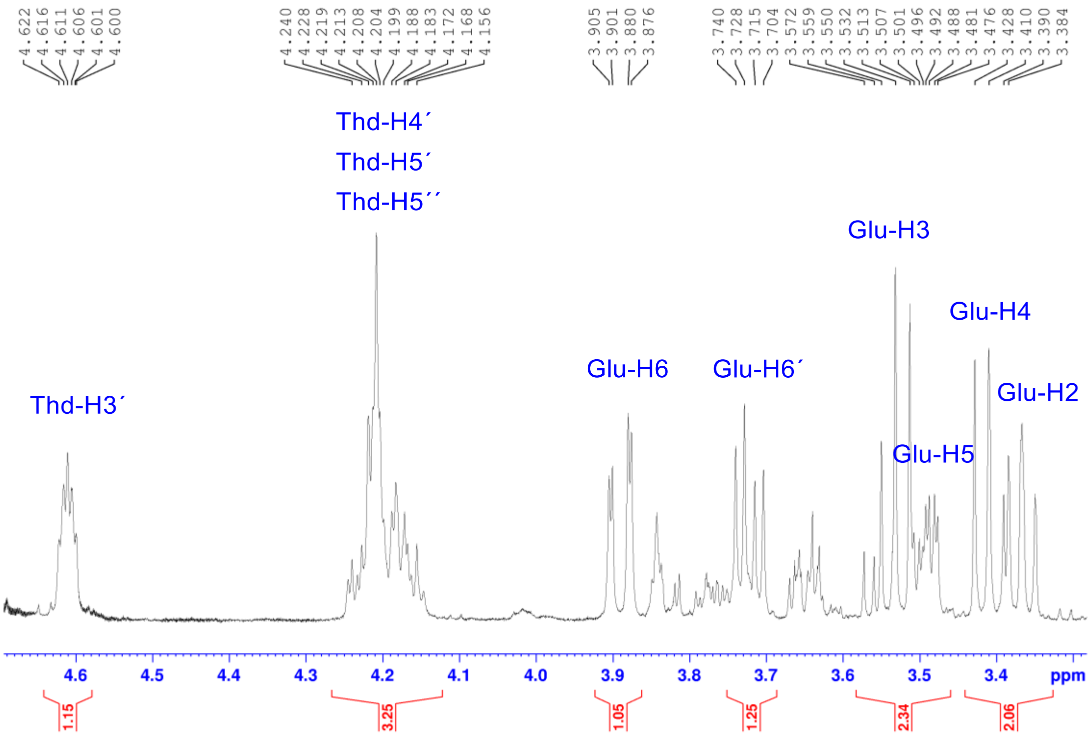

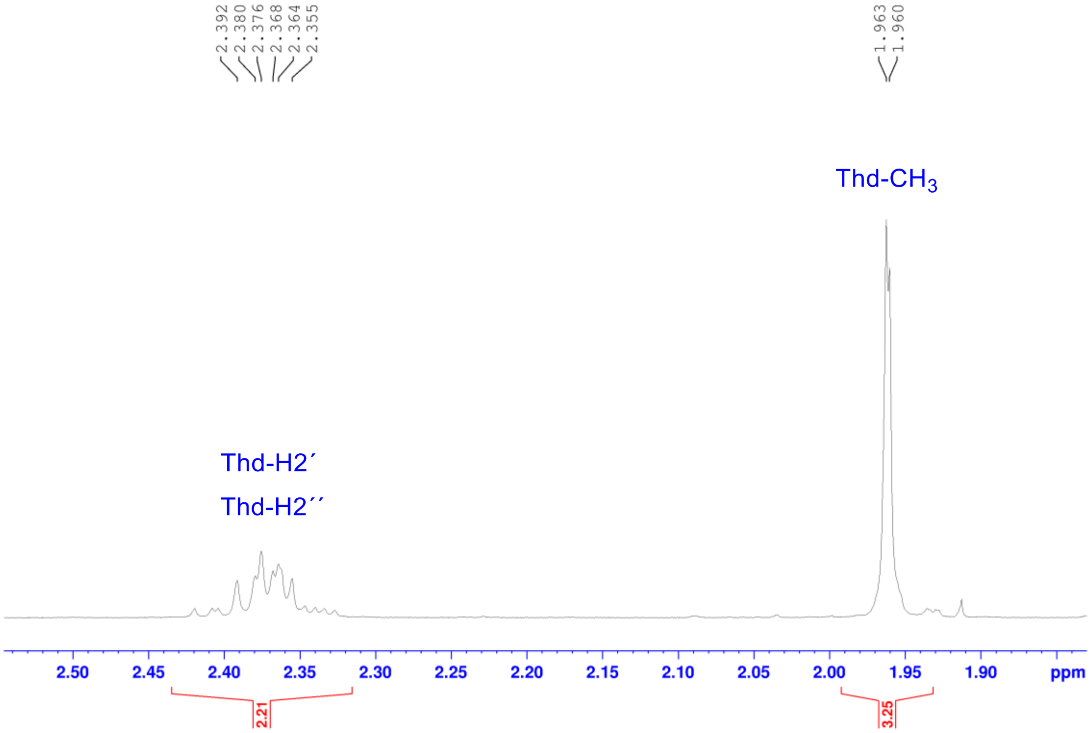

Figure S16 (continued). ^{1}H NMR spectrum (500 MHz, D_2O) of compound **7β2**.

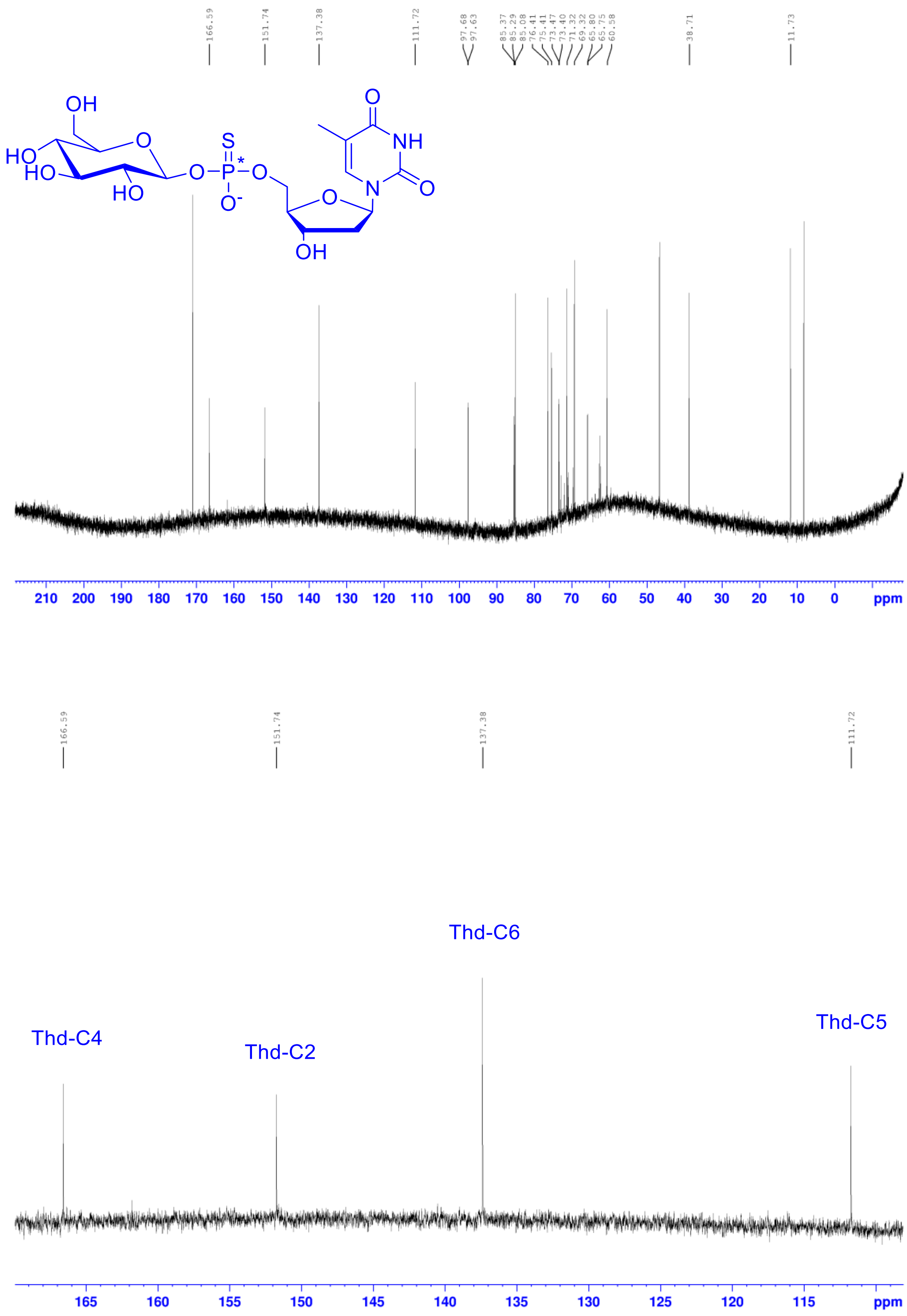

Figure S17. ^{13}C NMR spectrum (126 MHz, D_2O) of compound **7β2**.

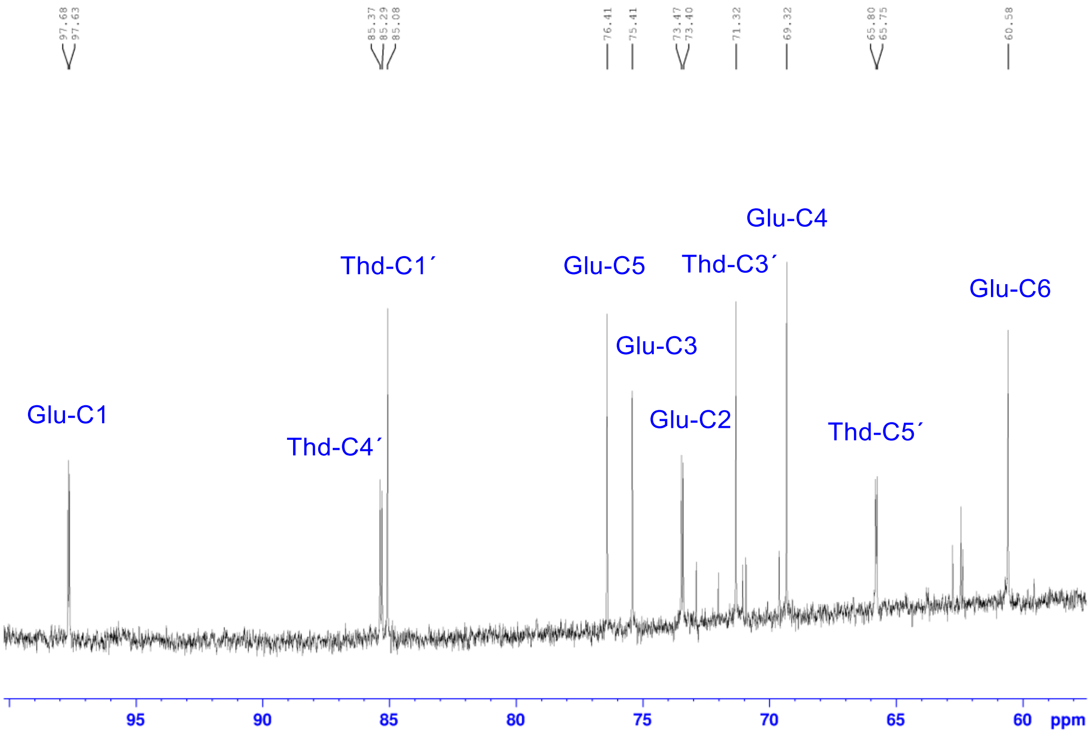

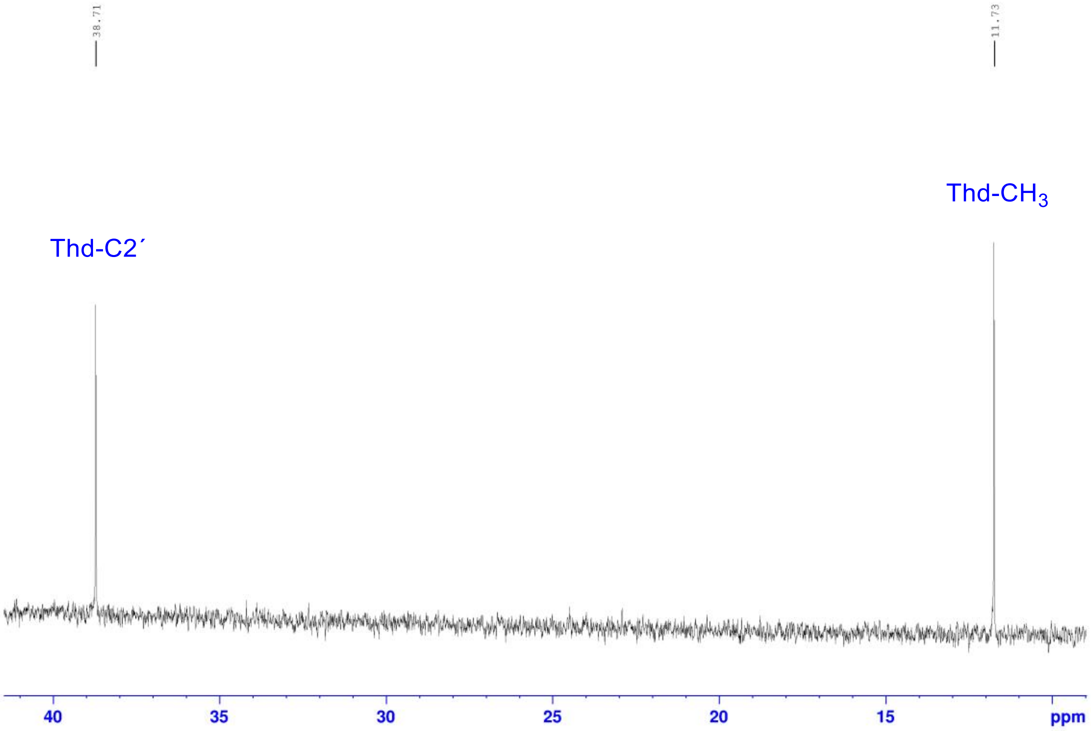

Figure S17 (continued). ^{13}C NMR spectrum (126 MHz, D_2O) of compound **7β2**.

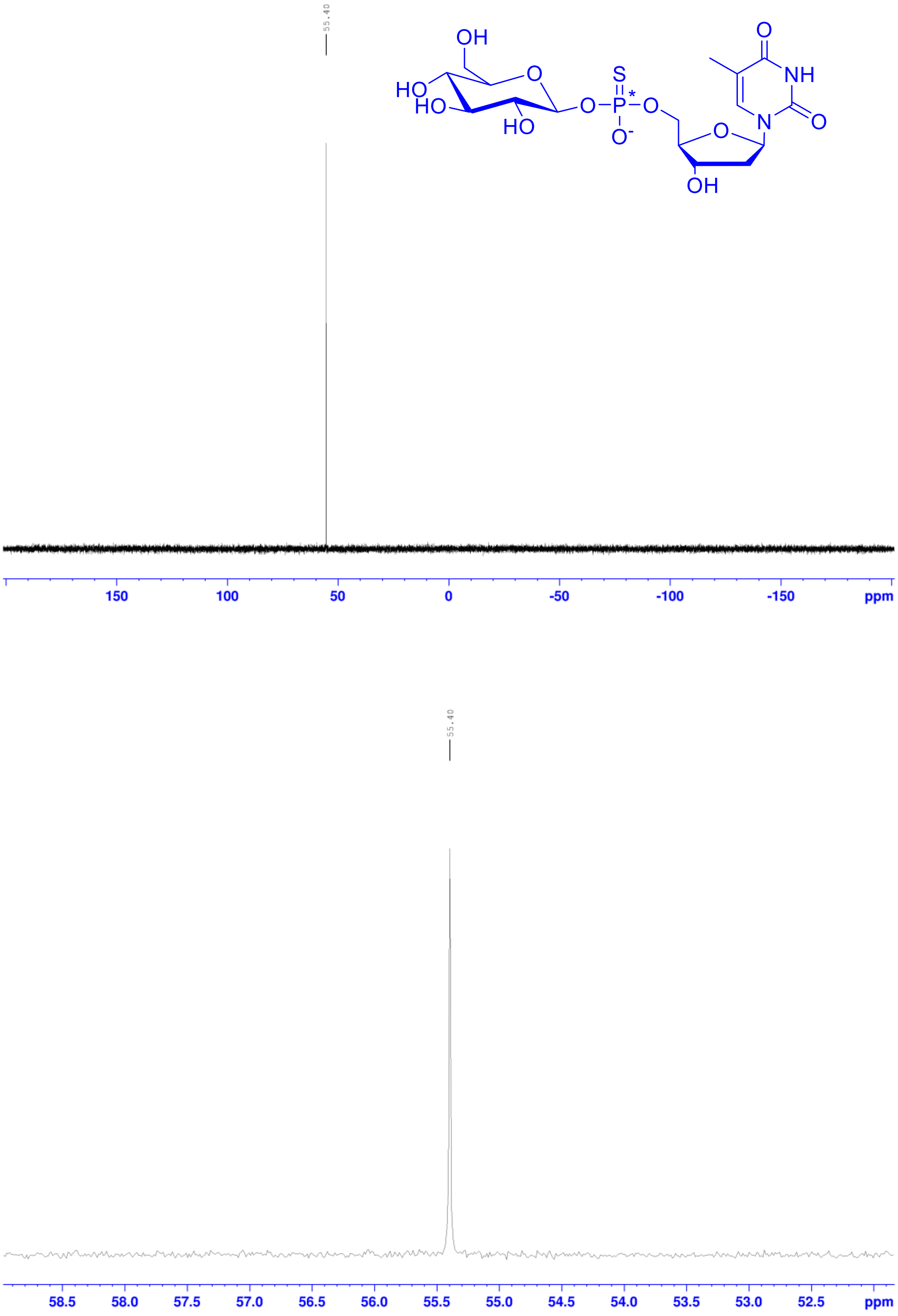

Figure S18. ^{31}P NMR spectrum (202 MHz, D_2O) of compound **7β2**.

Conditions of capillary electrophoresis and HPLC analysis

Electropherograms and chromatograms have been obtained under the following experimental conditions, unless otherwise stated.

Electropherograms: fused silica capillary (77 cm effective length, *i.d*. 75 µm). Background electrolyte MOPSO buffer (0.1 M, pH 6.7), Voltage of 30 kV, 25 °C.

HPLC chromatograms: Aquasil C18 column (150x4 mm, particle size 5 µm), isocratic eluation with 1.1 % acetonitrile in acetate buffer (50 mM, pH, 4.3, 0.1 M NH_4Cl).

Reaction components were detected by UV-detection at 268 nm in both methods.

Figures S19A-C: Electropherograms of samples from reaction of α-phosphate **6α** at pH 6.7 at 90 °C

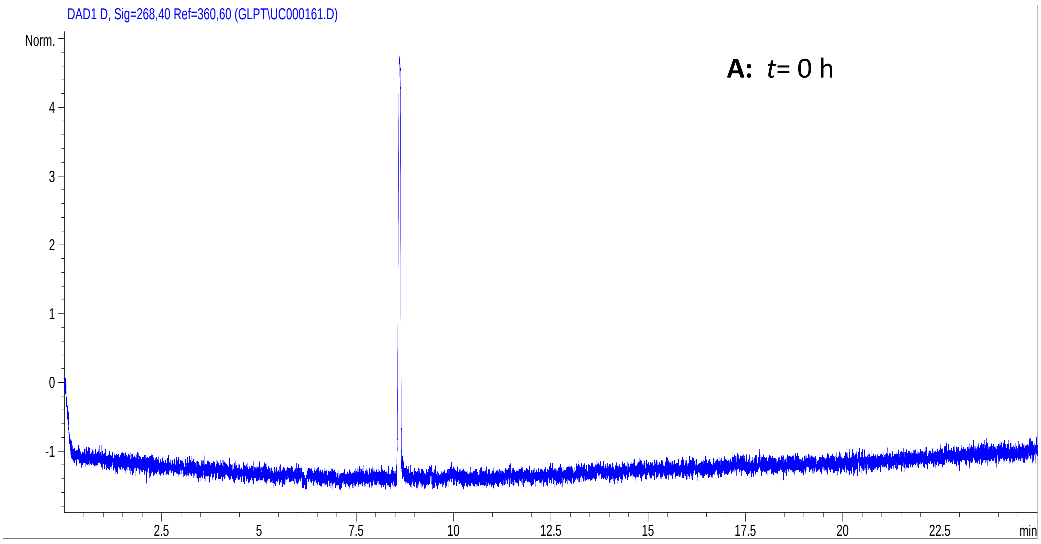

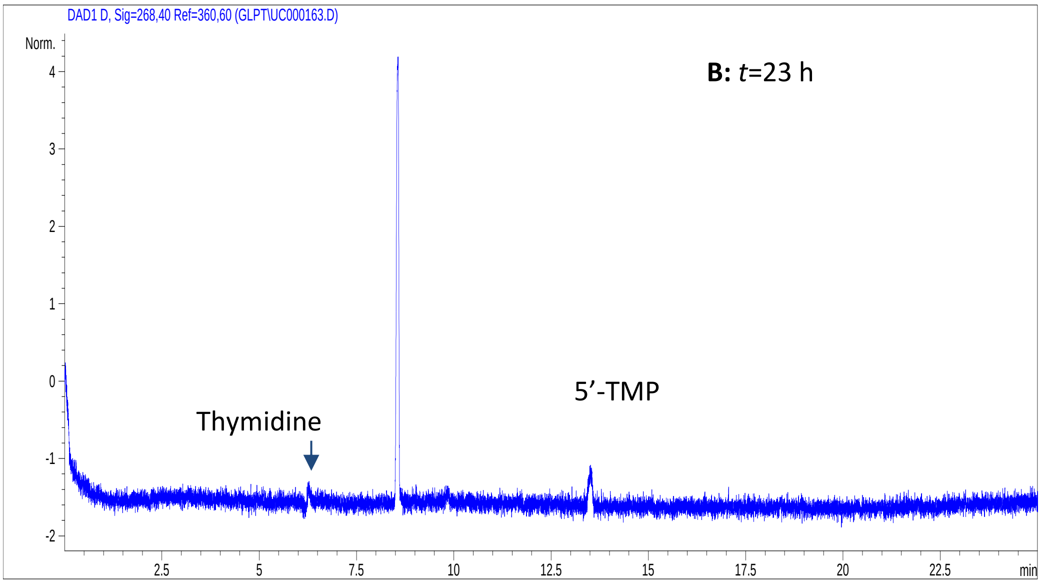

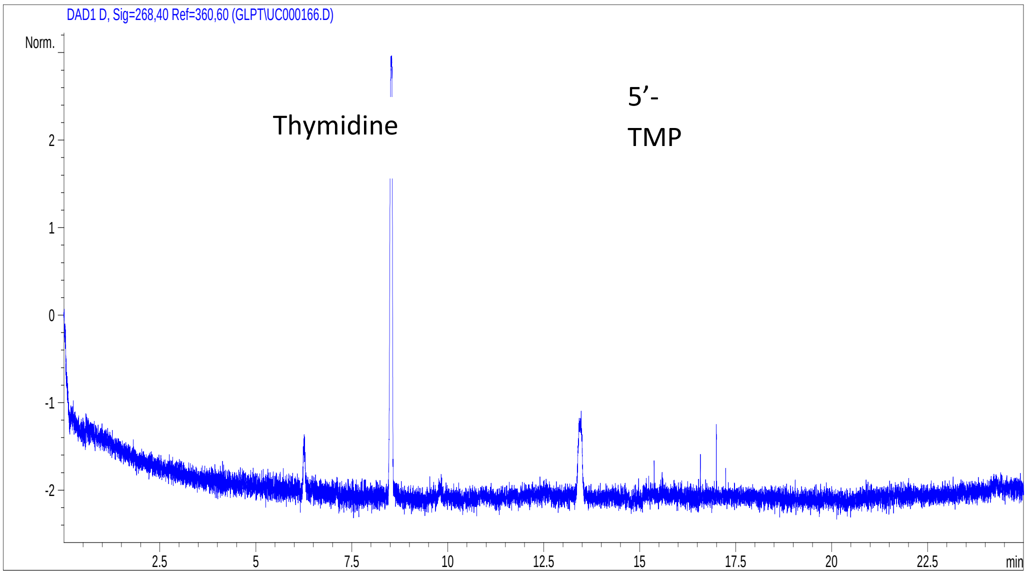

Figure S20: An electropherogram of a sample from the reaction of α-phosphate **6α** at pH 3.0 at 90 °C

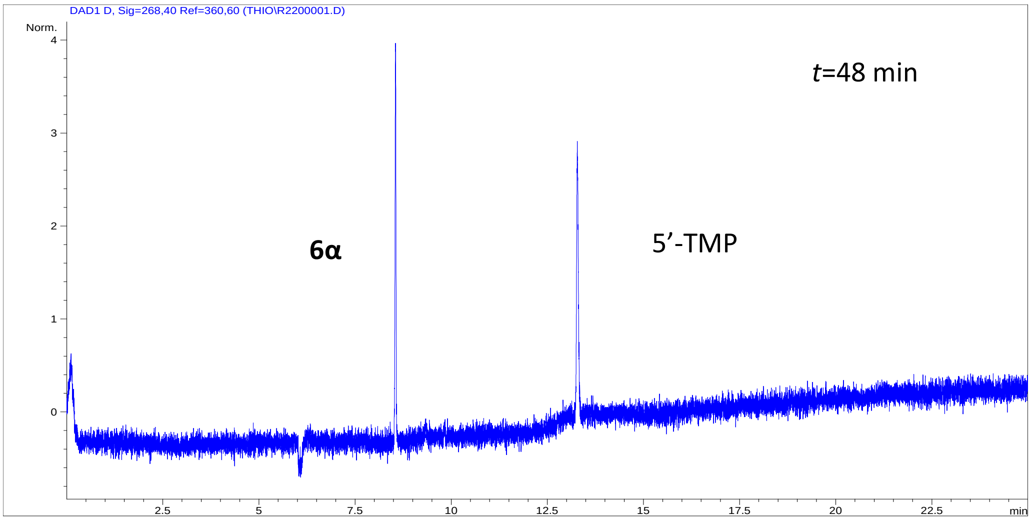

Figure S21A,B: HPLC-chromatograms of the reaction of **6α** with 10 mM CuBiPy at pH 6.7, 90 °C .

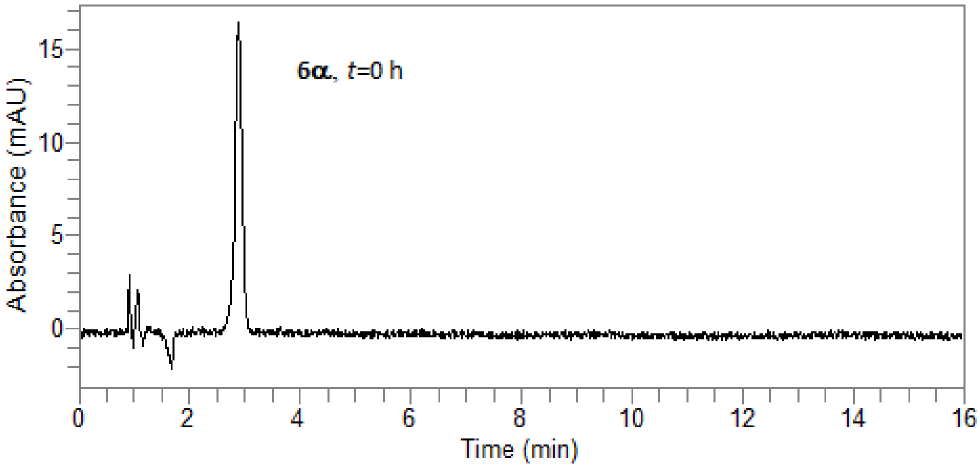

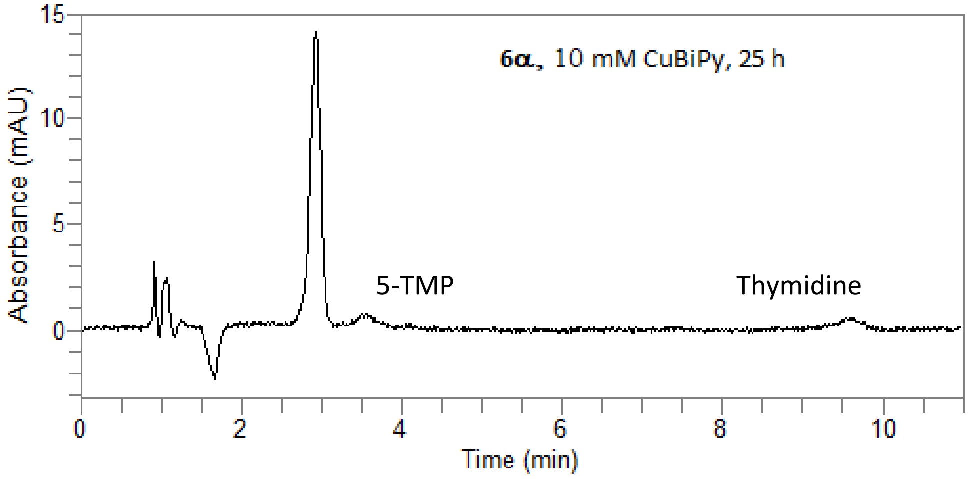

Figure S22: An HPLC-chromatogram of the reaction of α-phosphate **6α** with 10 mM CuTerPy at pH 6.7, 90 °C .

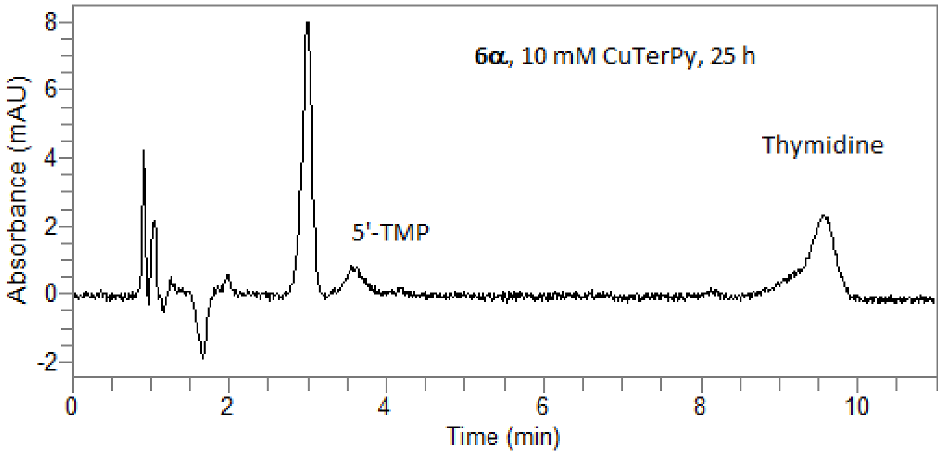

Figure S23: HPLC-chromatogram of TMP with 10 mM CuTerPy at pH 6.7, 20 h

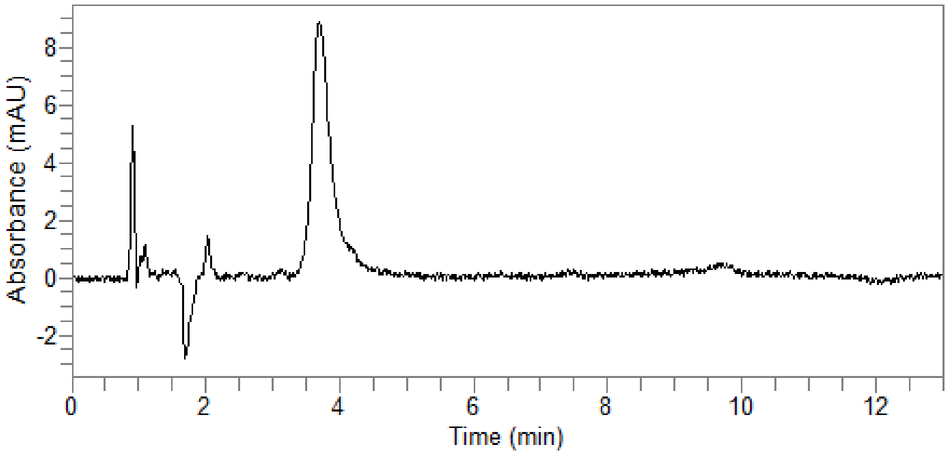

Figure S24: An electropherogram of a sample from a reaction of **7β2** at pH 3.0 to identify thio-TMP

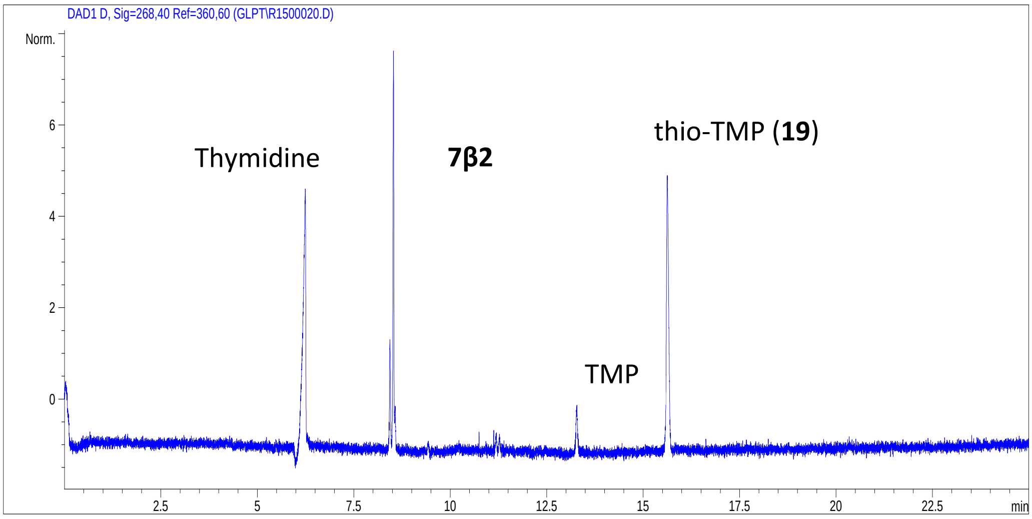

Figure S25: An electropherogram of a sample from a reaction of **7α2** at pH 6.7, 90 °C showing an extra peak close to the starting material

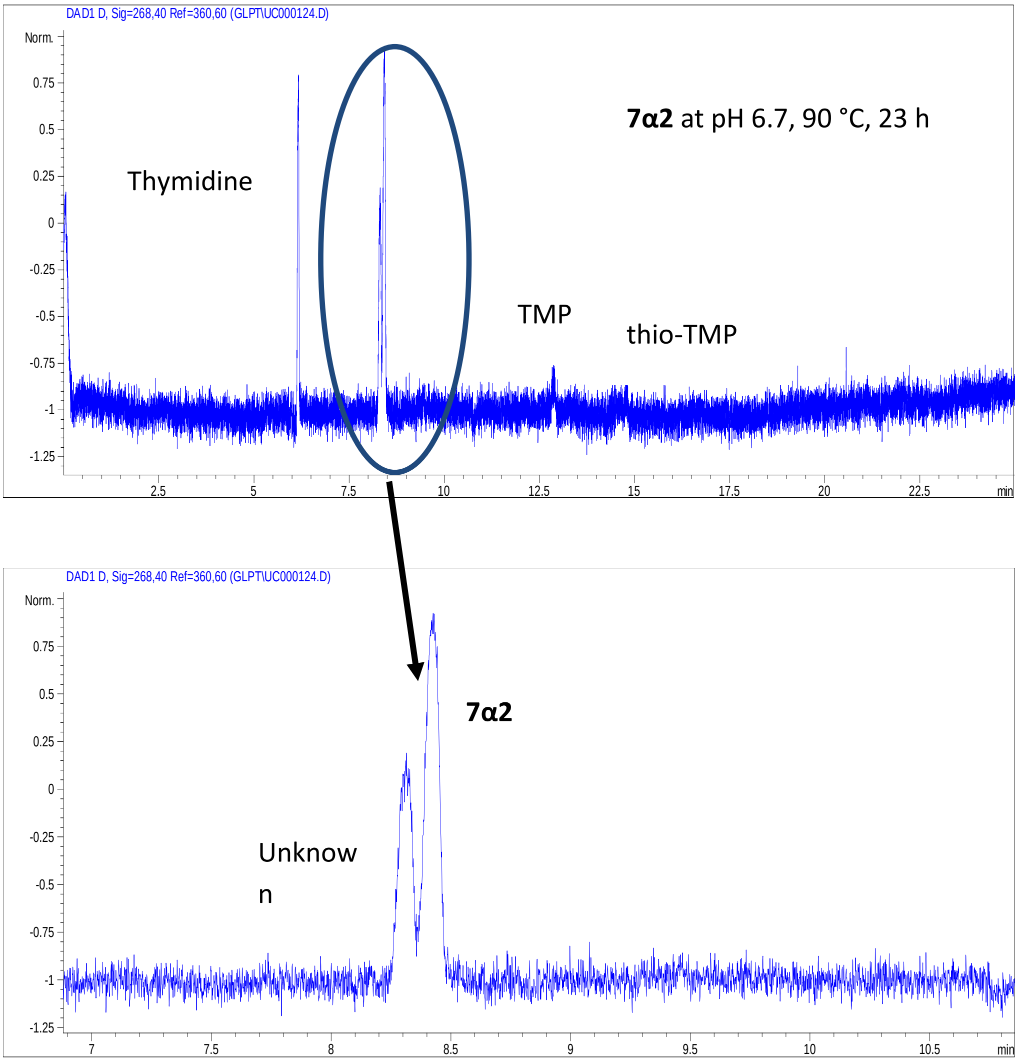

Figure S26A-C: HPLC-chromatograms of samples form reaction of **7α2** at pH 6.7 and 90 °C

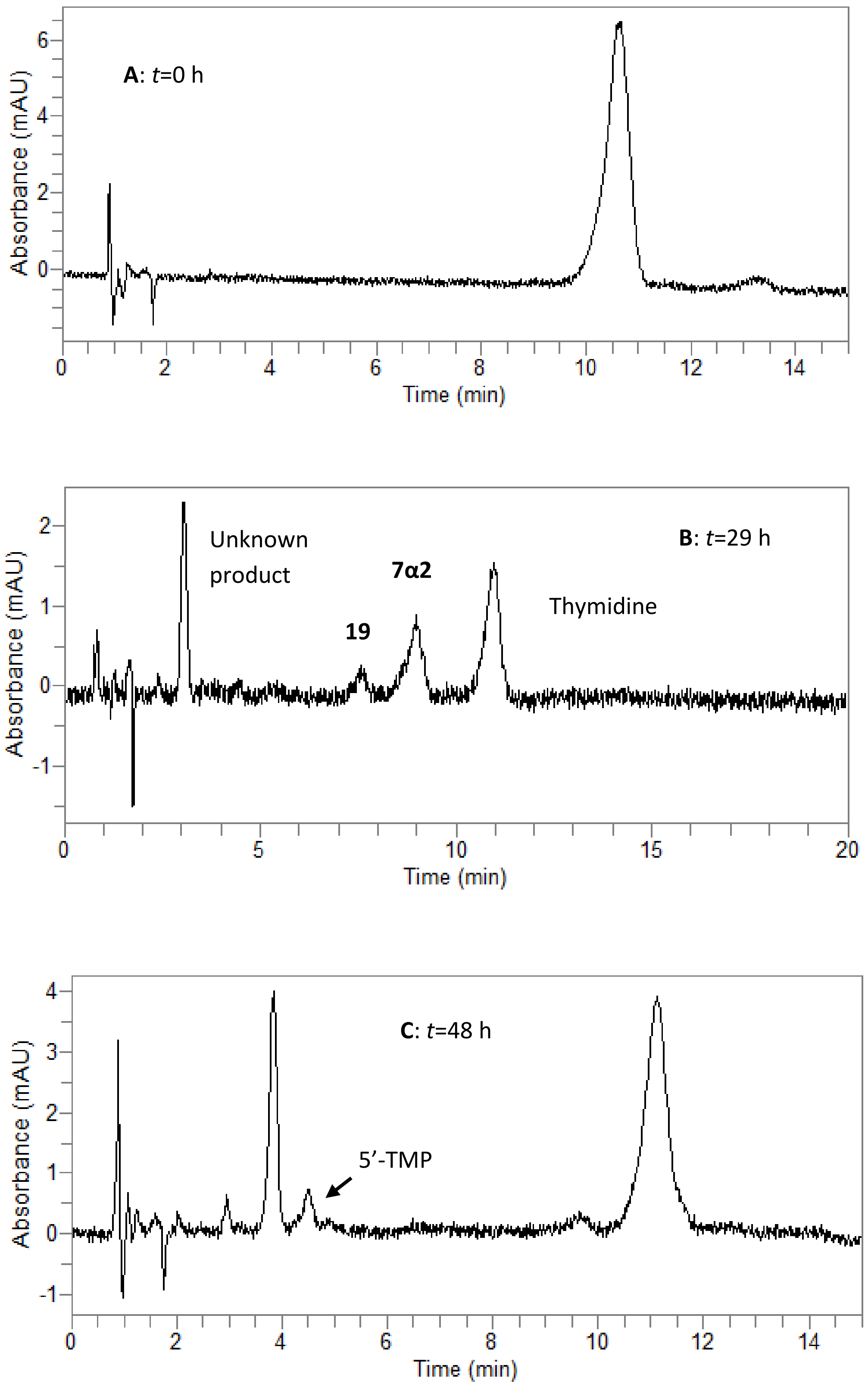

Figure S27A,B. HPLC-chromatograms of sample from reaction of **7α2** at pH 6.7 (29 h). A gradient elution is applied. In **B** α-phosphate is added in the sample to show that the unknown peak is not the corresponding phosphate.

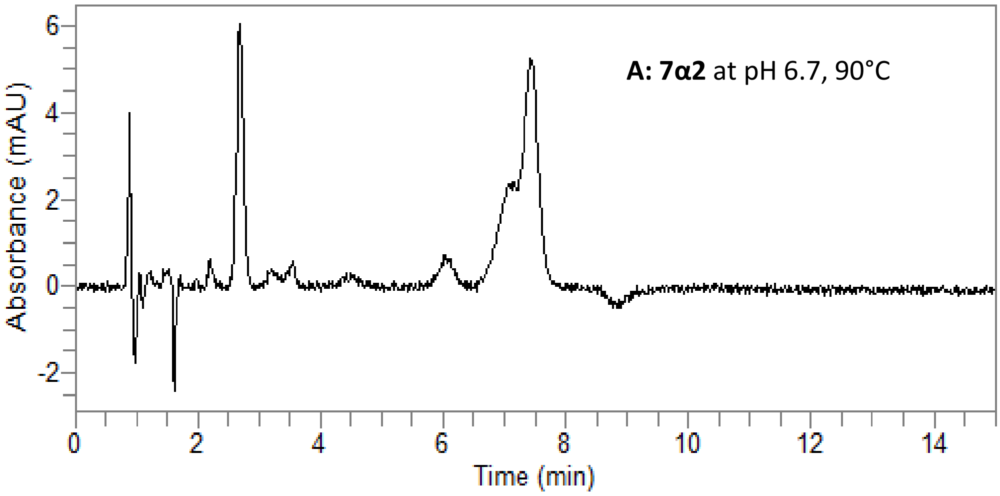

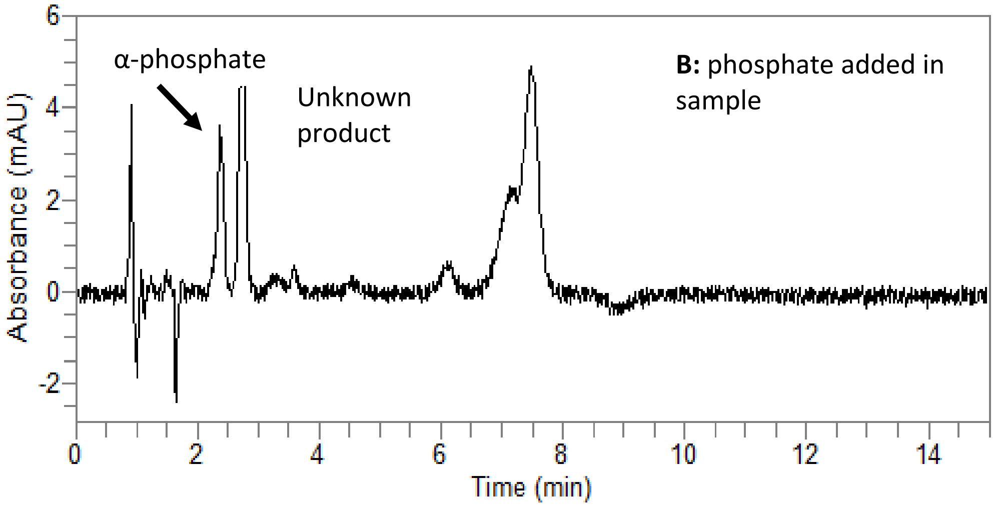

Figure 28A,B: HPLC-chromatograms of samples from the reaction of **7α2** with CuBiPy at pH 6.7 and 90 °C

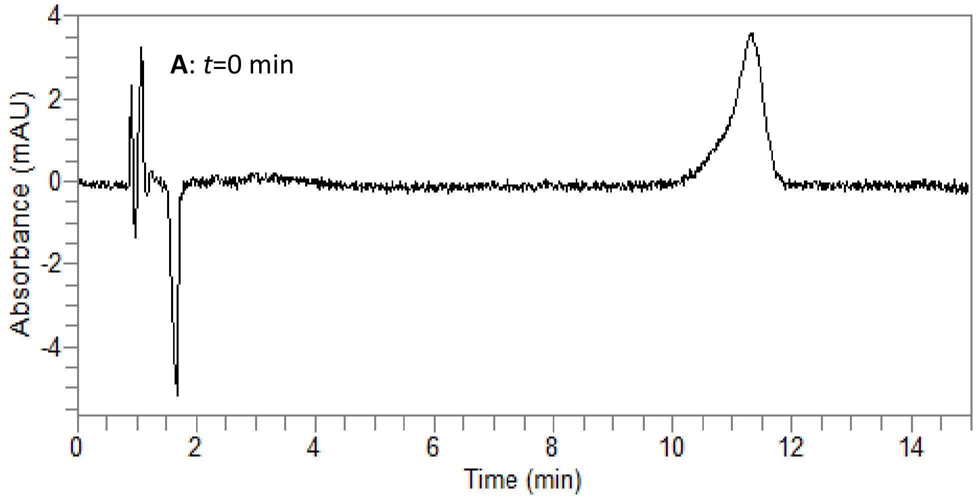

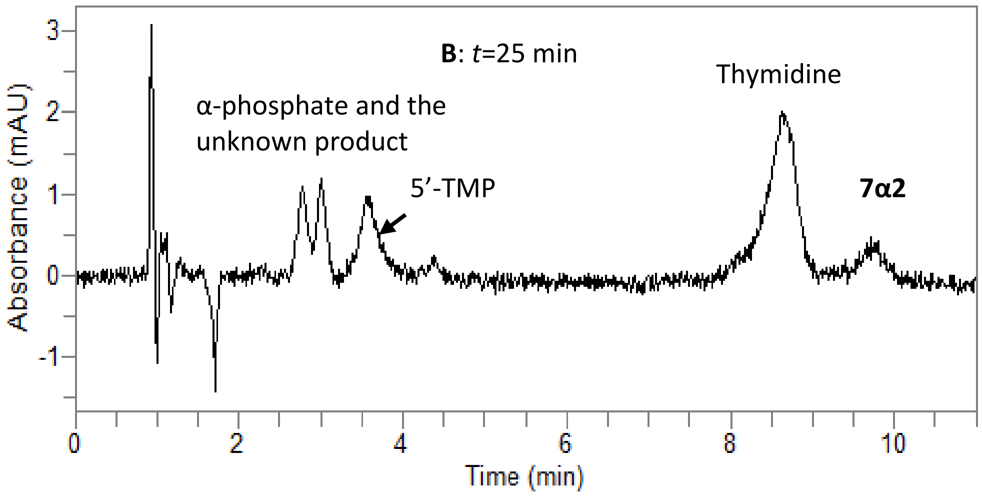

Figure **29A,B:** HPLC-chromatograms of samples from the reaction of **7α2** with CuTerPy at pH 6.7 and 90 °C

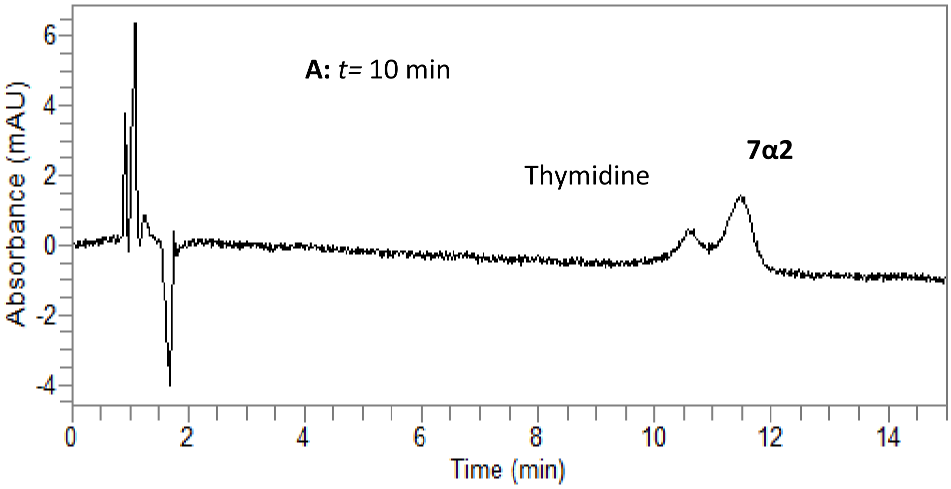

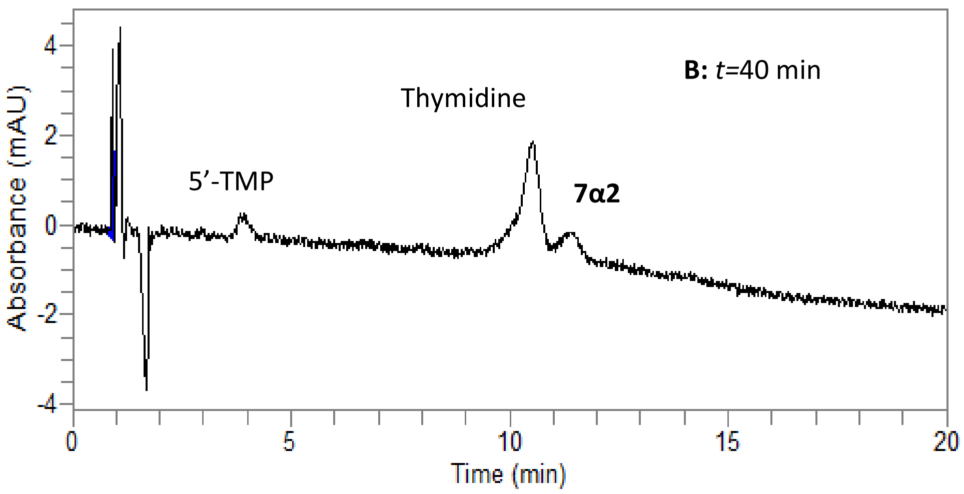
B: t=40 min
Thymidine
5'-TMP
7α2
Absorbance (mAU)
4
2
0
-2
-4
0
5
10
15
20
Time (min)
